# LncRNA PCNAP1 modulates hepatitis B virus replication and enhances tumor growth of liver cancer

**DOI:** 10.7150/thno.34273

**Published:** 2019-07-09

**Authors:** Jinyan Feng, Guang Yang, Yunxia Liu, Yuen Gao, Man Zhao, Yanan Bu, Hongfeng Yuan, Ying Yuan, Haolin Yun, Mingming Sun, Hongwei Gao, Shuqin Zhang, Zixian Liu, Ming Yin, Xijun Song, Zhenchuan Miao, Zhongqing Lin, Xiaodong Zhang

**Affiliations:** 1 Department of Cancer Research, College of Life Sciences, Nankai University, Tianjin 300071, China.; 2 Key Laboratory of Plant Resources and Chemistry in Arid Regions, Xinjiang Technical Institute of Physics and Chemistry, Chinese Academy of Sciences, Urumqi 830011, P.R. China.; 3 Beijing Vitalstar Biotechnology Co. Ltd.

**Keywords:** LncRNA PCNAP1, HBV, HBV cccDNA, PCNA, hepatocarcinogenesis.

## Abstract

**Rationale**: Hepatitis B virus (HBV) is a major risk factor for liver cancer, in which HBV covalently closed circular DNA (cccDNA) plays crucial roles. However, the effect of pseudogene-derived long noncoding RNAs (lncRNAs) acting as functional regulators of their ancestral gene expression on HBV replication and hepatocellular carcinoma (HCC) remains unclear. In this study, we speculated that the pseudogene-derived lncRNA PCNAP1 and its ancestor PCNA might modulate HBV replication and promote hepatocarcinogenesis.

**Methods**: We investigated the roles of lncRNA PCNAP1 in contribution of HBV replication through modulating miR-154/PCNA/HBV cccDNA signaling in hepatocarcinogenesis by using CRISPR/Cas9, Southern blot analysis, confocal assays, *et al.* in primary human hepatocytes (PHH), HepaRG cells, HepG2-NTCP cells, hepatoma carcinoma cells, human liver-chimeric mice model, transgenetic mice model, *in vitro* tumorigenicity and clinical patients.

**Results**: Interestingly, the expression levels of PCNAP1 and PCNA were significantly elevated in the liver of HBV-infectious human liver-chimeric mice. Clinically, the mRNA levels of PCNAP1 and PCNA were increased in the liver of HBV-positive/HBV cccDNA-positive HCC patients. Mechanistically, PCNA interacted with HBV cccDNA in a HBc-dependent manner. PCNAP1 enhanced PCNA through sponging miR-154 targeting PCNA mRNA 3′UTR. Functionally, PCNAP1 or PCNA remarkably enhanced HBV replication and accelerated the growth of HCC *in vitro* and* in vivo*.

**Conclusion**: We conclude that lncRNA PCNAP1 enhances the HBV replication through modulating miR-154/PCNA/HBV cccDNA signaling and the PCNAP1/PCNA signaling drives the hepatocarcinogenesis. Our finding provides new insights into the mechanism by which lncRNA PCNAP1 enhances HBV replication and hepatocarcinogenesis.

## Introduction

At least 350 million people worldwide are chronically infected with hepatitis B virus (HBV), a small hepatotropic DNA virus that selectively infects hepatocytes in human liver and replicates through reverse transcription, which is a leading cause of hepatitis, liver cirrhosis and liver cancer 1. Upon HBV infection, the viral relaxed circular DNA (rcDNA) genome in nucleocapsid is transported into the nucleus 2, 3 and converted into covalently closed circular DNA (cccDNA), which plays a key role in the viral persistence that is intractable of current antiviral therapies 4. HBV cccDNA is a template for the transcription of viral RNAs, being a nucleosome-bound minichromosome before 5, which is likely associated with the hepatitis B virus X protein (HBx) and key host factors mediated by epigenetic control 6, 7. Our recent work identifies that HBx-elevated MSL2 is able to modulate HBV cccDNA to enhance hepatocarcinogenesis through a positive feedback loop of HBx/MSL2/cccDNA/HBV 8. However, the host molecules that modulate HBV replication and cccDNA accumulation and lead to hepatocarcinogenesis are poorly understood.

Accumulating evidence has indicated that long non-coding RNAs (lncRNAs) can participate in diverse physiological and pathological processes and affect disparate cellular functions 9. LncRNAs are transcripts longer than 200 bp that do not have any apparent protein-coding ability 9. Advances in the depth and quality of transcriptome sequencing have revealed many new classes of lncRNAs, in which pseudogenes have been recently identified 10, 11. Basically, pseudogenes are ancestral copies of protein-coding genes that arise from genomic duplication or retro-transposition of mRNA sequences into the genome followed by accumulation of deleterious mutations due to the loss of selection pressure 12, degenerating eventually into so-called genetic fossils 13. As an important classification of lncRNA, pseudogenes play important roles in many cellular processes 9. It has been reported that a pseudogene long-noncoding-RNA network regulates PTEN transcription and translation in human cells 11.

PCNA, acting as a coordinator of DNA polymerase(s) in maintaining genomic integrity at both genetic and epigenetic levels, plays multiple roles in DNA replication and repair, and interacts with many partner proteins to accomplish these roles 14. PCNA as a cellular marker for proliferation can be used for grading of different neoplasms 14, including at least four valid pseudogenes, such as PCNAP1, PCNAP2, PCNAP3 (LOC392454) and PCNAP4 (LOC390102) 15. However, the effect of PCNA pseudogenes on its ancestor PCNA has not been reported. Notably, significance of PCNA pseudogenes in HBV infection and cancers has not been documented. In addition, lncRNAs may serve as endogenous RNAs that regulate each other through miRNA-dependent crosstalk 16, among which pseudogenes as the high sequence homology to their ancestral genes enable to share common microRNAs (miRNAs), leading to the regulation of ancestral genes 17. The expression of miR-154 is down-regulated in several types of cancers, including hepatocellular carcinoma, colorectal cancer, non-small cell lung cancer, breast cancer, and prostate cancer. MiR-154 acts as a tumor suppressor to inhibit the cell proliferation and metastasis 18. Importantly, the host-virus interaction is critical for the development of HBV-related liver cancer. However, the relationships among lncRNA PCNA pseudogenes, PCNA, miR-154, HBV and HCC are not well documented.

In this study, we are interested in the effect of lncRNA PCNA pseudogene on HBV replication and hepatocarcinogenesis. Surprisingly, we identify that the host lncRNA PCNAP1 enhances HBV replication through modulating miR-154/PCNA/HBV cccDNA signaling and the PCNAP1/PCNA signaling drives the hepatocarcinogenesis. Our finding provides new insights into the mechanism by which lncRNA PCNAP1 promotes HBV replication and hepatocarcinogenesis.

## Results

### LncRNA PCNAP1 promotes HBV replication and cccDNA accumulation

**Figure 1:**
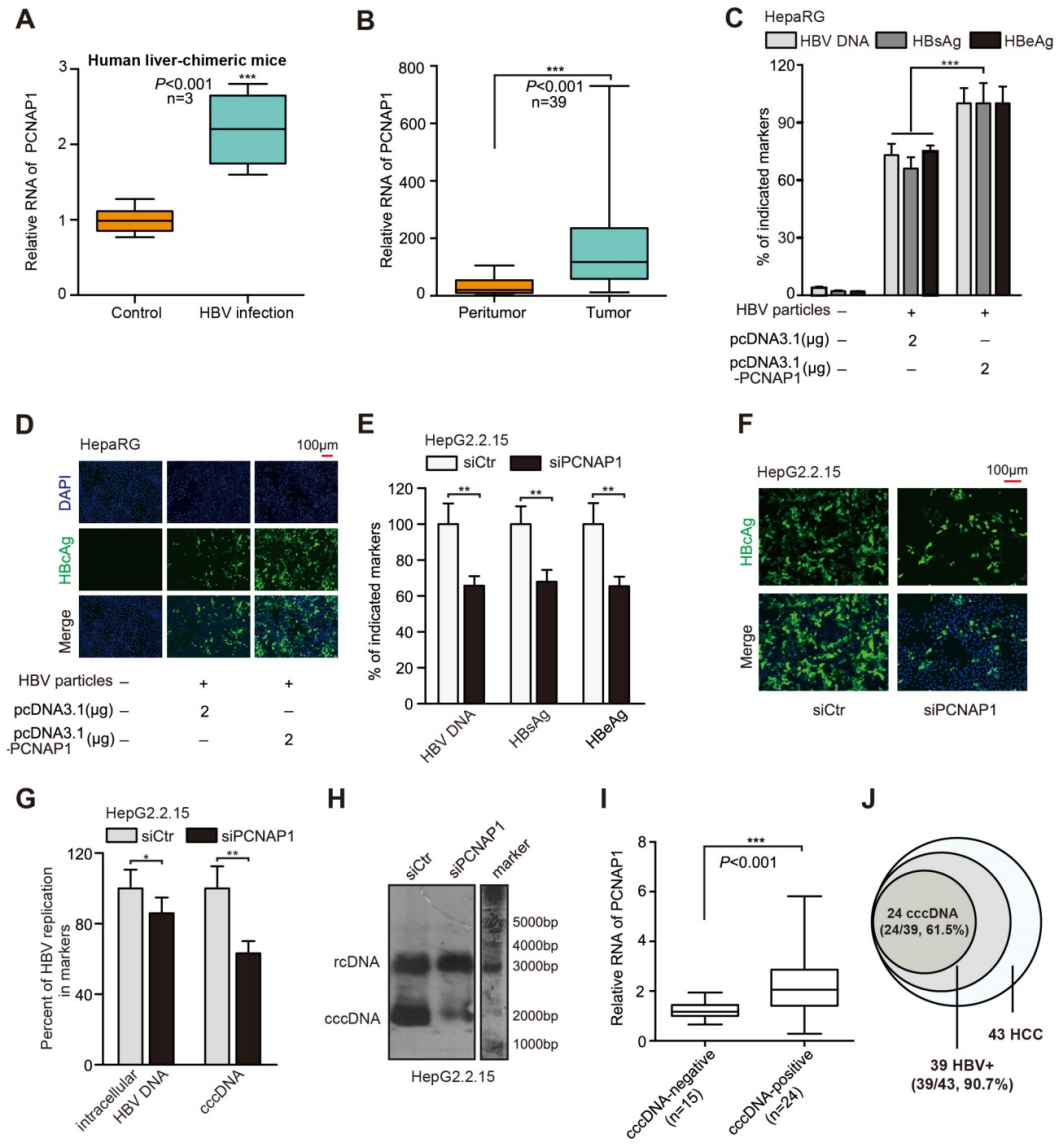
Pseudogene PCNAP1 promotes HBV replication and cccDNA accumulation. (A) The relative levels of PCNAP1 were determined by RT-qPCR in the liver of human liver-chimeric mice (n=3) and HBV-infected human liver-chimeric mice (n=3). (B) The relative levels of PCNAP1 were examined by RT-qPCR in 39 paired HBV-related HCC tissues and adjacent non-tumorous liver tissues (*P<0.001, Unpaired *t* test). (C and D) The levels of HBV progeny DNA, HBsAg, HBeAg and HBcAg were measured by qPCR, ELISA or immunofluorescence assays in HepaRG and HBV-infected HepaRG cells treated with pcDNA3.1 or pcDNA3.1-PCNAP1 at dpi 8 for 3 days. (E-H) HepG2.2.15 cells were treated with siCtr or siPCNAP1. (E and F) The levels of HBV progeny DNA, HBeAg, HBsAg and HBcAg were measured as above. (G and H) The intracellular HBV DNA and cccDNA were evaluated by qPCR and Southern blot assays, respectively. (I and J) Relative levels of PCNAP1 were detected by RT-qPCR in cccDNA-positive liver tissue of HCC patients (n=24) and cccDNA-negative ones (n=15). Statistical significant differences are indicated: *P<0.05; **P<0.01; ***P<0.001; Student's *t* test.

Basically, pseudogene-derived lncRNA as a novel tier of gene regulation functions in multiple cellular processes 12. It has been reported that four PCNA pseudogenes in human are named as PCNAP1, PCNAP2, PCNAP3 and PCNAP4, which are located on 4q23, 4q23, Xp11.3-Xp11.4, and 11p15.1 chromosome 15, respectively. Interestingly, our genome blast analyses showed that PCNAP1 especially displayed high homology to the entire mature PCNA mRNA, and had acquired a 344 bp sequence that showed 78% homology to the 3′UTR of PCNA transcript (Figure S1A). Using specific primers for each PCNA pseudogene, in this study all candidate pseudogenes were amplified by PCR from random or oligo-dT primed cDNA prepared from HepG2.2.15 total RNAs. The identity of PCNA pseudogenes was validated by PCR (Figure S1B). Using total RNAs from nuclear and cytoplasmic fractions of HepG2.2.15 cells, pseudogene-specific quantitative reverse transcription PCR (RT-qPCR) displayed that PCNAP1 was predominantly located in the cytoplasm, while PCNAP2, PCNAP3 and PCNAP4 mainly gathered in the nucleus (Figure S1C). To address whether the PCNA pseudogenes were subjected to HBV expression, we prepared total RNAs from HBV-negative (HBV-) hepatoma cells (HepG2, HepaRG and HepAD38) and HBV-expressing (HBV+) hepatoma cells (termed as HepG2*/HepaRG*/HepAD38* and HepG2.2.15). Interestingly, a 10-20 fold higher expression of PCNAP1 was observed in HBV+ hepatoma cells relative to HBV- ones. However, the other three PCNA pseudogenes showed irregular changes in the system (Figure S1D). Moreover, we examined the expression levels of PCNAP1 in human liver-chimeric mouse model. The levels of albumin and HBV DNA were shown (Table S1). Interestingly, we observed that the levels of PCNAP1 were significantly elevated in the livers from HBV-infected human liver-chimeric mice relative to those in control group (Figure 1A), suggesting that PCNAP1 is positively associated with HBV infection in liver. Additionally, our data revealed that the levels of PCNAP1 were significantly elevated in 39 HBV-related HCC patients relative to those in their adjacent non-tumorous liver tissues (Figure 1B), supporting that PCNAP1 may be positively related to HBV-related HCC.

Based on the quantitative analysis by using a more sensitive real-time PCR assay 19, we adopted four HBV-expressing cell lines and observed that HBV cccDNA and HBV concentration could be quantified and specifically detected (Figure S2A-B). Three days post transfection in HepaRG cells* de novo* infected with HBV particles, HBV indicated markers including HBV progeny DNA, HBsAg, HBeAg, HBcAg and HBx could be up-regulated by PCNAP1, respectively (Figure 1C-D, Figure S2C). Next, three siRNA sequences targeting PCNAP1 were designed, among which PCNAP1 siRNA3 showed the strongest knockdown efficiency (Figure S2D), then the PCNAP1 siRNA3 was used in the following experiments (termed siPCNAP1). Using qPCR, ELISA and immunofluorescence assays, we observed that the treatment of siPCNAP1 resulted in the decrease of HBV DNA, HBsAg, HBeAg and HBcAg in HepG2.2.15 cells, respectively (Figure 1E-F). Additionally, Western blot analysis and HBV-specific dot blot assays revealed that the expression levels of HBx protein and HBV progeny were attenuated in the cells (Figure S2E). Clinically, the levels of PCNAP1 were positively associated with those of HBx mRNA/pgRNA in 39 liver tissues from HBV-related HCC patients (Figure S2F).

Next, we found that the intracellular HBV DNA and HBV cccDNA displayed a remarkable up-regulation by qPCR and Southern blot assays in HepaRG cells transfected with PCNAP1 (Figure S2G-H). Moreover, we observed that the knockdown of PCNAP1 led to the opposite results in HepG2.2.15 cells (Figure 1G-H), indicating that PCNAP1 facilities HBV replication and cccDNA accumulation. Southern blot analysis showed the EcoRI digested-DNA and EcoRI undigested-DNA in HepaRG and HepG2.2.15 cells (Figure S2I-J), verifying the cccDNA and rcDNA. Then, we validated the role of PCNAP1 in modulating HBV replication and cccDNA accumulation by measuring HBV DNA and cccDNA in the PHH, HepaRG and HepG2.2.15 (Figure S2K-N). We further evaluated the relationships between PCNAP1 and HBV cccDNA in clinical liver tissues. Our data showed that 39 samples were positive for HBV DNA in 43 clinical liver cancer tissues, in which 24 samples (24/39) were positive for cccDNA. Interestingly, the expression levels of PCNAP1 were remarkably higher in cccDNA-positive tissues than those in cccDNA-negative ones (Figure 1I), supporting that PCNAP1 is positively associated with cccDNA in liver. Thus, our data that the incidence (24/39, 61.5%) of HBV cccDNA in 39 HBV-related HCC patients (Figure 1J) imply that HBV cccDNA may be associated with the development of HCC. Therefore, we conclude that the pseudogene PCNAP1 promotes HBV replication and cccDNA accumulation in liver cancer.

### PCNAP1 contributes to HBV replication and cccDNA accumulation through its ancestor PCNA

**Figure 2:**
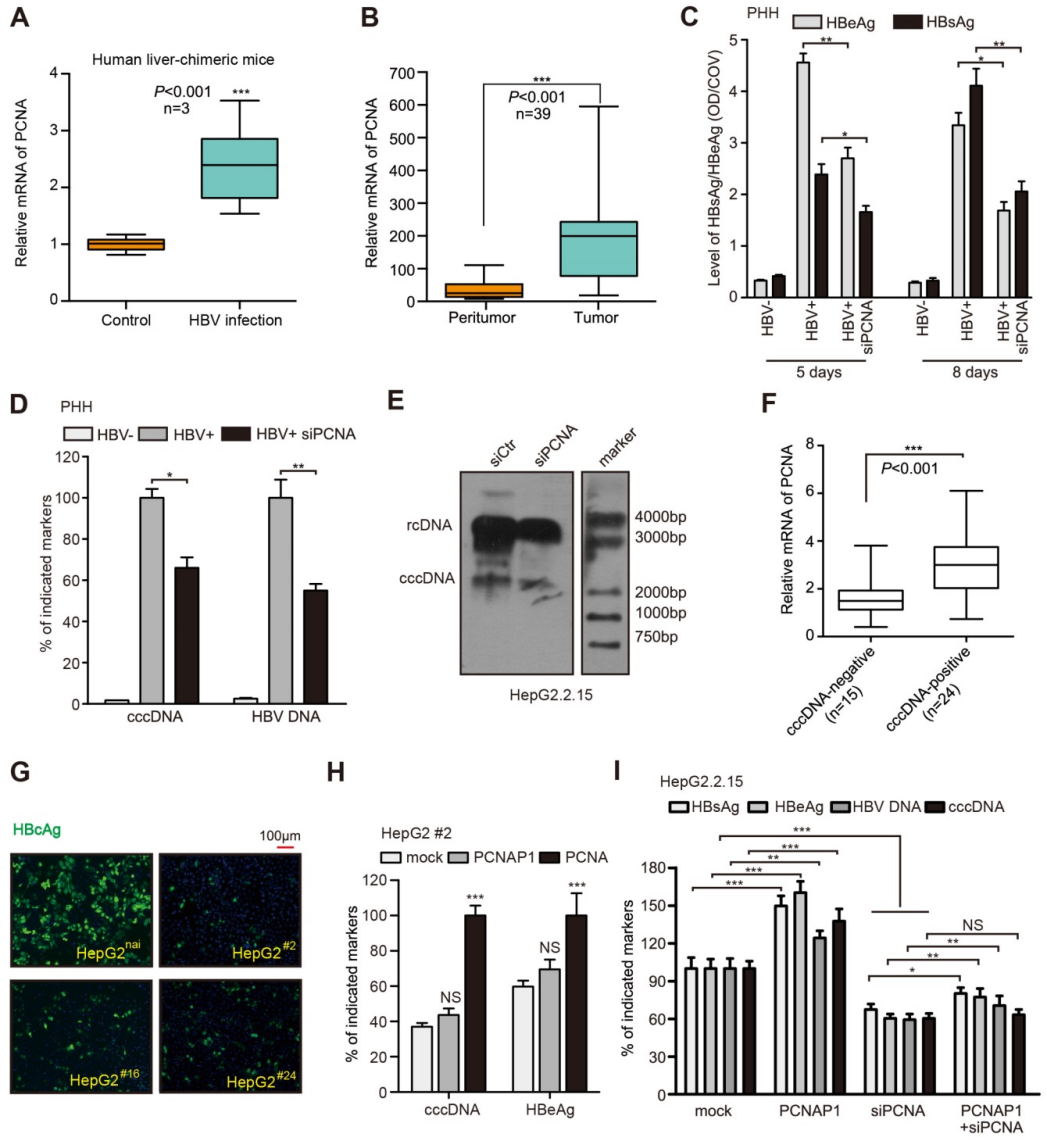
PCNAP1 contributes to HBV replication and cccDNA accumulation through its ancestor PCNA. (A) The mRNA levels of PCNA were assessed by RT-qPCR in the liver of human liver-chimeric mice (n=3) and HBV-infected human liver-chimeric mice (n=3). (B) The relative levels of PCNA were examined by RT-qPCR in 39 paired HBV-related HCC tissues and adjacent non-tumorous liver tissues (*P<0.001, Unpaired *t* test). (C and D) Primary human hepatocytes (PHH) were infected with HBV or without HBV particles and transfected with siRNA targeting PCNA or a negative control siRNA post infection. Indicated HBV markers including HBeAg, HBsAg, HBV DNA and cccDNA were measured by ELISA assays and qPCR, respectively. (E) HBV cccDNA were analyzed by Southern blot assays in HepG2.2.15 cells transfected with sicontrol (siCtr) or siPCNA. (F) Relative mRNA levels of PCNA were detected by RT-qPCR in cccDNA-positive liver cancer tissues (n=24) and cccDNA-negative ones (n=15). (G) Parental HepG2 (HepG2^nai^) and HepG2^PCNA-/-^ (HepG2^ #^) were treated with PCH9-3091 plasmids. The expression of HBcAg was detected 3 days post transfection. (H) The levels of HBV cccDNA and HBeAg was measured in HepG2 #2 cells transfected with pcDNA 3.1-PCNAP1 or pcDNA 3.1-PCNA. (I) The indicated markers were assessed by ELISA assays or qPCR in HepG2.2.15 cells transfected with pcDNA 3.1-PCNAP1, or siPCNA, or cotransfected with pcDNA 3.1-PCNAP1 and siPCNA, respectively. Error bars represent means ± SD (n=3). Statistical significant differences are indicated: *P<0.05; **P<0.01; ***P<0.001; NS, no significance; Student's *t* test.

To better understand the mechanism by which PCNAP1 modulated HBV replication and cccDNA accumulation, we performed gene expression microarray analysis in HepG2.2.15 cells treated with siPCNAP1. Interestingly, our data showed that 189 mRNAs were up-regulated and 1231 mRNAs were down-regulated in the cells (Figure S3A). Gene ontology (GO) function enrichment analysis demonstrated the response genes of PCNAP1 in the cellular biological processes (Figure S3B), including DNA repair, DNA replication, cell cycle checkpoint and p53 pathways. Some key and obvious-changed genes were shown in the heat map (Figure S3C). The expression levels of above response genes were validated by RT-qPCR in the cells (Figure S3D). Additionally, we observed that the expression levels of DNA repair factors, such as PCNA, CHK1, CHK2, PARP1, ATM and ATR, were highly expressed in HBV-infected HepaRG cells (Figure S3E). Basically, pseudogenes play multiple roles through modulating their ancestral genes 13. We observed that knockdown of PCNA was mostly able to decrease the levels of HBV cccDNA among the above 6 DNA repair factors (Figure S4A). The silencing efficiency of above 6 shRNA sequences was validated by RT-qPCR in HepG2.2.15 cells (Figure S4B). Moreover, we found that the mRNA levels of PCNA were significantly elevated in the liver of HBV-infected human liver-chimeric mice relative to those in control of human liver-chimeric mice (Figure 2A). Moreover, TCGA data analysis revealed that PCNA was highly elevated in the clinical HCC tissues and HBV-related HCC tissues (Figure S4C-D). As expected, we further demonstrated that PCNA was highly expressed in clinical HBV-related HCC tissues and the liver tissues from HBV transgenic (HBV-Tg) mice (Figure 2B, Figure S4E). The similar data were observed in 4 HBV-expressing cell lines at the levels of mRNA and protein (Figure S4F), suggesting that PCNA is associated with HBV infection in HCC.

Next, the effect of PCNA on HBV was further investigated. We adopted siRNAs targeting PCNA mRNA and chose siPCNA-2 (termed siPCNA) for further experiments. The *de novo* infection was performed in PHH cells using HBV particles, in which the cells were co-transfected with siPCNA at 16 hours post infection. As expected, we observed that siRNAs targeting PCNA mRNA could efficiently silence the secretion of HBeAg and HBsAg in PHH cells on day 5 and 8 post treated, respectively (Figure 2C). Interestingly, silencing of PCNA mRNA could significantly decrease the levels of cccDNA and HBV DNA in HBV-infected PHH cells (Figure 2D). The overexpression of PCNA was able to increase the secretion of HBsAg and HBeAg, as well as the quantification of intracellular HBV DNA and cccDNA in HepaRG cells *de novo* infected with HBV particles (Figure S4G). The similar extent changes of HBx expression levels, the amount of HBV progeny virion and the HBcAg levels were also observed in the cells (Figure S4H-I). Additionally, PCNA knockdown led to the suppression of HBeAg secretion by ELISA assays, 43.2% reduction of cccDNA levels and approximately 50% down-regulation in HBV DNA levels by qPCR analysis in HepG2.2.15 cells (Figure S4J).

And the levels of HBx and HBV progeny were also reduced in the PCNA knockdown cells (Figure S4K). Surprisingly, Southern blot assays showed that both rcDNA and the cccDNA could be remarkably decreased by siPCNA in HepG2.2.15 cells (Figure 2E), suggesting that PCNA is able to enhance the HBV replication and cccDNA accumulation. Then, we validated the role of PCNA overexpression or silencing in HBV replication and cccDNA accumulation by examining HBV DNA and cccDNA in the cell lines of PHH, HepaRG and HepG2.2.15 (Figure S2L-N). Clinically, we further evaluated the relationships between PCNA and HBV cccDNA in the liver tissues. Our data showed that 39 samples were positive for HBV DNA in 43 clinical liver cancer tissues, in which 24 samples were positive for HBV cccDNA. Interestingly, the expression levels of PCNA mRNA were remarkably higher in HBV cccDNA-positive tissues than those in HBV cccDNA-negative ones (Figure 2F), supporting that PCNA is associated with HBV cccDNA in liver.

Next, we took advantage of the CRISPR/Cas9 system to generate PCNA knockout in HepG2 cells 4, 20, 21. Western blot analysis identified that the expression levels of PCNA were efficiently silenced in 6 single clones including #2, #8, #16, #24, #29, #36 from 36 candidates. (Figure S5A). Moreover, we found that the levels of HBV cccDNA and HBeAg were remarkably decreased in #2, #16, #24 cell lines compared with those in the native HepG2 cells transfected with PCH9-3091 plasmid (an HBV 1.1× overlength genome) (Figure S5B-C). Meanwhile, Sanger sequencing analysis validated that the three clones had a frame shift in the coding region, which resulted in the disruption of intact PCNA protein expression (Figure S5D), suggesting that PCNA is homologous knockout in the three clones. Furthermore, we observed that PCNA knockout markedly reduced the levels of HBcAg by immunofluorescence assays in above 3 cell lines relative to those in the native HepG2 cells transfected with PCH9-3091 plasmids (Figure 2G). Then, we identified that the levels of HBV cccDNA were significantly decreased by qPCR assays in above system (Figure S5E). Interestingly, the effects could be mostly rescued by the re-expression of pcDNA3.1-PCNA in the cells (Figure S5E), implying that PCNA is involved in the event of cccDNA accumulation. Next, we found that the ectopic expression of pseudogene PCNAP1 failed to revert the depressed state of HBeAg and HBV cccDNA in #2 cell line transfected with PCH9/3091. But the re-expression of PCNA could sharply re-activate the levels of HBeAg and HBV cccDNA (Figure 2H), suggesting that the pseudogene PCNAP1 functions in promotion of HBV replication and cccDNA accumulation through its ancestor PCNA. Similarly, silencing of PCNA exhibited dramatic inhibition of HBV cccDNA and approximately 40-50% reduction of HBV replication markers including HBsAg, HBeAg and HBV DNA in HepG2.2.15 cells, in which the effect could be partially rescued by the ectopic expression of PCNAP1 (Figure 2I), supporting that PCNAP1 elevates the HBV replication and cccDNA accumulation through its ancestor PCNA. Thus, we conclude that PCNAP1 confers to the HBV replication and cccDNA accumulation through its ancestor PCNA.

### PCNA anchors onto cccDNA minichromosome through interacting with HBc

**Figure 3:**
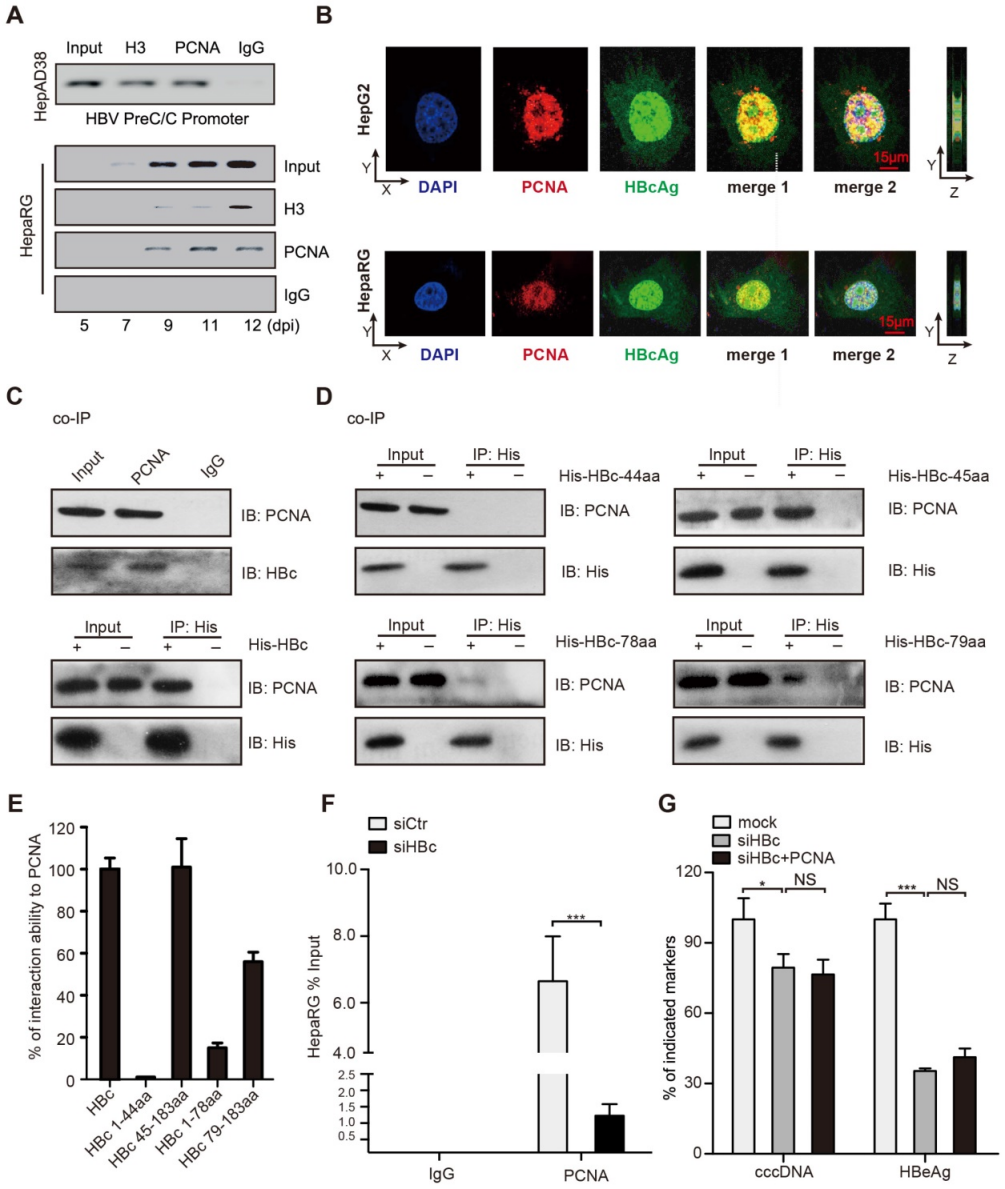
PCNA anchors onto cccDNA minichromosome through interacting with HBc. (A) Upper: cross-linked chromatin from HepAD38 cells treated without tetracycline was immunoprecipitated and analyzed by PCR. Bottom: kinetics of histone H3 and PCNA recruitment onto the HBV cccDNA in infected HepaRG cells. (B) In HepG2 cells transfected with PCH9/3091 plasmids and HBV-infected HepaRG cells, the colocalization of PCNA and HBc was analyzed by immunofluorescence. Right panels indicate Z stacks taken at the dotted lines. (C) Upper: the interaction between PCNA and HBc was identified in HepG2.2.15 cells. Bottom: in HepG2 cells cotransfected with PCH9/3091 plasmids and His-HBc, the interaction between PCNA and HBc was identified by co-IP assays. (D and E) Serial HBc deletion mutants were fused to His protein and interaction with PCNA was assessed by co-IP assays in HepG2 cells. (F) HepaRG cells were infected with HBV particles and transfected with siCtr or siHBc. The levels of cccDNA were analyzed by ChIP-qPCR. (G) HBV indicated markers including cccDNA and HBeAg were measured as above in HepG2.2.15. Error bars represent means ± SD (n=3). Statistical significant differences are indicated: ***P<0.001; NS, no significance; Student's* t* test.

Next, we explored the mechanism by which PCNA promoted HBV replication and cccDNA accumulation. It has been reported that PCNA as a lariat anchors on DNA strains to maintain its genome integrity 22. Surprisingly, cccDNA-ChIP assays showed that PCNA could bind to cccDNA in HepAD38 cells (tet off) (Figure 3A, up panel). The kinetics of PCNA recruitment onto cccDNA was tested by cccDNA-ChIP assays in HepaRG cells *de novo* infected with HBV particles (Figure 3A, bottom panel). We found that the recruitment of PCNA onto the cccDNA was most stable at 11 days post infection (Figure 3A, below panel), indicating that PCNA is able to anchor onto cccDNA minichromosome.

Given that HBc as a guider protein initiated the cccDNA accumulation and HBV replication 23, we wondered whether PCNA interacted with HBc in the event. Immunofluorescence assays indicated that PCNA was mainly distributed in the nucleus and partially co-localized with the nucleus-distributed HBc in HBV infected-HepaRG cells and in HepG2 cells transfected with PCH9-3091 plasmids (Figure 3B). Furthermore, co-IP assays validated that either ectopically expressed HBc or endogenous HBc was able to interact with PCNA in the cells (Figure 3C). To identify the binding domain of HBc with PCNA, we constructed serial HBc mutants into the plasmids containing 6xHis at C-terminus (Figure S6A) and determined that the region located at amino acids 44 to 183 of HBc was responsible for the interaction with PCNA (Figure 3D-E). In addition, co-IP assays showed that PCNA with depletion of protein binding domain could still interact with HBc (Figure S6B), suggesting that the protein binding domain of PCNA is not necessary for the interaction of PCNA with HBc. Importantly, cccDNA-ChIP assays validated that PCNA with depletion of DNA binding domain failed to bind to HBV cccDNA (Figure S6C), supporting that DNA binding domain (amino acids 61-80) of PCNA is responsible for the interaction with HBV cccDNA. Thereby, we speculated whether the interaction of PCNA with HBc played important roles in the modulation of HBV replication. Functionally, cccDNA-ChIP assays showed that the capability of PCNA binding to cccDNA was inhibited by siRNA of HBc in HBV infected-HepaRG cells (Figure 3F). Consistently, the siRNA of HBc significantly decreased the levels of HBV cccDNA and HBeAg in the system. However, the overexpression of PCNA failed to affect the event (Figure 3G), suggesting that HBc recruits PCNA to HBV cccDNA minichromosome, leading to HBV replication and HBV cccDNA accumulation.

To better understand the interaction of HBc with PCNA, we generated a three-dimensional co-crystallization structure by automatic modeling mode. The potential interaction sites (yellow) of modeled PCNA and HBc were depicted as Figure S6D. The main domain of PCNA interacting with HBc was located in the α-helix, but not the classical protein binding domain (IDCL). Simultaneously, the main domain of HBc interacting with PCNA was located in the α-helix of C-terminus. Thus, we conclude that PCNA anchors onto cccDNA minichromosome in a HBc-dependent manner, leading to HBV replication and cccDNA accumulation.

### PCNAP1 stimulates the expression of PCNA at post-transcriptional level

**Figure 4:**
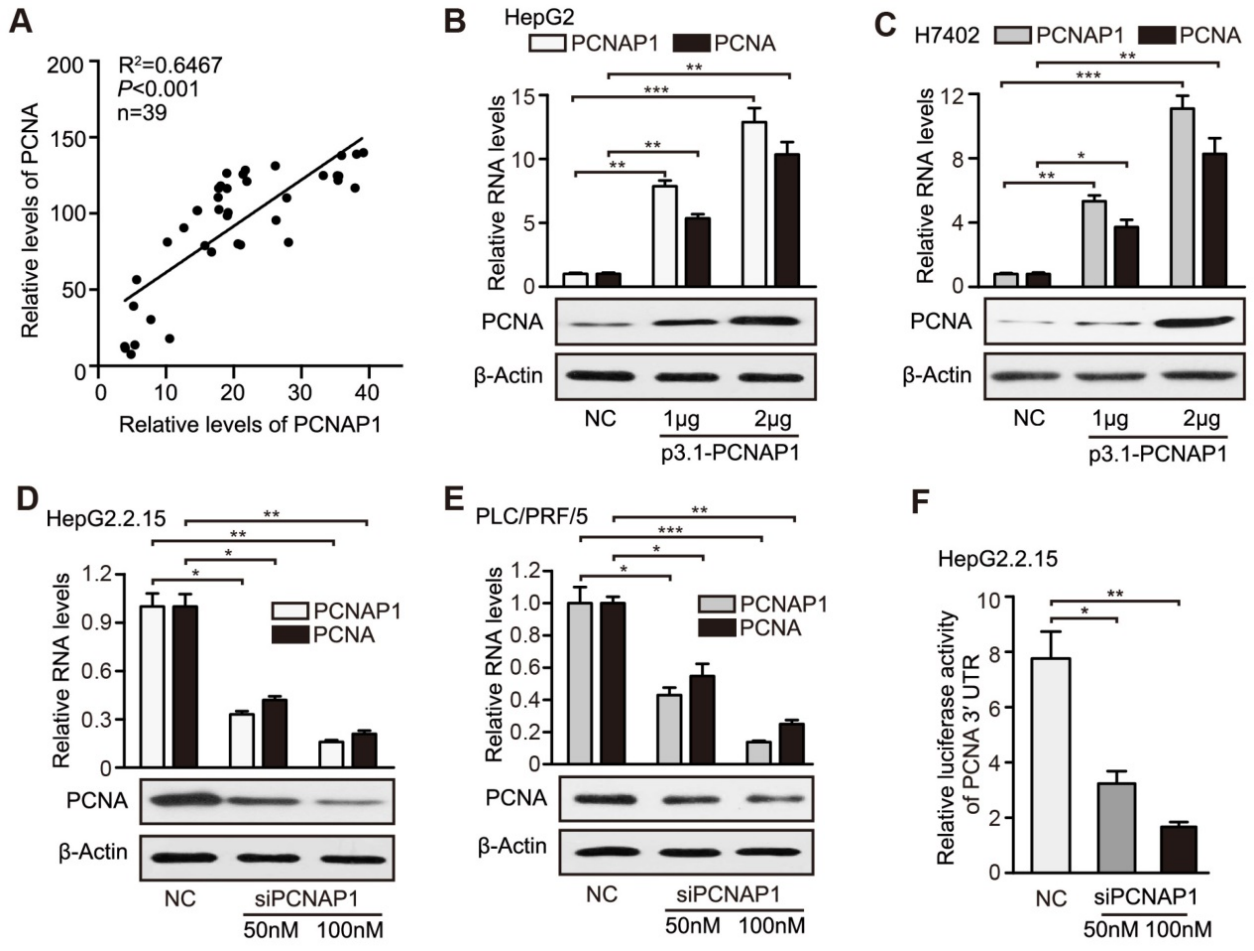
PCNAP1 stimulates the expression of PCNA at post-transcriptional level in hepatoma cells. (A) Correlation between the PCNAP1 levels and the PCNA mRNA levels was examined by RT-qPCR in 39 cases of HBV-related HCC tissues. (B and C) The expression of PCNA was detected by RT-qPCR and Western blot analysis in HepG2 and H7402 cells transfected with pcDNA3.1-PCNAP1. (D and E) The expression of PCNA was detected as above in HepG2.2.15 and PLC/PRF/5 cells transfected with siPCNAP1. (F) Luciferase activities of PCNA mRNA 3′UTR were measured by luciferase reporter gene assays in HepG2.2.15 cells transfected with siPCNAP1. Error bars represent means ± SD (n=3). Statistical significant differences are indicated: *P<0.05; **P<0.01; ***P<0.001; Student's *t* test.

Given that the pseudogenes played multiple roles through modulating their ancestral genes 12, we concerned whether the pseudogene PCNAP1 modulated its ancestor PCNA in hepatoma cells. Interestingly, RT-qPCR assays showed that the RNA levels of PCNAP1 were positively associated with those of ancestral PCNA transcripts in clinical HBV-related HCC liver tissues (Figure 4A). Similarly, we demonstrated the positive correlation between the expressions of PCNAP1 and PCNA in 10 liver cell lines by RT-qPCR and Western blot analysis, respectively. (Figure S7A-B). Notably, our data showed that the overexpression of PCNAP1 could significantly elevate the levels of mRNA and protein of PCNA in HepG2 and H7402 cells in a dose-dependent manner (Figure 4B-C). Additionally, the knockdown of PCNAP1 dose-dependently decreased the expression levels of PCNA in HepG2.2.15 and PLC/PRF/5 cells (Figure 4D-E), indicating that PCNAP1 is able to up-regulate PCNA in liver cancer. To better understand the mechanism by which PCNAP1 modulated the expression of PCNA, we constructed the PCNA promoter region and PCNA mRNA 3′UTR into the pGL3-basic and pGL3-control vectors, respectively. Luciferase reporter gene assays indicated that siPCNAP1 could decrease the luciferase activities of PCNA mRNA 3′UTR, rather than those of PCNA promoter in HepG2.2.15 cells (Figure 4F, Figure S7C), suggesting that PCNAP1 regulates its ancestral PCNA at post-transcriptional level. Therefore, we conclude that PCNAP1 enhances the expression of PCNA at post-transcriptional level in liver.

### PCNAP1 up-regulates PCNA through competing for miR-154

**Figure 5:**
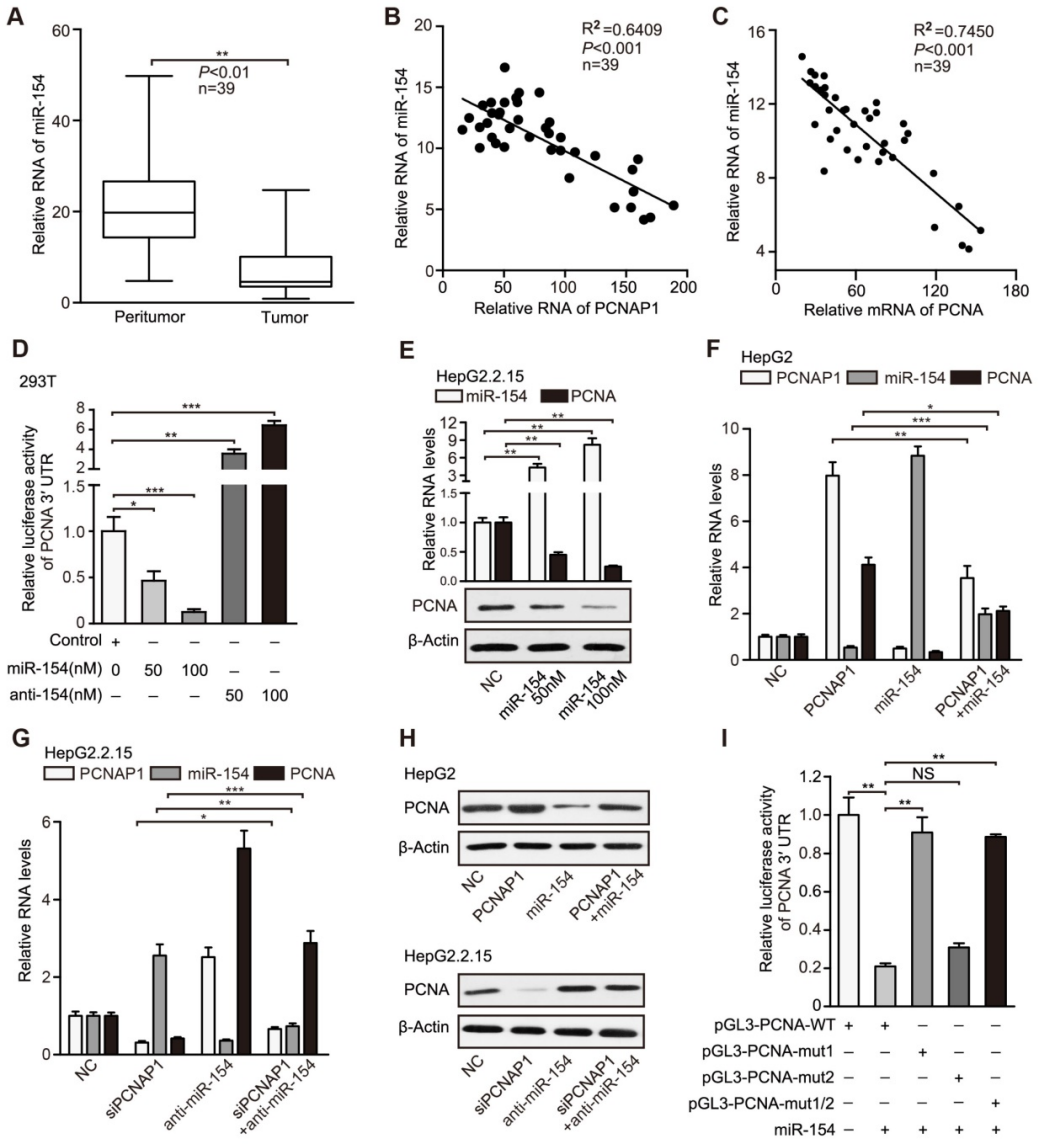
PCNAP1 up-regulates PCNA through competing for miR-154. (A) The relative levels of miR-154 were examined by RT-qPCR in clinical HBV-related HCC samples. (B and C) The correlation between the PCNAP1 or PCNA mRNA levels and the miR-154 levels was examined by RT-qPCR in clinical HBV-related HCC samples. (D) Luciferase activities of PCNA mRNA 3′UTR were measured by luciferase reporter gene assays in 293T cells transfected with miRNA control, miRNA-154 or miRNA-154 inhibitors. (E) The expression of miR-154 and PCNA was detected by RT-qPCR and Western blot analysis in HepG2.2.15 cells transfected with miRNA control or miRNA-154. (F) The RNA levels of PCNAP1, miR-154 and PCNA were measured by RT-qPCR analysis in HepG2 cells with the single treatment of PCNAP1 or miR-154 and the co-transfection with PCNAP1 and miR-154. (G) The RNA levels of PCNAP1, miR-154 and PCNA were tested by RT-qPCR analysis in HepG2.2.15 cells with the single treatment of siPCNAP1 or anti-miR-154 and the co-transfection with siPCNAP1 and anti-miR-154. (H) The protein levels of PCNA were detected by Western blot analysis in the indicated cells with different treatments as above. (I) Luciferase activities of PCNA 3′UTR were examined in HepG2.2.15 cells with indicated regiments. Error bars represent means ± SD (n=3). Statistical significant differences are indicated: *P<0.05; **P<0.01; ***P<0.001; NS, no significance; Student's *t* test.

It has been reported that the pseudogenes as sponges competing for microRNAs regulate their parental genes 24. Therefore, we explored whether pseudogene PCNAP1 modulated its ancestor PCNA by regulating microRNAs. Interestingly, we observed two miR-154 binding sites in PCNAP1 and PCNA mRNA 3′UTR by using the TargetScan (http://www.targetscan. org/) and DIANA database (http://diana.cslab.ece.ntua.gr/microT/) (Figure S8A), suggesting that PCNA mRNA 3′UTR might be one of the targets of miR-154. Clinically, TCGA data analysis revealed that miR-154 was highly elevated in HCC tissues (Figure S8B). Moreover, RT-qPCR assays revealed that the levels of miR-154 were significantly decreased in HBV-related HCC samples relative to those in their adjacent non-tumorous liver tissues (Figure 5A). We also found that the expression levels of miR-154 were negatively related with those of PCNAP1 and PCNA in 39 liver tissues of HBV-related HCC patients (Figure 5B-C), suggesting that miR-154 is negatively associated with PCNAP1/PCNA signaling. Luciferase reporter gene assays showed that overexpression of miR-154 decreased the luciferase activities of PCNA mRNA 3′UTR in a dose-dependent manner, which could be reversed by miR-154 inhibitors in 293T cell line (Figure 5D). In addition, we validated that overexpression of miR-154 decreased the luciferase activities of PCNA mRNA 3′UTR in HepG2.2.15 cells, while knockdown of miR-154 increased the luciferase activities in HepG2 cells (Figure S8C), suggesting that PCNA mRNA 3′UTR is targeted by miR-154. Furthermore, our data showed that ectopic expression of miR-154 down-regulated the levels of both RNA and protein of PCNA in HepG2.2.15 and PLC/PRF/5 cells in a dose-dependent manner (Figure 5E, Figure S8D). Conversely, the inhibitor of miR-154 led to the opposite results (Figure S8E-F), supporting that miR-154 can target PCNA in hepatoma cells.

Our data documented that PCNAP1 elevated the expression of its ancestral PCNA at post-transcriptional level (Figure 4, Figure S7). Accordingly, we hypothesized that pseudogene PCNAP1 as a endogenous sponge might compete for miR-154, leading to the up-regulation of PCNA in liver. Interestingly, luciferase reporter gene assays indicated that ectopic expression of PCNAP1 increased the luciferase activities of PCNA mRNA 3′UTR, which could be markedly blocked by miR-154 in HepG2 cells (Figure S8G). The same results were validated in HepG2.2.15 cells (Figure S8H), suggesting that pseudogene PCNAP1 enhances the expression of its ancestral PCNA through sponging miR-154 at post-transcriptional level. In addition, our data uncovered that PCNAP1 enhanced the expression of PCNA through inhibition of miR-154 in HepG2 and H7402 cells (Figure 5F-H, Figure S8I-K). Conversely, the inhibitory effect of siPCNAP1 on PCNA was reversed by miR-154 inhibitors in HepG2.2.15 and PLC/PRF/5 cells (Figure 5G-H, Figure S8J-K), suggesting that PCNAP1 competes for miR-154 in modulation of PCNA.

To further confirm that PCNAP1 could compete for miR-154 in regulation of PCNA, we cloned the 3′UTR of PCNA mRNA (termed pGL3-PCNA-WT) and its three mutants (termed pGL3-PCNA-mut 1, pGL3-PCNA-mut 2, and pGL3-PCNA-mut 1/2). The mutants were randomly designed following the principles that they could not produce another miR-154 binding site (Figure S8L). Interestingly, luciferase reporter gene assays showed that miR-154 could decrease the luciferase activities of PCNA mRNA 3′UTR in HepG2.2.15 cells. But it failed to work in the cells transfected with pGL3-PCNA-mut 1 or pGL3-PCNA-mut 1/2 (Figure 5I). Moreover, we repeated the results in HepG2 cells (Figure S8M), suggesting that the miR-154 binding site-1 in PCNA mRNA 3′UTR is the target of miR-154. Moreover, we constructed the entire region of PCNAP1 (termed p3.1-PCNAP1-WT) and its three mutants (termed as p3.1-PCNAP1-mut 1, p3.1-PCNAP1-mut 2, and p3.1-PCNAP1-mut 1/2) into pcDNA3.1 plasmids (Figure S8L). Remarkably, luciferase reporter gene assays demonstrated that miR-154 remarkably decreased the luciferase activities of PCNA mRNA 3′UTR, which could be mostly reversed by the co-transfection with p3.1-PCNAP1-WT, but the three mutants of PCNAP1 failed to work (Figure S8N), indicating that the two miR-154 binding sites in PCNAP1 are necessary for PCNAP1 modulating PCNA by competing for miR-154. In addition, we validated the results by RT-qPCR and Western blot analysis (Figure S8O-P), suggesting that PCNAP1 sponges miR-154 to stimulate PCNA expression. Thus, we conclude that PCNAP1 up-regulates the expression of PCNA by competing for miR-154.

### PCNAP1 and PCNA promote the growth of hepatoma cells *in vitro* and* in vivo*

**Figure 6:**
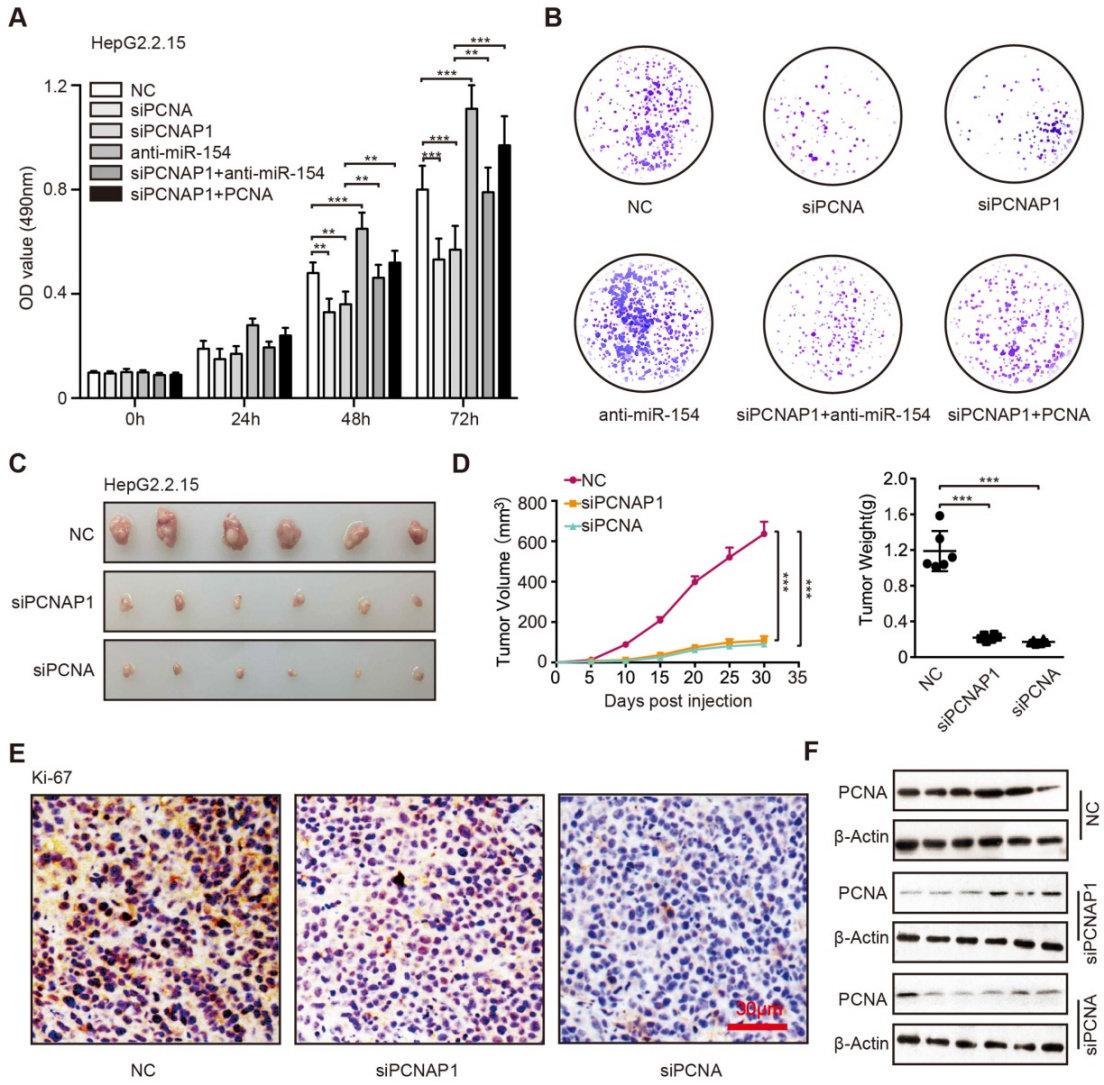
PCNAP1 and PCNA promotes the growth of hepatoma cells* in vitro* and *in vivo.* (A) The cell viability was determined by MTT assays in HepG2.2.15 cells with single treatment of siPCNA (100 nM), siPCNAP1 (100 nM) and anti-miR-154 (100 nM) or the co-transfection of siPCNAP1 and anti-miR-154 or the co-transfection of siPCNAP1 and PCNA. (B) Colony formation efficiencies were determined in HepG2.2.15 cells treated with different regiments as above. (C) Photographs of dissected tumors from nude mice injected with HepG2.2.15 cells treated with siCtr, siPCNAP1 or siPCNA. (D) The average volume and weight of the tumors transplanted with HepG2.2.15 cells pre-treated with siCtr, siPCNAP1 or siPCNA in nude mice. (E) Immunohistochemistry assays for Ki-67 were measured in tumor tissues from the nude mice treated as above. (F) The protein expression levels of PCNA were examined by Western blot analysis in the tumor tissues from nude mice, respectively. Error bars represent means ± SD (n=3). Statistical significant differences are indicated: *P<0.05; **P<0.01; ***P<0.001; Student's *t* test.

It has been reported that the HBV replication and cccDNA accumulation played critical role in the development of liver cancer, in which the interaction of host and virus was important 8. Accordingly, we concerned whether PCNAP1 could promote the proliferation of hepatoma cells through modulating the expression of miR-154 and PCNA. Surprisingly, MTT, EdU and colony formation assays revealed that siPCNA or siPCNAP1 inhibited the cell proliferation, while the co-treatment of miR-154 inhibitors or overexpression of PCNA could reverse this event (Figure 6A-B, Figure S9A-C), suggesting that pseudogene PCNAP1 promotes the proliferation of hepatoma cells through miR-154/PCNA signaling *in vitro*. Meanwhile, we observed that PCNA was able to enhance the proliferation of hepatoma cells and miR-154 had the reversed effect in the system. To better understand the role of PCNAP1 and PCNA in promotion of hepatocarcinogenesis *in vivo*, we subcutaneously injected the pretreated cells into 4-week-old BALB/c athymic nude mice. Strikingly, we demonstrated that the treatment with PCNAP1 siRNA or PCNA siRNA sharply suppressed the growth of HepG2.2.15 and HepG2 cells in mice (Figure 6C-D, Figure S9D-E). IHC staining further confirmed that the expression of Ki-67, a marker of proliferation, was markedly decreased in the tumor tissues from the nude mice transplanted with HepG2.2.15 and HepG2 cells treated with PCNAP1 siRNA or PCNA siRNA (Figure 6E, Figure S9F). Western blot analysis validated that the expression levels of PCNA were decreased in the group of mice tumor samples treated with siPCNAP1 and siPCNA (Figure 6F, Figure S9G). Thus, we conclude that PCNAP1 and PCNA significantly promotes the growth of hepatoma cells *in vitro* and *in vivo*.

## Discussion

Chronic infection of hepatitis B virus is a leading cause of liver cancer. HBV cccDNA plays a key role in viral persistence and antiviral therapy resistance. The host-virus interaction is critical for the development of HBV-related liver cancer 8. The pseudogene-derived lncRNAs serve as functional regulators of their ancestral gene expression and play important roles in multiple processes 12. However, the role of pseudogene-derived lncRNAs and their ancestral genes in modulation of HBV replication and liver cancer remains unclear. In this study, we investigated the significance of pseudogene PCNAP1 in modulation of HBV replication and hepatocarcinogenesis.

Over the last few decades, research has focused on the role of protein-coding genes in the pathogenesis of diseases. The recent advances in high-throughput transcriptome sequencing have revealed the widespread expression of pseudogenes in multiple cancer diseases 17. To address the significance of pseudogene PCNAPs in pathogenesis of diseases, we demonstrated that the levels of PCNAP1 were significantly elevated in the liver of HBV-infected human liver-chimeric mice relative to those in the livers from human liver-chimeric mice. It suggests that PCNAP1 is closely associated with HBV infection. Moreover, we identified that the pseudogene PCNAP1 was significantly elevated in 39 HBV-related HCC samples and HBV positive cell lines. It suggests that the pseudogene PCNAP1 is positively associated with HBV infection in liver. Previous study reported that a series of host molecules could modulate HBV replication and cccDNA accumulation 1. However, the pseudogene-derived lncRNAs involving modulation of HBV have been not reported. Surprisingly, in this study we found that the levels of HBV replication markers, including HBV progeny DNA, HBsAg, HBeAg, HBcAg and HBx, were modulated by PCNAP1 in HBV-infected HepaRG and HepG2.2.15 cells. It suggests that the pseudogene PCNAP1 is able to modulate HBV replication in hepatoma cells. Importantly, we found that the levels of HBV cccDNA were increased by PCNAP1 in the cells. It suggests that PCNAP1 contributes to the HBV cccDNA accumulation. Clinically, the expression levels of PCNAP1 were remarkably elevated in the HBV cccDNA-positive liver cancer tissues. Notably, we demonstrated the incidence (24/39, 61.5%) of HBV cccDNA in HBV-related HCC patients. It implies that HBV cccDNA may play crucial roles in the development of HCC. It supports that PCNAP1 is able to modulate HBV cccDNA in liver. Thus, our data first provide evidence that the host pseudogene is able to activate HBV in liver.

Pseudogene-derived lncRNAs act as functional regulators of their ancestral gene expression in multiple cellular processes 13. To better understand the mechanism by which PCNAP1 modulated HBV replication and cccDNA accumulation, we performed gene expression microarray analysis using siPCNAP1 in HepG2.2.15 cells, and found that 189 mRNAs were up-regulated and 1231 mRNAs were down-regulated. Interestingly, we found that PCNAP1 was involved in DNA repair, DNA replication, cell cycle checkpoint and p53 pathways, in which PCNA was identified as a downstream gene of PCNAP1. Basically, the pseudogenes play multiple roles through modulating their ancestral genes, such as PTEN pseudogene 1 and its ancestor PTEN 13. Consistently, we identified the ancestral gene PCNA of PCNAP1 in our system. We observed that the mRNA levels of PCNA were significantly elevated in the liver of HBV-infected human liver-chimeric mice relative to those in human liver-chimeric mice. It suggests that PCNA is closely associated with HBV infection. Next, we were interested in whether PCNA could regulate HBV replication and cccDNA accumulation. As expected, siRNA of PCNA significantly decreased the levels of HBeAg, HBsAg, HBV DNA and cccDNA in HBV-infected PHH and HepG2.2.15 cells. Moreover, overexpression of PCNA could stimulate the levels of HBV replication markers and HBV cccDNA. Then, we took advantage of the CRISPR/Cas9 system to generate PCNA knockout in HepG2 cells. The cells were transfected with PCH9/3091-HBV 1.1 plasmids. Interestingly, the levels of HBV replication markers and HBV cccDNA were remarkably decreased in the PCNA knockout cells, and the effects could be mostly rescued by ectopic expression of pcDNA3.1-PCNA. It suggests that PCNA contributes to the HBV replication and cccDNA accumulation in liver. Clinically, the expression levels of PCNA were remarkably elevated in HBV cccDNA-positive liver cancer tissues. It supports that PCNA is able to modulate HBV cccDNA in liver. PCNA, acting as a coordinator of DNA polymerase(s) in maintaining genomic integrity at both genetic and epigenetic levels, plays multiple roles in DNA replication and repair, and serves as a cellular marker for proliferation and can be used for grading of different neoplasms 14. Here, we firstly reported that PNCA plays a crucial role in modulation of HBV replication and cccDNA accumulation.

Given that pseudogenes functioned multiple roles through regulating their ancestral genes, we concerned whether the pseudogene PCNAP1 modulated HBV replication and cccDNA accumulation by its ancestor PCNA. As expected, our data demonstrated that PCNAP1 influenced the HBV replication and cccDNA accumulation through its downstream factor PCNA. Thereby, our finding is consistent with the report that the pseudogenes play multiple roles through modulating their ancestral genes. It has been reported that PCNA as a lariat anchors on DNA strains to maintain its genome integrity 22. Next, we were eager to uncover the mechanism by which PCNA promoted HBV replication and cccDNA accumulation. Strikingly, we demonstrated that PCNA could bind to cccDNA in HepaRG and HepAD38 cells. It has been reported that HBc as a guider protein initiates the cccDNA formation and HBV viral transcription 23. Accordingly, we validated that PCNA was able to interact with HBc and modulated HBV replication and cccDNA accumulation in a HBc-dependent manner.

Upon infection, HBV cccDNA is generated as a plasmid-like episome in the host cell nucleus from the protein-linked rcDNA genome 1. In the event, its fundamental role is serving as template for all viral RNAs, leading to new virions. Biosynthesis of rcDNA by reverse transcription of the viral pregenomic RNA is now understood in considerable detail, yet conversion of rcDNA to cccDNA is still obscure, due to the lack of feasible cccDNA-dependent assay systems. Conceptual and recent experimental data link cccDNA formation to cellular DNA repair, which is increasingly appreciated as a critical interface between cells and viruses. However, the details of the formation, stability and epigenetic regulation of cccDNA remains unclear. It has been reported that PCNA is involved in the DNA repair pathway 22. Therefore, we speculated that PCNA might play key role of cccDNA formation by regulating DNA repair. However, the mechanism by which PCNA modulates HBV replication and cccDNA accumulation in detail will be further investigated.

Previous study reported that the pseudogene modulated their ancestral genes at post-transcriptional level 17. Consistently, our data validated that PCNAP1 regulated its ancestral PCNA at post-transcriptional level. It has been reported that the pseudogenes as sponges competing for microRNAs regulated their parental genes 24. Accordingly, we predicted miR-154 binding sites in PCNAP1 and PCNA mRNA 3′UTR using the TargetScan and DIANA database. The expression of miR-154 is down-regulated in several types of cancers, including hepatocellular carcinoma, colorectal cancer, non-small cell lung cancer, breast cancer, and prostate cancer. MiR-154 acts as a tumor suppressor to inhibit the cell proliferation and metastasis 18. Interestingly, we found that the levels of miR-154 were significantly decreased in HBV-related HCC samples relative to those in their adjacent non-tumorous liver tissues. We also observed that the expression levels of miR-154 were negatively associated with those of PCNAP1 or PCNA in 39 clinical tumorous liver tissues. It suggests that miR-154 as a potential tumor suppressor is involved in modulation of PCNAP1/PCNA signaling. As expected, we found that PCNAP1 sponged miR-154 to stimulate PCNA expression in hepatoma cells. It suggests that PCNAP1 up-regulates the expression of PCNA by competing for the tumor-suppressive miR-154.

As we all know, HBV is a major risk factor for liver cancer, in which HBV cccDNA plays crucial roles 1. Thereby, we concerned whether PCNAP1/PCNA signaling, as a regulating factor of HBV replication and cccDNA accumulation, could promote the proliferation of hepatoma cells. Our data showed that PCNAP1/PCNA signaling significantly promoted the growth of hepatoma cells *in vitro* and *in vivo*. It suggests that PCNAP1/PCNA signaling can enhance the growth of both HBV-related HCC and HBV-free HCC.

Taken together, we conclude that the host lncRNA PCNAP1 enhances HBV replication through modulating miR-154/PCNA/HBV cccDNA signaling and the PCNAP1/PCNA signaling drives the hepatocarcinogenesis, implying that the elevated HBV by PCNAP1 may accelerate the hepatocarcinogenesis because the chronic infection of HBV is a leading cause of liver cancer. Thus, our finding provides new insights into the mechanism by which PCNAP1 modulates HBV replication and hepatocarcinogenesis.

In summary, in this study we firstly report that the lncRNA PCNAP1 promotes HBV replication and hepatocarcinogenesis (Figure S10). In the model, PCNAP1 as a natural sponge of miR-154 regulates its ancestral PCNA, leading to the HBV replication and cccDNA accumulation. HBc-recruited PCNA binds to HBV cccDNA. Functionally, the PCNAP1/PCNA signaling contributes to the HBV replication and hepatocarcinogenesis.

## Materials and Methods

### Generation of human liver-chimeric mice

The human liver-chimeric mice were generated by VITALSTAR (Beijing, China). Primary human hepatocytes (PHHs) were transplanted into 3-week-old urokinase-type plasminogen activator/severe combined immunodeficient beige (uPA/SCID-bg) mice (male and female) by intrasplenic injection as described 3, 25. Engraftment and viability of PHHs were assessed by quantification of human serum albumin by enzyme-linked immunosorbent assay (Human Albumin ELISA kit, Immunology Consultants Lab, Portland, USA). Then, the uPA/SCID-bg mice were infected with 2.5E+08 IU/ml (0.2ml/mouse) HBV particles from the supernatant of HepAD38 cells (tet-off) and sacrificed 8 weeks after virus inoculation. Serum HBV load in the mice was determined by quantitative PCR (Da An Gene, Guangzhou, China) before sacrifice. The information of human liver-chimeric mice was shown in Table S1. The expression levels of PCNAP1 and PCNA were analysis in liver tissues of the mice. The Institute Research Ethics Committee at the Nankai University approved the study protocol.

### Cell culture and generation of stable cell lines

Hepatoma cell lines HepG2 and HepG2.2.15 cells were maintained in Dulbecco's modified Eagle's medium (Gibco, Grand Island, NY). HepaRG and H7402 cell lines were cultured in RPMI Medium 1640 (Gibco) supplemented with 10% FBS. The HepAD38 cell line regulates HBV replication through the presence or absence of tetracycline in the culture medium. HepAD38 cell was cultured in DMEM/F12 medium (Life Technologies, Carlsbad, CA) supplemented with 10% heat-inactivated FBS (Fetal Bovine Serum, FBS), 100 U/ml penicillin, 100 μg/ml streptomycin, 100 μg/ml kanamycin, 400 μg/ml G418, and with 0.3 μg/ml tetracycline (for inhibition of HBV replication) or without any tetracycline (for induction of HBV replication). PHHs were purchased from Shanghai RILD Inc. (Shanghai, China). The cells were cultured similarly using the same plating and incubation medium as described above 26, 27. All the cell lines were treated with 100 U/ml penicillin, and 100 mg/ml streptomycin in 5% CO_2_ at 37°C. The cells were cultured in a 6-well, 24-well or 96-well plate for 12 h and then were transfected with plasmids or siRNAs. The transfections were performed using Lipofectamine 2000 reagent (Invitrogen, Carlsbad, CA, USA) or lipofectamine Messenger Max^TM^ Reagent for PHH (Invitrogen, Carlsbad, CA, USA) according to the manufacturer's protocol.

### Patient samples

Forty-three liver tissues from HCC patients utilized in this study were immediately obtained from Tianjin First Center Hospital (Tianjin, P.R. China) and Tianjin Medical University Cancer Institute and Hospital (Tianjin, P.R. China) after surgical resection. Clinicopathological information about the patients was obtained from patient records, and was summarized in Table S2. Written consents approving the use of their tissues for research purposes after operation were obtained from patients. The Institute Research Ethics Committee at the Nankai University approved the study protocol.

### Model of HBV transgenic BALB/c mice (HBV-Tg)

HBV-Tg containing the intact HBV genome were obtained from VITALRIVER experiment animal company (Beijing, China). The generation and specific histological changes of HBV-Tg mice have been reported by our and other laboratory 7, 8, 28.

### PCNA pseudogenes analysis and sequencing

Accordingly 29, genome blast analysis (http://blast.ncbi.nlm.nih.gov/) was performed to identify PCNA pseudogenes. Genomic coordinates of identified pseudogenes were obtained using the University of California Santa Cruz (UCSC) genome browser. All candidate pseudogenes were PCR amplified from HepG2.2.15 cells cDNA using specific primers (Table S3). PCR amplicons were cloned into pcDNA3.1+ vector (Life Technologies) and subjected to bi-directional sequencing.

### Plasmid constructions

The 5'-flanking region (nucleotides -1500/+200) of PCNA was amplified by PCR from the genomic DNA of HepG2 using specific primers and was cloned into the upstream of the pGL3-basic vector (Promega, Madison, WI, USA) *via* KpnI and XhoI sites. The resulting plasmid was sequenced and named pGL3-PCNA-promoter. The 3' untranslated region of PCNA was amplified by PCR from the cDNA pool of HepG2 using specific primers and was cloned into the downstream of the pGL3-control vector (Promega, Madison, WI, USA) *via* FseI and XbaI sites. The resulting plasmid was sequenced and named pGL3-PCNA-3'UTR. Mutant constructs of pGL3-PCNA-3'UTR were named as pGL3-PCNA-mut1 and pGL3-PCNA-mut2, which carried the substitution of core nucleotides shown in the Table S3. The CDS region of PCNA was amplified by PCR from the cDNA of HepG2.2.15 cells using specific primers and was cloned into the pcDNA3.1, pcDNA4.0, pmCherry-C1 and pCMV-Tag2B vector. Especially, the wild type and mutants of PCNA were cloned into pCMV-Tag2B vector, including pCMV-Tag2B-PCNA-wt (termed as WT), pCMV-Tag2B-PCNA-DNA domain mutants (termed as DM), pCMV-Tag2B-PCNA-protein domain mutants (termed as PM). The whole region of PCNA pseudogenes and PCNAP1 mutants with the mutation of miR-154 binding sites were amplified by PCR from the cDNA of HepG2.2.15 cells using specific primers and were cloned into the pcDNA3.1 vector. The CDS region of HBc and its serial fragments were amplified by PCR from the cDNA of HepG2.2.15 cells using specific primers and were cloned into the pcDNA4.0 and pEGFP-n1 vector, including pcDNA4.0-HBc-wt (termed as HBc or His-HBc), pcDNA4.0-HBc 1-44aa (termed as His-HBc-44aa), pcDNA4.0-HBc 45-183aa (termed as His-HBc-45aa), pcDNA4.0-HBc 1-78aa (termed as His-HBc-78aa), pcDNA4.0-HBc 79-183aa (termed as His-HBc-79aa). All primers are listed in Table S3.

### RNA extraction, reverse-transcription polymerase chain reaction (RT-PCR), and quantitative real-time PCR (RT-qPCR)

Total RNA was extracted from cells (or liver tissues from HBV-Tg mice, HBx-Tg mice and patient tissues) using Trizol reagent (Invitrogen, Carlsbad, CA, USA). First-strand cDNA was synthesized as reported before 9, 30. RT-qPCR was performed by a Bio-Rad sequence detection system according to the manufacturer's instructions using double-stranded DNA-specific SYBR GreenPremix Ex TaqTM II Kit (TaKaRa, Ohtsu, Japan). Experiments were conducted in three independent assays. Relative transcriptional folds were calculated as 2^-∆∆Ct^. GAPDH was used as an internal control for normalization. All the primers used are listed in Table S3.

### Western blot analysis

Total protein lysates were extracted from hepatoma cells with RIPA buffer. Protein concentrations were measured using the BCA protein Quantification Kit (YEASEN, China), and 20-50 μg protein extracts were subjected to SDS-PAGE 31, 32. Then proteins were transferred to a PVDF membrane, blocked with 5% non-fat milk and incubated with first antibodies for 1h at R.T. After incubation with secondary antibody against mouse (1:10,000) or rabbit (1: 10,000) for 1h at 37°C, the membrane was visualized by ECL Western Blotting Detection Kit (GE Healthcare, Waukesha, WI, USA). The primary antibodies used for Western blot analysis were listed in Table S4.

### Nuclear and cytoplasm RNA extraction

Accordingly 29, 33, cells were collected, resuspended in lysis buffer (10 mM NaCl, 20 mM MgCl, 10 mM Tris-HCl, pH 7.8, 5 mM DTT, 0.5% NP-40) and kept in ice for 5 minutes. Nuclei were pelleted by centrifugation at 8000 rpm for 5 minutes at 4°C, pellets were washed and resuspended in lysis buffer. The cytoplasmic fraction was collected to a new tube and clarified by centrifugation. Nuclear and cytoplasmic fractions were subjected to protease treatment for 20 minutes at 37°C by adding an equal volume of proteinase K solution (300 mM NaCl, 0.2 M Tris-HCl, pH7.5, 25 mM EDTA, 2% SDS and 0.1 mg/ml proteinase K), and RNA was purified using the QIAzol Lysis Reagent (Qiagen, Germany). The RNA was subjected to DNase treatment; cDNA was synthesized using the QuantiTect Reverse Transcription Kit (Qiagen, Germany) according to the manufacture's protocol. Quantitative real time PCR was performed on StepOnePlus real-time PCR machine (Applied Biosystems, UK), using SYBR Green Universal PCR Master Mix (Applied Biosystems, UK). Oligonucleotides used for quantitative and semi-quantitative PCR are listed in Table S3.

### HBV preparation and infection

HBV used in this study was mainly derived from HepAD38 cells, which is classified as genotype D. HepAD38 cells were cultured in DMEM/F12 medium (Life Technologies, Carlsbad, CA, USA) supplemented with 10% heat-inactivated FBS, 100 U/ml penicillin, 100 μg/ml streptomycin, 100 μg/ml kanamycin, 400 μg/ml G418, and with 0.3 μg/ml tetracycline (for inhibition of HBV replication) or without any tetracycline (for induction of HBV replication, termed as HepAD38*). Media were recovered every 3 days from HepAD38* cells at day's 7-15 post-induction of HBV by depletion of tetracycline. Media were cleared through a 0.45 μm filter and precipitated with 10% PEG8000 and 2.3% NaCl. The precipitates were washed and resuspended with medium at 200-fold concentration. The HBV DNA was quantified by qPCR. HepaRG and PHH cells were infected with HBV at 500-1000 genome equivalents (GEq)/cell in the presence of 4% PEG8000 at 37˚C for 16 h as previously described 27, 34. Under these conditions, efficient infection of HepaRG cells (termed as HepaRG*) using virus derived from HepAD38 cells requires an inoculum of >10^4^ HBV GEq/cell 27. HepG2 cells were transfected with PCH9/3091 expressing the whole HBV genome to induce the HBV life cycle (termed as HepG2*).

### HBV cccDNA isolation

The experiments were carried out in the cells as described with minor modifications 35. The cells were lysed in lysis buffer A (50 mM Tris-HCl pH 7.4, 1 mM EDTA, 1% NP-40) containing complete protease inhibitor cocktail for 30 min on ice. After centrifugation, the pelleted nuclei were resuspended in lysis buffer B (10 mM Tris-HCl, 10 mM EDTA, 150 mM NaCl, 0.5% SDS, Proteinase K 0.5 mg/ml) and incubated overnight at 37°C. Nucleic acids were purified by phenol-chloroform (1:1) extraction and ethanol precipitation.

### Southern blot analysis of HBV cccDNA

Selective extraction of HBV cccDNA from HBV infected cells was achieved as previously described 19, 26, 35. For detection of cccDNA by Southern blot analysis, the extracted HBV cccDNA sample was subjected to 1.2% agarose gel electrophoresis and transferred onto Amersham Hybond-N+ membrane (GE Healthcare, USA). The Hybond-N+ membrane was cross-linked in a UV cross linker chamber with UV energy dosage at 1500 mJ and followed by being probed with “DIG-labeled probes of linear HBx DNA fragments” for 24 h, 37°C. And the membrane was blocked and incubated with Anti-Digoxigenin-AP (dilute Anti-Digoxigenin-AP 1:10 000 (75 mU/ml) in blocking solution) for overnight at 4 °C. After washing for 15 min, we placed the membrane with DNA side facing up on a development folder (or hybridization bag) and apply 1 ml CSPD ready-to-use (bottle 5). Immediately cover the membrane with the second sheet of the folder to spread the substrate evenly and without air bubbles over the membrane. Incubate for 5 min at 15-25°C. When indicated, isolated DNA was digested with EcoRI that linearizes the HBV cccDNA.

### HBV DNA specific dot blot for progeny HBV secretion

Viral DNA in the supernatants was extracted following the method described as before 19. The concentration was measured by NanoDrop. In order to increase sensitivity and accuracy, we amplified the specific fragments using the HBx primers and obtained the products by “Gel Extraction purification”. Isolated products were first denatured by heating at 95°C for 3 min, followed by chilling on ice directly. Next, the DNAs were spotted onto Amersham Hybond-N+ membrane (GE Healthcare, USA) and the nucleic acids were UV cross-linked in an Ultraviolet Crosslinker (Ultra-Violet Products, Cambridge, UK). Then, the membrane was washed by PBST buffer (PBS Tween), blocked with 5% of BSA in PBST and hybridized with “DIG-labeled probes, which were used for hybridization to membrane blotted nucleic acids according to standard methods” 19. The sensitivity of this technique, determined by using serial dilutions of HBV DNA (isolated from PCH9/3091-HBV plasmid) of known concentration, was less than 1 pg of cloned DNA. And the membrane was incubated with Anti-Digoxigenin-AP (dilute Anti-Digoxigenin-AP 1:10000 (75 mU/ml) in blocking solution) for overnight at 4 °C. After washing for 15 min, we placed the membrane with DNA side facing up on a development folder (or hybridization bag) and apply 1 ml CSPD ready-to-use (bottle 5), and immediately covered the membrane with the second sheet of the folder to spread the substrate evenly and without air bubbles over the membrane, and incubated for 5 min at 15-25°C.

### Immunofluorescence assays

Virus infected cells in 48-well plates were washed three times with pre-cooled PBS and fixed by 4% paraformaldehyde for 10 min, followed by permeabilization for 10 min at room-temperature with 0.5% Triton X-100. After incubation for 1 h with 3% BSA for blockade of nonspecific binding, primary antibodies were added for incubation for 1 h at 37°C. The bound antibodies were visualized by incubation with secondary antibodies. Images were acquired using a Nikon A1-R confocal microscope or a Nikon Eclipse Ti Fluorescence Microscopy 4.

### HBV cccDNA-ChIP assays

HBV cccDNA-ChIP experiments were carried out in hepatoma cells post transfection as described with minor modifications 35. Briefly, the cells were fixed with 1% formaldehyde for 10 min at 37 ^o^C and quenched with 0.125 M Glycine. For nuclear extracts preparation, the cells were lysed in buffer A (0.25% Triton X-100, 10 mM Tris pH8, 0.5 mM pefablock, EDTA-free protease inhibitors, Roche, USA). After centrifugation, nuclei were washed in buffer B (0.2 M NaCl, 10 mM Tris pH8, 0.5 mM pefablock, EDTA-free protease inhibitors), centrifuged and lysed in nuclei lysis buffer (1% SDS, 10 mM EDTA, 50 mM Tris pH8, 0.5 mM pefablock, EDTA-free protease inhibitors). After sonication, the lysates were diluted 1:10 with 0.01% SDS, 1% Triton X-100, 1.2 mM EDTA, 16.7 mM Tris pH8, 167 mM NaCl 0.5 mM pefablock, EDTA-free protease inhibitors. Chromatin was then subjected to overnight immunoprecipitation at 4^o^C using 2-5 g of antibodies listed in Table S4. Negative controls with IgG were included in each experiment. Immune complexes were incubated with protein A/G agarose beads at 4^o^C, washed, and eluted in 1% SDS, 0.1% NaHCO_3_. Immunoprecipitated DNA was quantified by qPCR using cccDNA specific primers (Table S3). Samples were normalized to input DNA using the DCt method were DCt = Ct (input) △Ct (immunoprecipitation) and calculated as percentage of the input. Results are expressed as the average of at least three independent experiments. Error bars represent means ± SD (n=3). Statistical differences were analyzed by Student's *t* test.

### Microarray analysis

HepG2.2.15 cells were treated with 100 nM PCNAP1 siRNA or control siRNA. Then, RNA was extracted with Trizol (Invitrogen, Carlsbad, CA, USA) and verified with RNA integrity number (RIN). The amino allyl-RNA (aRNA) probes labeled with NHS-Cy5 (Amersham, Sweden) were hybridized at 50°C for 16 hours to the Human Whole Genome OneArray TM Version 4.3 (Phalanx Biotech Group, Taiwan, PR China), scanned with Axon 4000B Scanner (Molecular Devices, USA) and analyzed with Genepix software (Molecular Devices USA). Further data were analyzed according to the manufacturer's protocol 8. The results were shown in Table S5.

### Hepatitis B surface antigen (HBsAg) and Hepatitis B e antigen (HBeAg) quantification

Secretion of HBsAg into the supernatants of cultured cells was measured by diagnostic kit for hepatitis B virus surface antigen according to the manufacturer's instructions (Kehua Bio-engineering, Shanghai, China). The cut-off value (COV) for HBsAg analysis was indicated as: COV =OD (negative control)/0.100. HBeAg in the supernatants of cultured cells was measured by diagnostic kit for Hepatitis B e antigen according to the manufacturer's instructions (Kehua Bio-engineering, Shanghai, China). The cut-off value for HBeAg analysis was indicated as: COV= OD (negative control)*2.1 (0<OD_NC_≤0.050, COV=0.050*2.1=0.105; 0.050<OD_NC_≤0.100; OD_NC_>0.100, invalidation) 26.

### HBV DNA analysis

 The HBV DNA in the supernatants of HepAD38 cells, HepG2.2.15 cells, HepG2 cells transfected with pCH-9/3091, and primary human hepatocytes (PHH) and HepaRG cells infected with different dose of HBV particles were extracted using the Blood & Cell Culture DNA kit (QIAGEN, Germany) following the manufacturer's instructions. The qPCR was used to quantify HBV DNA copies according to a diagnostic kit for quantification of HBV DNA (Da An Gene, Guangzhou, China) in a Bio-Rad sequence detection system 8.

### HBV cccDNA quantification

Before lysis, cell number was counted using Cell Counting Chamber Set (Qiujing, Shanghai, China). The procedure of the isolation of HBV cccDNA from the cells has been reported. Quantification of HBV cccDNA was performed by using the method described with minor modifications 19. Briefly, aliquots of each DNA extracted from cell pellets were treated for 1 hour at 37°C with 10 U Plasmid-safe ATP-dependent DNase (Epicentre, Madison, WI, USA). qPCR experiments were performed in a Mastercycler ep realplex (Eppendorf, Germany) using a 20 μL reaction volume containing 2 μl digested HBV DNA. Primers, which were used to amplify the cccDNA, were listed in Table S3. The efficacy of DNase treatment in the elimination of OC and SS forms of HBV DNA prior to PCR was confirmed by the abrogation of the PCR amplification of HBV DNA extracted from cytoplasmic viral particles by the nonselective HBV oligonucleotide primers HBV 18 (sense) and HBV 2 (antisense), which target the HBs ORF 36. Serial dilutions of a plasmid containing a monomeric genotype D HBV insert were used as quantification standards.

### Gene knockout of PCNA in HepG2 cells

Multiple sgRNAs for PCNA DNA target were screened from the BlueHeron (https://wwws.blueheronbio.com/external/tools/gRNASrc.jsp) and https://benchling.com. The sgRNAs was designed based on the target site sequence (20bp) and needs to be flanked on the 3' end by a 3bp NGG PAM sequence. DNA oligonucleotides, which harbored variable 20-nt sequences for Cas9 targeting, were annealed to generate short double-strand DNA and inserted into BsmBI-digested plasmid LentiCRISPR (pXPR-001) according to previous study 20, 21. Primers related to the sgRNAs were listed in Table S3. Then, Cas9/sgRNA co-expression constructs based upon pXPR-001 were co-transfected into HepG2 cells. Following the limiting dilution of genetically manipulated cells to screen for monoclonal mutant cells, the clones harboring indel mutations giving rise to PCR product length polymorphisms were sequenced for validation of frame shift. Primers were listed in Table S3. To assess the efficiency generated by Cas9/sgRNA, the cell lysate was subjected to Western blot analysis.

### Modeling of HBc/ PCNA interaction

A three-dimensional structure of HBV core protein was generated by automatic modeling mode of SWISS-MODEL server (swissmodel.expasy.org) 3, 37. The crystal structure of the human hepatitis B virus capsid (PDB code 1QGT, chains D) 38 was adapted as a template to perform the homology-modeling. In silico docking of PCNA (PDB code 1AXC) 39 and modeled HBV core was carried out by using Hex protein docking server (http://hexserver.loria.fr/) 40. The result was displayed and analyzed by Rasmol 2.7.5.

### Luciferase reporter gene assays

Luciferase reporter gene assays were performed using the Dual-Luciferase Reporter Assay System (Promega, Madison, WI, USA) according to the manufacturer's instructions 7, 8, 41. Cells were transferred into 24-well plates at 3× 10^4^ cells per well. After 24 h, the cells were transiently co-transfected with 0.1 μg/well of pRL-TK plasmid (Promega, Madison, WI, USA) containing the Renilla luciferase gene used for internal normalization, plasmids containing the PCNA promoter region and pGL3-basic as negative control or plasmids containing PCNA 3′UTR and PGL3-control as negative control. The luciferase activities were measured as previously described 42. All experiments were performed at least three times.

### Oligonucleotides

The siRNAs of HBc 43, PCNA 44, PCNAP1 and control siRNA (siCtrl) were purchased from RiboBio (Guangzhou, China). All oligonucleotide sequences are listed in Table S6.

### Statistical analysis

Each experiment was repeated at least three times. Statistical significance was assessed by comparing mean values (± SD) using a Student's *t* test for independent groups and was assumed for *P<0.05; **P<0.01; ***P<0.001. One-way analysis of variance was performed to compare PCNAP1/PCNA expression in all individual HBV negative hepatoma cell lines with HBV-related hepatoma cell lines. Pearson's correlation coefficient was used to determine the correlation between PCNAP1/PCNA, miR-154 and HBx mRNA levels in tumorous tissues. PCNAP1, PCNA and miR-154 expression in tumor tissues and matched adjacent non-tumor tissues were compared using the Wilcoxon signed-rank test.

## Supplementary Material

Supplementary materials and methods, figures and tables.

## Abbreviations

HBV: Hepatitis B virus
cccDNA: covalently closed circular DNA
rcDNA: relaxed circular DNA
lncRNAs: long noncoding RNAs
HCC: hepatocellular carcinoma
PHH: primary human hepatocytes
HBx: hepatitis B virus X protein
miRNAs: microRNAs
RT-PCR: reverse-transcription polymerase chain reaction
RT-qPCR: quantitative real-time PCR
ChIP: chromatin Immunoprecipitation
HBsAg: Hepatitis B surface antigen
HBeAg: Hepatitis B e antigen
