# Supplementary material for: LncRNA PCNAP1 modulates hepatitis B virus replication and enhances tumor growth of liver cancer
Source: Theranostics. 2019 Jul 9;9(18):5227–45. doi: 10.7150/thno.34273 (PMC6691589; doi:10.7150/thno.34273)
Supplement: Supplementary file 1 — Supplementary materials and methods, figures and tables. [file thnov09p5227s1.pdf]

Supplementary materials and methods

Analysis of tumorigenicity in nude mice.

All experimental procedures involving animals were in accordance with the Guide for the Care and Use of Laboratory Animals (NIH publications nos. 80-23, revised 1996) and were performed according to the institutional ethical guidelines for animal experiment [1-3]. HepG2.2.15 and HepG2 cells were transfected *in vitro* with 100 nM (final concentration) siCtrl or siPCNAP1 by using Lipofectamine RNAiMAX (Invitrogen, Carlsbad, CA). At 48 hours after transfection, 10^7 viable cells were injected subcutaneously into the right flanks of 4- to 6-week-old male BALB/c athymic nude mice, six mice per group. Tumor growth was measured after 10 days from injection and then every 5 days. At 30 days after injection, mice were sacrificed and tumors were weighed after necropsy. Tumor volume (V) was monitored by measuring the length (L) and width (W) with calipers and calculated with the formula [4] * 0.5. The Institute Research Ethics Committee at the Nankai University approved the study protocol.

Analysis of cell proliferation.

The protocol was described previously [1, 5-8]. Hepatoma cells were seeded into 96 well plates (2000 cells/well) for 12 hours before transfection and 3-(4,5-dimethylthiazol-2-yl)-2,5-diphenyltetrazolium bromide (MTT) assay was used to assess cell proliferation every day from the first day until the third day after transfection. 5-ethynyl-2'-deoxyuridine [9] incorporation assay was carried out using the Cell-Light™ EdU imaging detecting kit according to the manufacturer's instructions (RiboBio, Guangzhou, China). For colony formation analysis, 48 hours after transfection, 1000 viable transfected cells (HepG2.2.15,

HepG2 and HepG2-X cells) were placed in 6-well plates and maintained in complete medium for 2 weeks. Colonies were fixed with methanol and stained with methylene blue.

Co-immunoprecipitation assays.

The co-immunoprecipitation (co-IP) protocol was described in detail in previously published articles [10]. The lysates harvested from Hepatoma cells transfected with different regiments as the manufacturer's instructions were then subjected to overnight immunoprecipitation at 4°C using 2–5 g of antibodies listed in Table S4. Negative controls with IgG were included in each experiment. Immune complexes were incubated with protein A/G agarose beads at 4°C. The precipitates were washed six times with ice-cold lysis buffer, resuspended in the PBS, and resolved by SDS-PAGE followed by Western blot analysis.

Immunohistochemistry.

The tumor tissue from nude mice were fixed and embedded with paraffin after those mice were sacrificed. Immunohistochemical staining of samples were performed as previously reported [1, 6-8] and the primary antibody of rabbit anti-Ki-67 was used (Table S4).

TCGA database

Public TCGA (https://portal.gdc.cancer.gov/) data repositories for liver hepatocellular carcinoma (LIHC) (Cancer Genome Atlas Network, 2014) were used to analyze the expression of PCNA and miR-154.

References

1. Gao Y, Feng J, Yang G, Zhang S, Liu Y, Bu Y, et al. HBx-elevated MSL2 modulates HBV cccDNA through inducing degradation of APOBEC3B to enhance hepatocarcinogenesis. Hepatology. 2017; 66: 1413-1429.

2. Wang Y, Chen F, Zhao M, Yang Z, Li J, Zhang S, et al. The long noncoding RNA HULC promotes liver cancer by increasing the expression of the HMGA2 oncogene via sequestration of the microRNA-186. The Journal of biological chemistry. 2017; 292: 15395-407.

3. Wang Y, Chen F, Yang Z, Zhao M, Zhang S, Gao Y, et al. The fragment HMGA2-sh-3p20 from HMGA2 mRNA 3'UTR promotes the growth of hepatoma cells by upregulating HMGA2. Scientific reports. 2017; 7: 2070.

4. Verrier ER, Yim SA, Heydmann L, El Saghire H, Bach C, Turon-Lagot V, et al. Hepatitis B virus evasion from cyclic guanosine monophosphate-adenosine monophosphate synthase sensing in human hepatocytes. Hepatology. 2018; 68: 1695-709.

5. Shan C, Xu F, Zhang S, You J, You X, Qiu L, et al. Hepatitis B virus X protein promotes liver cell proliferation via a positive cascade loop involving arachidonic acid metabolism and p-ERK1/2. Cell research. 2010; 20: 563-75.

6. Cui M, Xiao Z, Wang Y, Zheng M, Song T, Cai X, et al. Long noncoding RNA HULC modulates abnormal lipid metabolism in hepatoma cells through an miR-9-mediated RXRA signaling pathway. Cancer Res. 2015; 75: 846-57.

7. Zhang T, Zhang J, You X, Liu Q, Du Y, Gao Y, et al. Hepatitis B virus X protein modulates oncogene Yes-associated protein by CREB to promote growth of hepatoma cells. Hepatology. 2012; 56: 2051-9.

8. Feng GX, Li J, Yang Z, Zhang SQ, Liu YX, Zhang WY, et al. Hepatitis B virus X protein promotes the development of liver fibrosis and hepatoma through downregulation of miR-30e targeting P4HA2 mRNA. Oncogene. 2017; 36: 6895-905.

9. Yueduan W, Jinhua Z, Changling D, Yuhong Z, Lifang W, Bing S, et al. [Significance of the expression of proliferating cell nuclear antigen and P53 in the regeneration process of an atrophic parotid gland]. Hua Xi Kou Qiang Yi Xue Za Zhi. 2017; 35: 583-7.

10. Lee HW, Kyung T, Yoo J, Kim T, Chung C, Ryu JY, et al. Real-time single-molecule co-immunoprecipitation analyses reveal cancer-specific Ras signalling dynamics. Nat Commun. 2013; 4: 1505.

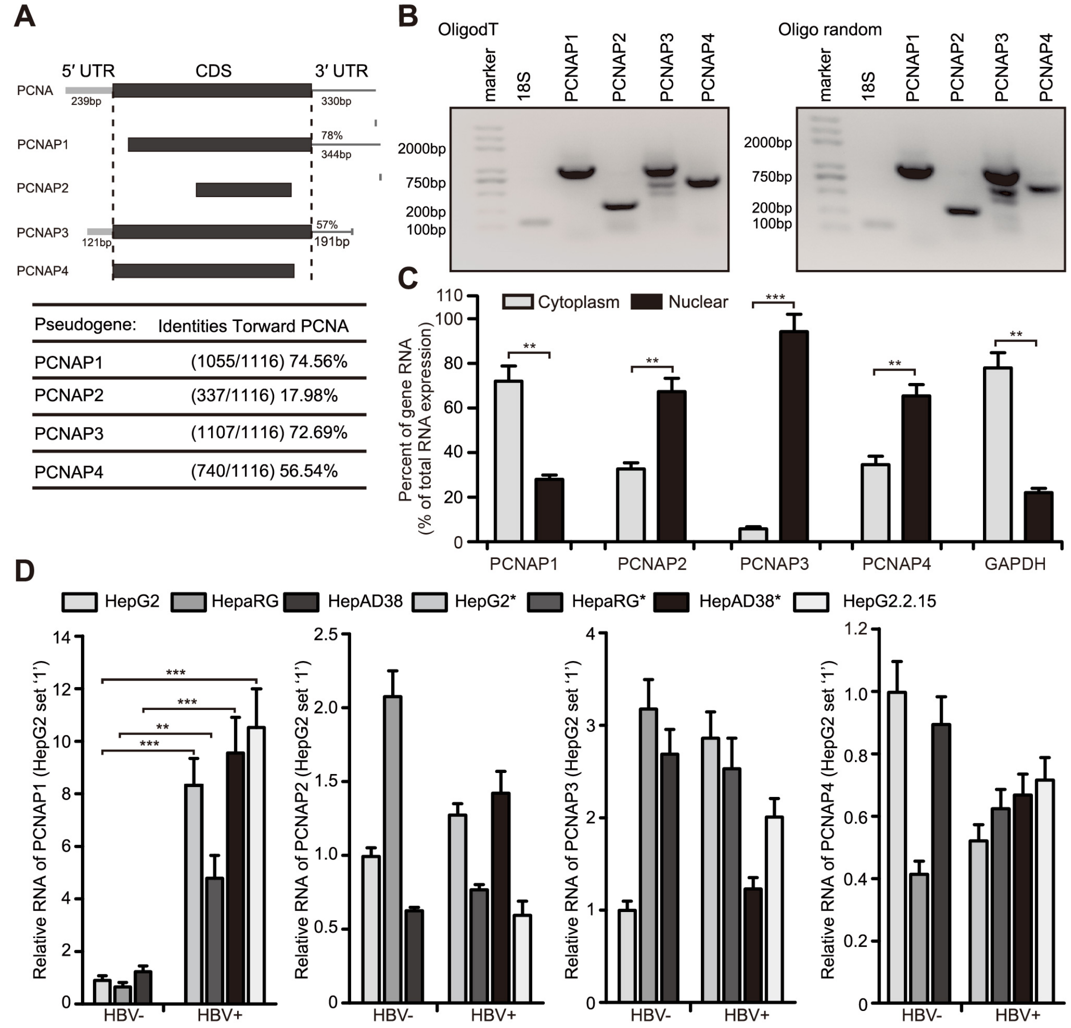

Figure S1. Characterization of human PCNA pseudogene PCNAP1. (A) Schematic representation of PCNA and PCNA pseudogenes transcripts. Regions with high homology to the PCNA mRNA 5′UTR or 3′UTR are indicated. (B) Verification of PCNA pseudogene expression in HepG2.2.15 by RT-PCR using oligo-dT primed (left panel) or random primed (right panel) cDNA. (C) Subcellular localization of PCNA pseudogene lncRNA in HepG2.2.15 cells, and the gene RNA of GAPDH were tested as a positive control. Expression values are shown as percentage of total RNA expression. (D) PCNA pseudogenes expression in HBV free or HBV positive hepatoma cells. Error bars represent means ± SD (n=3). Statistical significant differences are indicated: ***P*<0.01; ****P*<0.001; NS, no significance;

Student's *t* test.

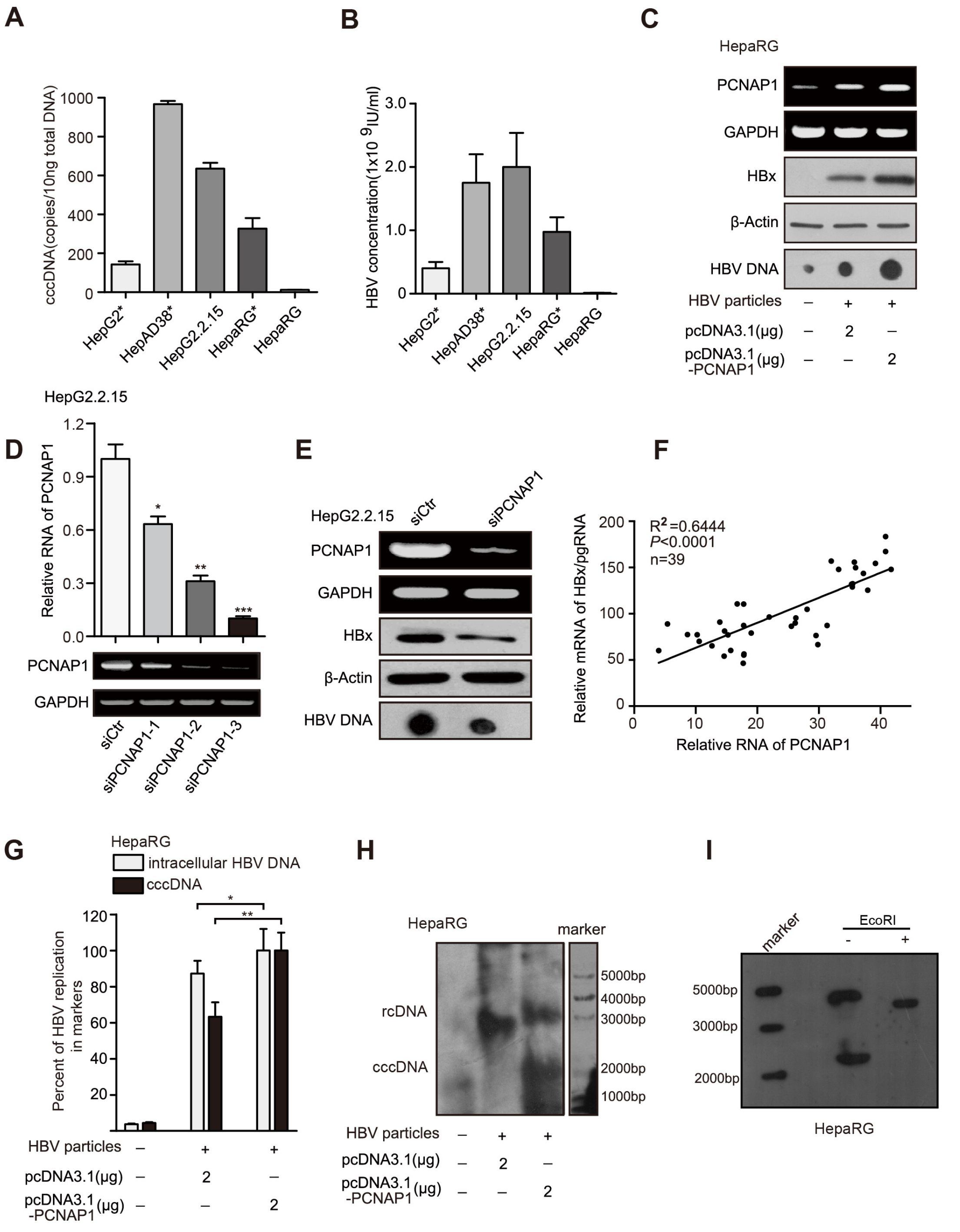

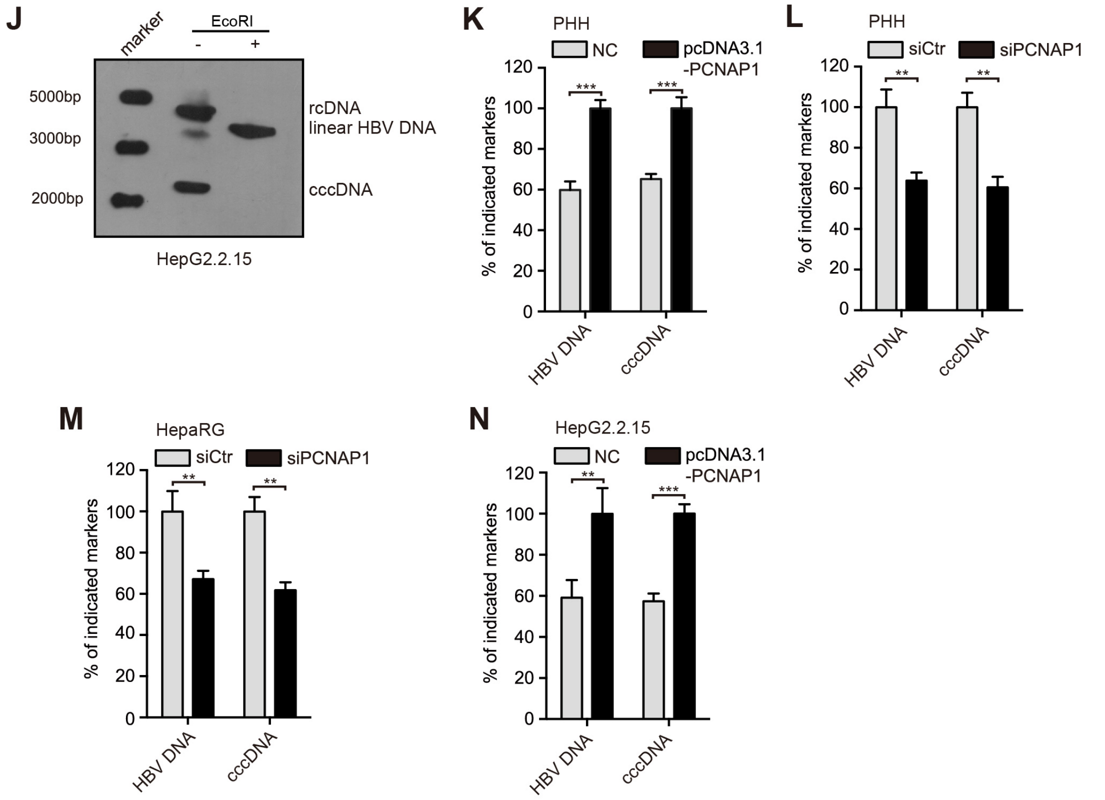

Figure S2. Pseudogene PCNAP1 promotes HBV replication and cccDNA accumulation. (A and B) The levels of HBV cccDNA and HBV progeny concentration were measured by qPCR in HBV-expressing cell lines including HepG2*/HepG2.2.15/HepAD38*/HepaRG* as well as HepaRG cells without HBV infection as negative control. (C) The levels of PCNAP1, HBx and HBV progeny DNA were detected by RT-PCR, Western blot analysis and dot blot assays in HepaRG and HBV-infected HepaRG cells treated with pcDNA3.1 or pcDNA3.1-PCNAP1. (D) The silencing efficiency of siPCNAP1s was examined by RT-qPCR and RT-PCR in HepG2.2.15 cells. (E) In HepG2.2.15 cells treated with siCtr or siPCNAP1, the expression of PCNAP1, HBx and HBV progeny DNA were tested by RT-PCR, Western blot analysis and dot blot assays, respectively. (F) Correlation between the PCNAP1 RNA levels and the HBx levels was examined by RT-qPCR in 39 cases of HCC tissues (***P<0.0001, R^2=0.6444, Pearson's correlation coefficient). (G and H) The intracellular HBV DNA and cccDNA were

evaluated by qPCR and Southern blot assays in HepaRG and HBV-infected HepaRG cells treated with pcDNA3.1 or pcDNA3.1-PCNAP1. (I and J) The EcoRI digested-DNA and EcoRI undigested-DNA were examined by Southern blot analysis to verify the cccDNA and rcDNA in the cells. Error bars represent means ± SD (n=3). (K) Primary human hepatocytes (PHH) were infected with HBV and transfected with pcDNA3.1 or pcDNA3.1-PCNAP1. The levels of HBV DNA and cccDNA were measured by qPCR in the cells. (L) PHH were infected with HBV and transfected with siRNA targeting PCNAP1 or a negative control siRNA. The levels of HBV DNA and cccDNA were measured by qPCR in the cells. (M) The HepaRG cells were infected with HBV and transfected with siRNA targeting PCNAP1 or a negative control siRNA. The levels of HBV DNA and cccDNA were determined by qPCR in the cells. (N) The levels of HBV DNA and cccDNA were measured by qPCR in the HepG2.2.15 cells transfected with pcDNA3.1 or pcDNA3.1-PCNAP1. Statistical significant differences are indicated: *$P<0.05$; **$P<0.01$; ***$P<0.001$; Student's *t* test.

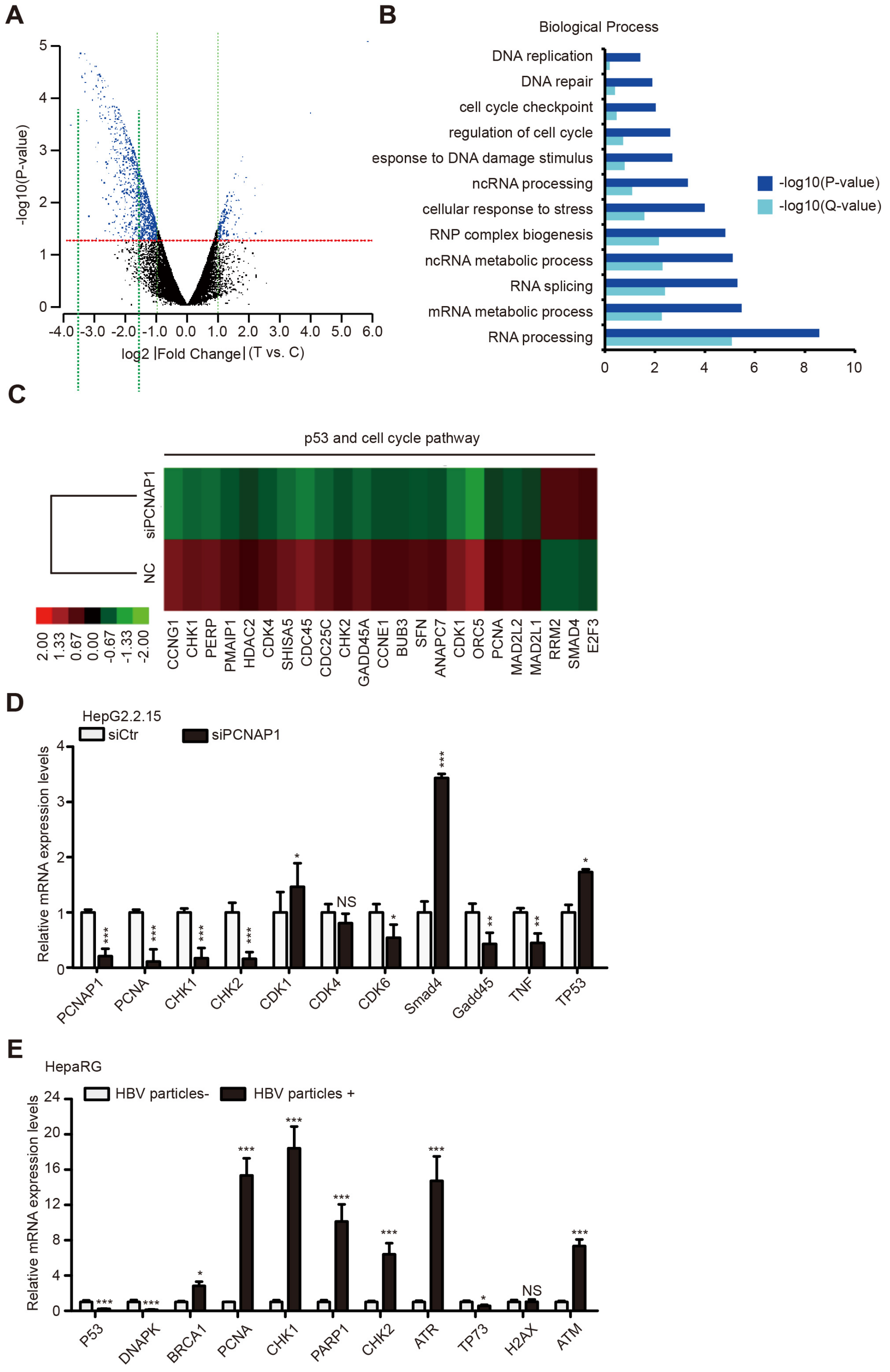

Figure S3. The global impact of PCNAP1 on host gene expression profiling in the presence of HBV. (A) Volcano plots depicted the relative fold change of gene expression in the

microarray when HepG2.2.15 cells were treated with siCtr and siPCNAP1. Up- and down-regulated genes were represented, respectively. (B) Gene ontology (GO) biological process analysis of the overlapping genes regulated by PCNAP1. The GO analysis was based on the DE list. (C) Heatmaps demonstrated the relative fold change of gene expression based on the cellular DNA damage repair and p53 pathway related genes by microarray. Red and green indicate increased and decreased expression, respectively. (D) Genes regulated by the microarray in HepG2.2.15 cells treated with siCtr and siPCNAP1 were confirmed by RT-qPCR. (E) The relative mRNA expression level of genes in DNA damage repair signaling were confirmed by RT-qPCR in HepaRG cells infected with or without HBV. Error bars represent means ± SD (n=3). Statistical significant differences are indicated: $*P<0.05$; $**P<0.01$; $***P<0.001$; NS, no significance; Student's *t* test.

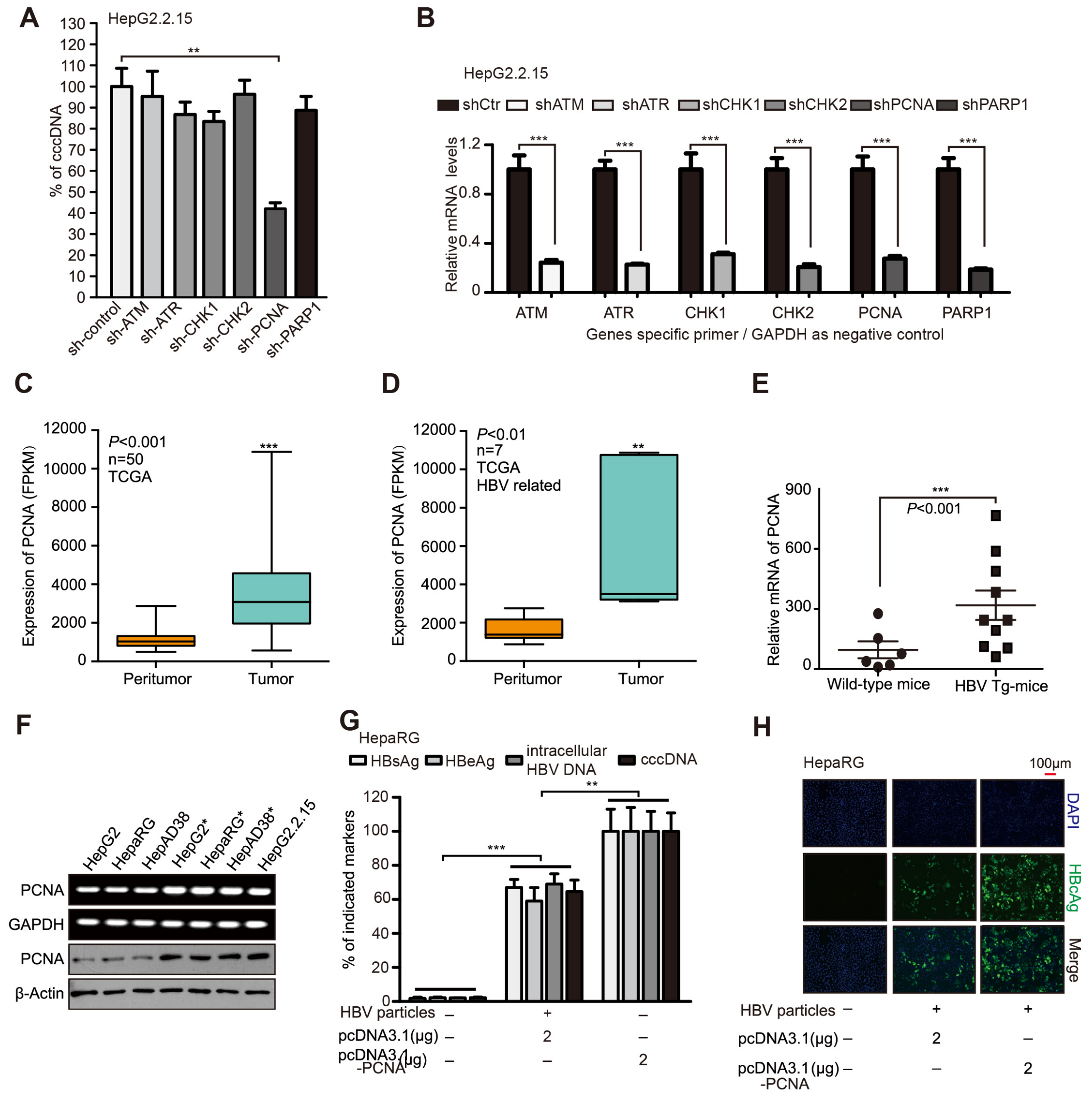
A
HepG2.2.15
% of cccDNA
sh-control
sh-ATM
sh-ATR
sh-CHK1
sh-CHK2
sh-PCNA
sh-PARP1
B
HepG2.2.15
shCtr shATM shATR shCHK1 shCHK2 shPCNA shPARP1
Relative mRNA levels
ATM ATR CHK1 CHK2 PCNA PARP1
Genes specific primer / GAPDH as negative control
C
P<0.001
n=50
TCGA
Expression of PCNA (FPKM)
Peritumor
Tumor
D
P<0.01
n=7
TCGA
HBV related
Expression of PCNA (FPKM)
Peritumor
Tumor
E
P<0.001
Relative mRNA of PCNA
Wild-type mice
HBV Tg-mice
F
HepG2
HepaRG
HepAD38
HepG2*
HepaRG*
HepAD38*
HepG2.2.15
PCNA
GAPDH
PCNA
β-Actin
G
HepaRG
HBsAg HBeAg intracellular HBV DNA cccDNA
% of indicated markers
HBV particles
pcDNA3.1(μg)
pcDNA3.1(μg) -PCNA
H
HepaRG
100μm
DAPI
HBcAg
Merge
HBV particles
pcDNA3.1(μg)
pcDNA3.1(μg) -PCNA

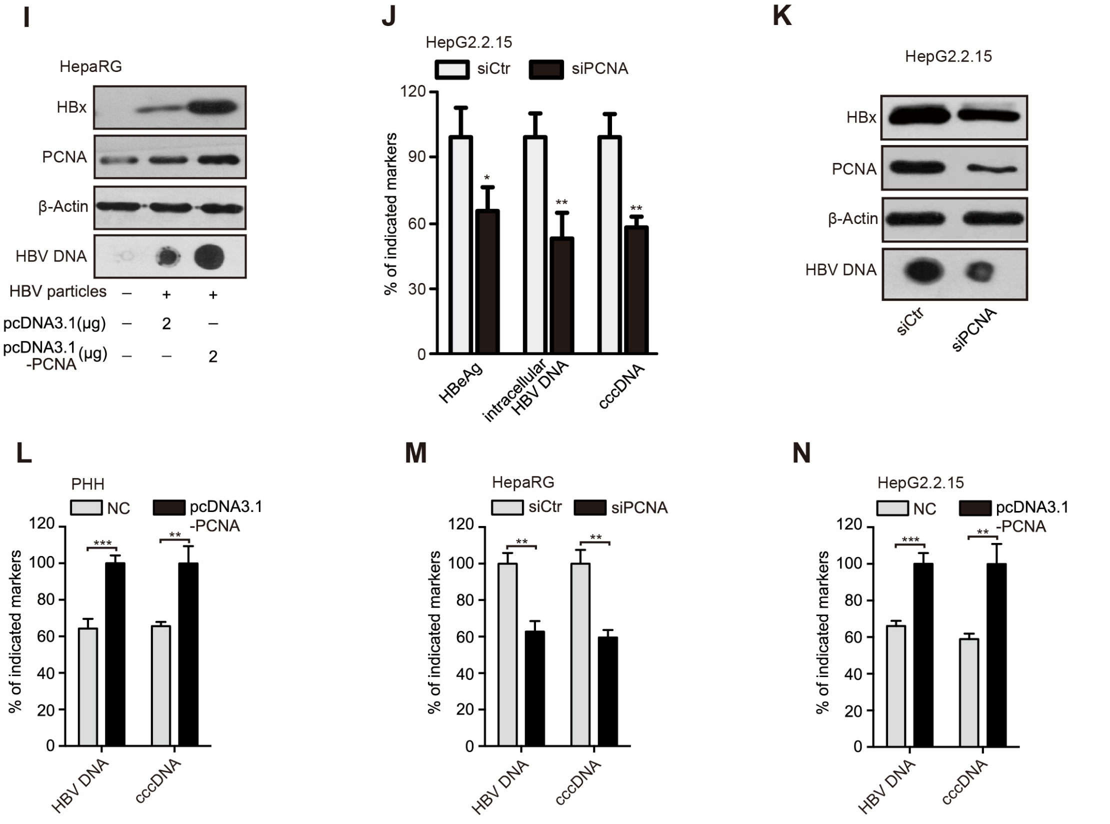

Figure S4. PCNAP1 contributes to HBV replicating and cccDNA accumulation through its ancestor PCNA. (A and B) shRNAs targeting different cellular DNA factors were transfected into HepG2.2.15 cells. The levels of HBV cccDNA and the efficiency of the shRNAs were measured by qPCR. (C) Expression of PCNA in 50 paired tumors and their adjacent nontumor parts of HCC patients in TCGA database. (D) Expression of PCNA in 7 paired tumors and their adjacent nontumor parts of HBV-related HCC patients in TCGA database. (E) The relative mRNA levels of PCNA were examined by RT-qPCR in liver tissues of wild type (n=6) and HBV-Tg mice (n=10) (***P*<0.001, Mann-Whitney U test). (F) The expression levels of PCNA in HBV free or HBV positive hepatoma cells were detected by RT-PCR and Western blot assays. The expression of PCNA was normalized to GAPDH and β-Actin. (G-I) In HepaRG and HBV-infected HepaRG cells treated with pcDNA3.1 or pcDNA3.1-PCNA, the levels of PCNA, HBx, HBcAg, HBsAg, HBeAg, HBV progeny DNA, intracellular HBV

DNA and cccDNA were measured by ELISA, qPCR, immunofluorescence staining and Western blot analysis or Dot blot assays. (J and K) The levels of HBeAg, intracellular HBV DNA, cccDNA, PCNA, HBx, and HBV progeny DNA were analysis by ELISA, qPCR, Western blot analysis or Dot blot assays in HepG2.2.15 cells transfected with sicontrol (siCtr) or siPCNA. (L) PHH were infected with HBV and transfected with pcDNA3.1 or pcDNA3.1-PCNA. The levels of HBV DNA and cccDNA were assessed by qPCR in the cells. (M) The HepaRG cells were infected with HBV and transfected with siRNA targeting PCNA or a negative control siRNA. The levels of HBV DNA and cccDNA were measured by qPCR in the cells. (N) The levels of HBV DNA and cccDNA were examined by qPCR in the HepG2.2.15 cells transfected with pcDNA3.1 or pcDNA3.1-PCNA. Error bars represent means ± SD (n=3). Statistical significant differences are indicated: *$P<0.05$; **$P<0.01$; ***$P<0.001$; Student's *t* test.

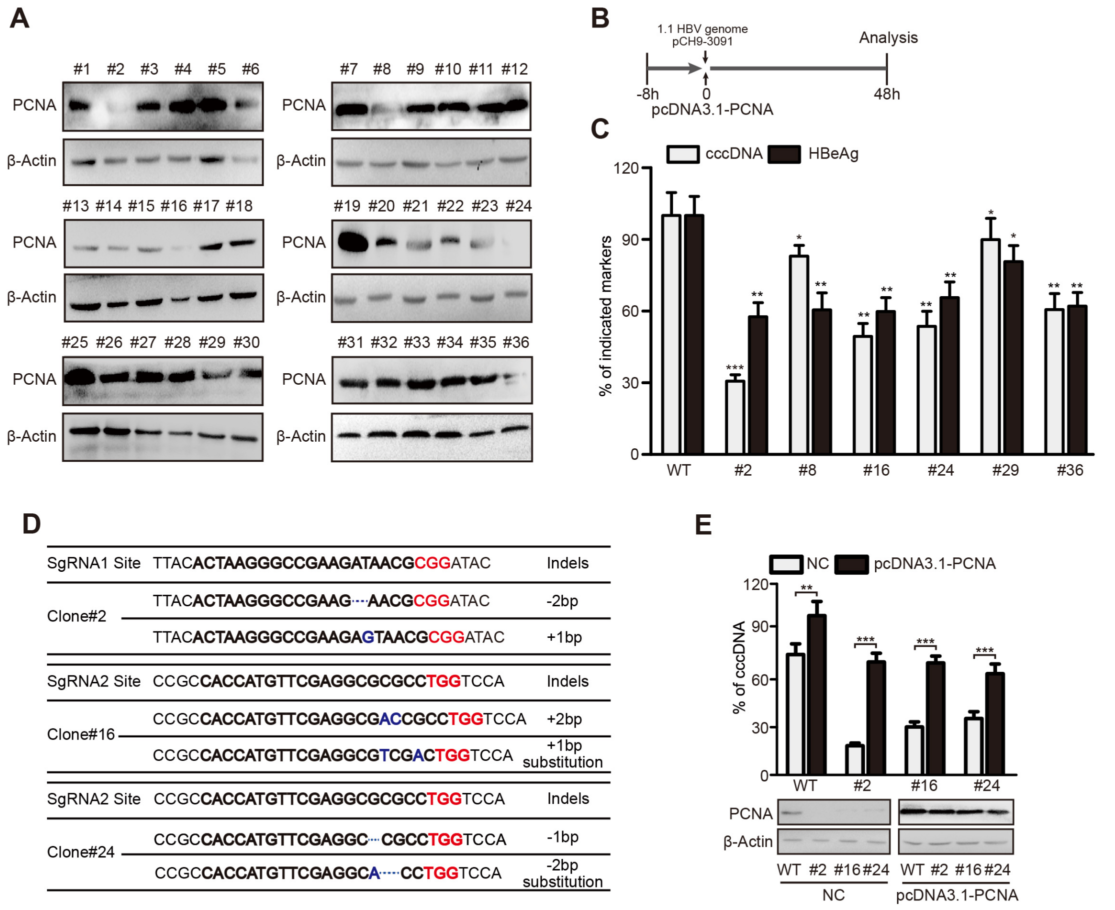

Figure S5. Depletion of PCNA impairs accumulation of cccDNA. (A) The expression of PCNA was measured by Western blot analysis in 36 cell clones derived from HepG2 cells (#1-#18 clones came from the sg1-selected cells and #19-#36 clones from the sg2-selected cells). (B) The scheme shows the procedure of treatment in HepG2#2 cells transfected with pcDNA3.1-PCNA. (C) HBV indicated markers including cccDNA and HBeAg were assessed by qPCR and ELISA assays in #2, #8, #16, #24, #29, and #36 cell clones, respectively. (D) Three clones were selected by length polymorphisms of PCR products and validated by Sanger sequencing. Clone 2 and Clone 16, Clone 24 were respectively generated by SgRNA1 and SgRNA2 that produced Clone2 with 2-bp deletion and 1+bp insertion, 2+bp insertion and 1+bp insertion/substitution for Clone 16 and 1-bp deletion and 2-bp deletion/substitution of Clone 24 for two alleles at different positions. (E) The levels of HBV cccDNA were

measured by qPCR in the indicated cells transfected with pcDNA3.1-PCNA. Error bars represent means ± SD (n=3). Statistical significant differences are indicated: *P<0.05; **P<0.01; ***P<0.001; Student's t test.

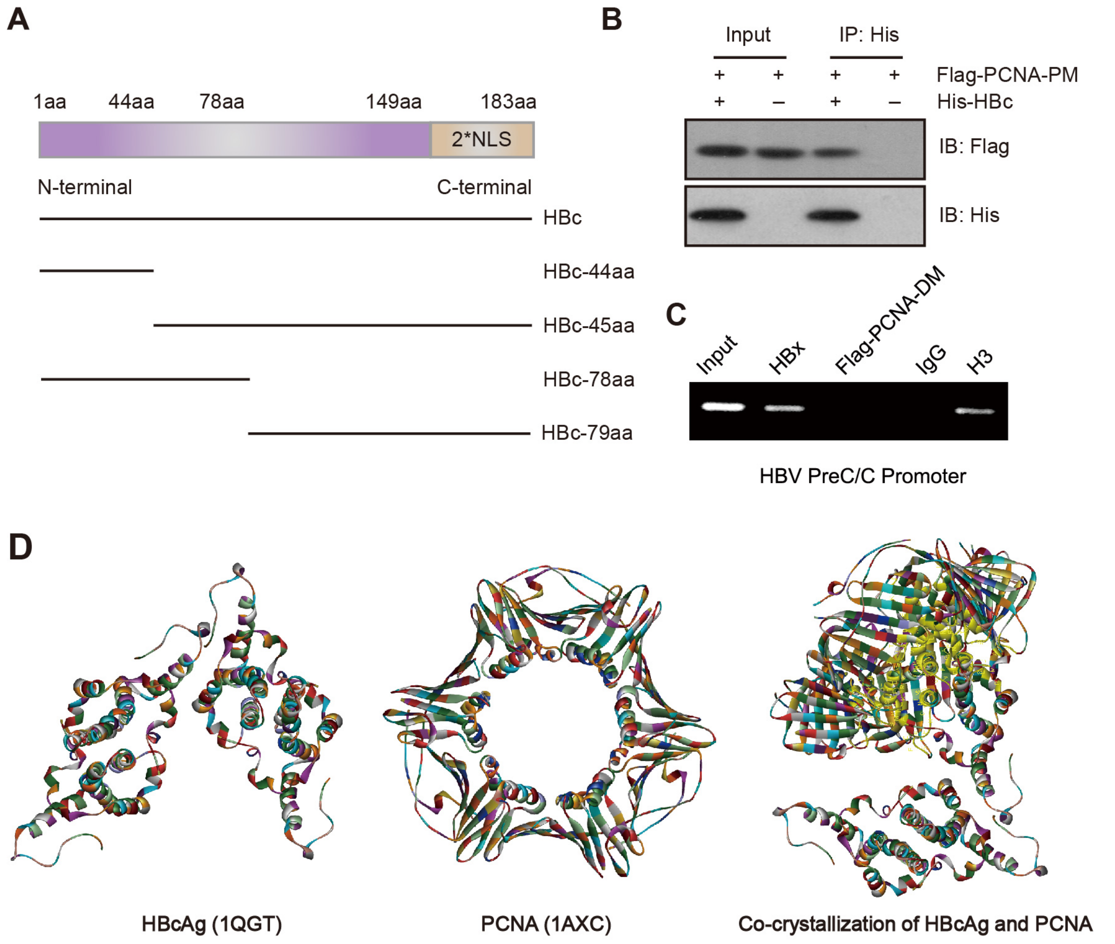

Figure S6. PCNA anchors onto cccDNA minichromosome through interacting with HBc. (A) The scheme of serial HBc mutants which were fused to 6xHis at C-terminus. (B) The interaction between serial HBc mutants and PCNA was identified by co-IP assays in HepG2 cells. (C) Cross-linked chromatin from HepAD38 cells transfected with flag-PCNA-DM was exogenously immunoprecipitated with the relevant control IgG or specific anti-HBx, anti-H3, and anti-flag antibodies and analyzed by PCR with HBV cccDNA selective primers. (D) A

three-dimensional co-crystallization structure of HBV core protein with PCNA has been generated by automatic modeling mode. The potential interaction sites (yellow) of modeled PCNA and HBc were depicted, in which the main domain of PCNA interacting with HBc was located in the α-helix including amino acid 8-11, 13, 14, 17, 20-21, 76-85, 141-146 and the main domain of HBc was located in the α-helix of C-terminus including amino acid 80-92, 107-130, 133, 142-143.

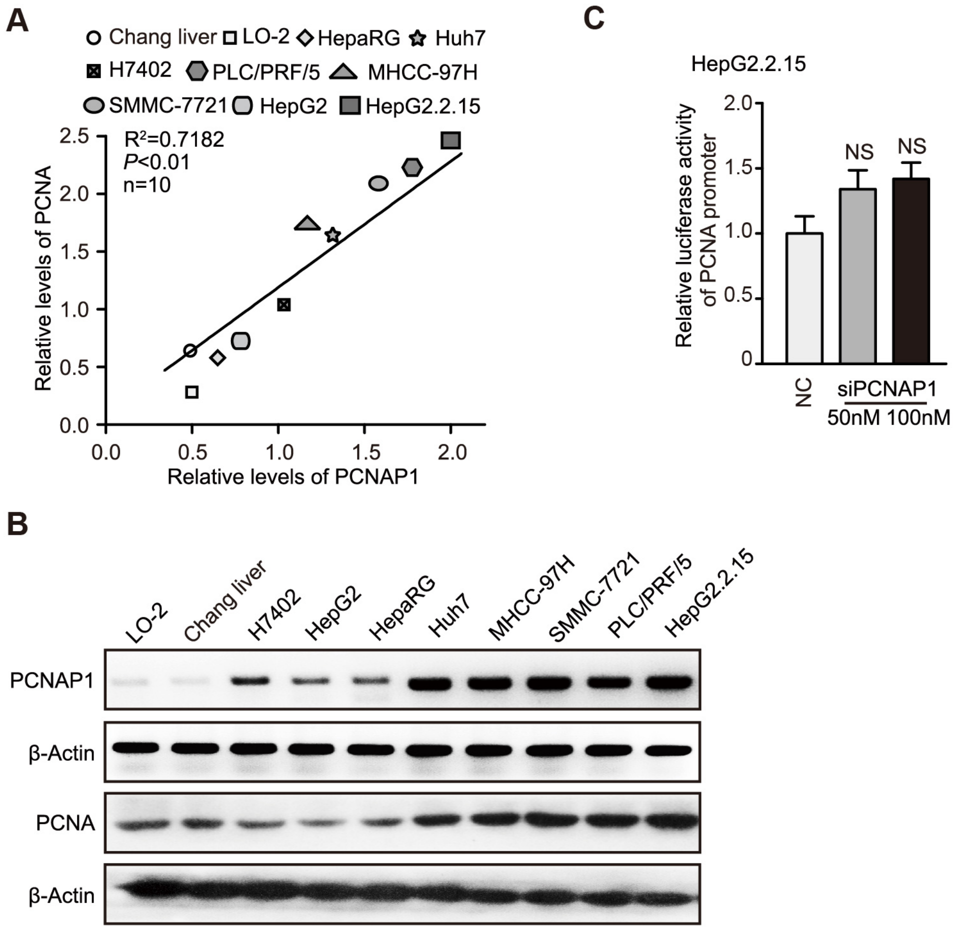

Figure S7. PCNAP1 stimulates the expression of PCNA at post-transcriptional level in hepatoma cells. (A) Correlation between the PCNAP1 RNA levels and the PCNA mRNA levels was examined by RT-qPCR in 10 liver cell lines. (B) The expression levels of PCNAP1 and PCNA were detected by RT-qPCR and Western blot analysis in 10 liver cell

lines, respectively. (C) Luciferase activities of PCNA promoter were measured by luciferase reporter gene assays in HepG2.2.15 cells transfected with siPCNAP1. Error bars represent means ± SD (n=3). Statistical significant differences are indicated: NS, no significance; Student's *t* test.

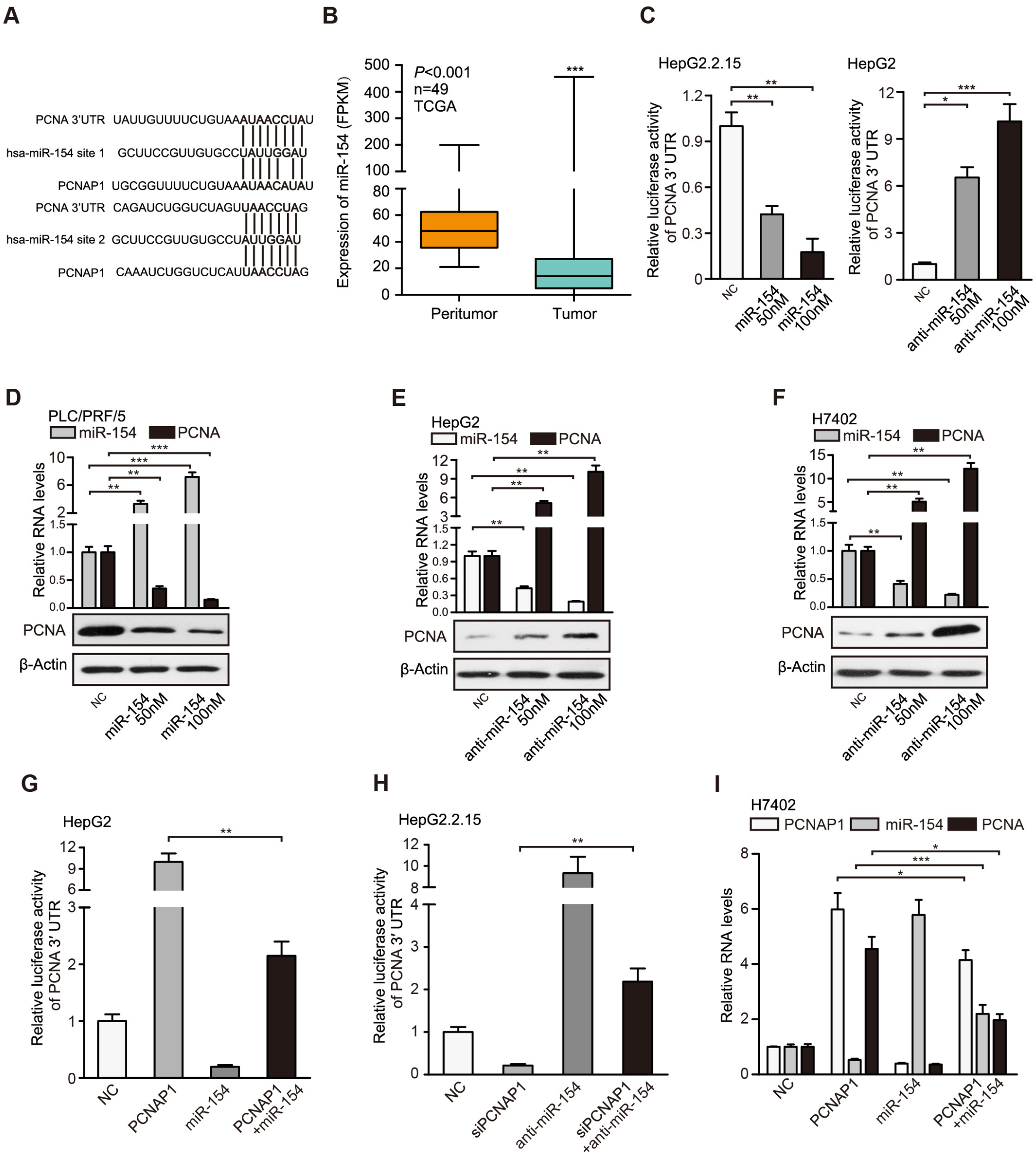

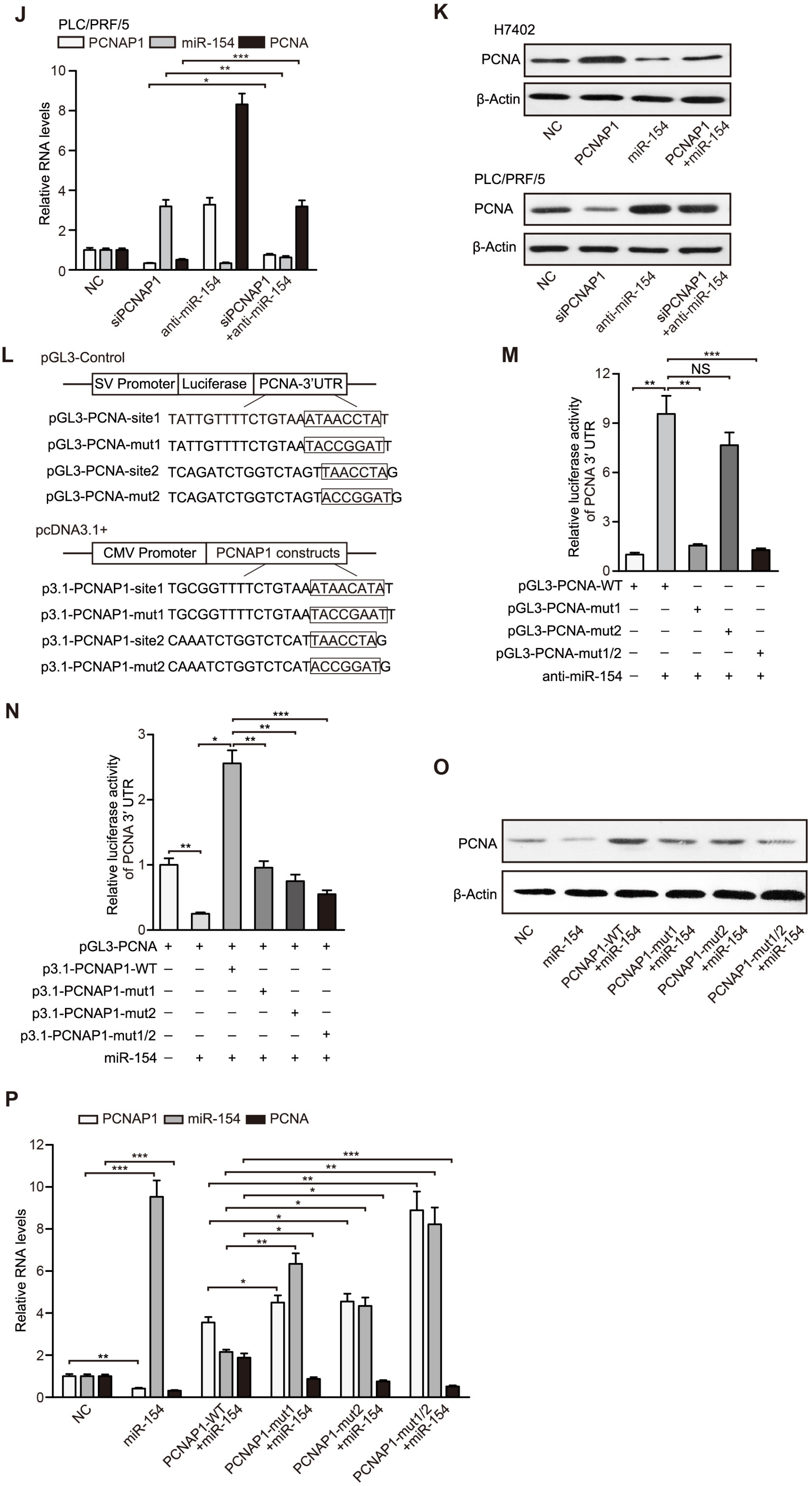
J
PLC/PRF/5
PCNAP1
miR-154
PCNA
Relative RNA levels
NC
siPCNAP1
anti-miR-154
siPCNAP1 +anti-miR-154
K
H7402
PCNA
β-Actin
NC
PCNAP1
miR-154
PCNAP1 +miR-154
PLC/PRF/5
PCNA
β-Actin
NC
siPCNAP1
anti-miR-154
siPCNAP1 +anti-miR-154
L
pGL3-Control
SV Promoter
Luciferase
PCNA-3'UTR
pGL3-PCNA-site1 TATTGTTTTCTGTAAATAACCTAT
pGL3-PCNA-mut1 TATTGTTTTCTGTAATACCGGATT
pGL3-PCNA-site2 TCAGATCTGGTCTAGTTAACCTAG
pGL3-PCNA-mut2 TCAGATCTGGTCTAGTACCGGATG
pcDNA3.1+
CMV Promoter
PCNAP1 constructs
p3.1-PCNAP1-site1 TGCGGTTTTCTGTAAATAACATAT
p3.1-PCNAP1-mut1 TGCGGTTTTCTGTAATACCGAATT
p3.1-PCNAP1-site2 CAAATCTGGTCTCATTAACCTAG
p3.1-PCNAP1-mut2 CAAATCTGGTCTCATACCGGATG
M
Relative luciferase activity of PCNA 3' UTR
NS
pGL3-PCNA-WT
pGL3-PCNA-mut1
pGL3-PCNA-mut2
pGL3-PCNA-mut1/2
anti-miR-154
N
Relative luciferase activity of PCNA 3' UTR
pGL3-PCNA
p3.1-PCNAP1-WT
p3.1-PCNAP1-mut1
p3.1-PCNAP1-mut2
p3.1-PCNAP1-mut1/2
miR-154
O
PCNA
β-Actin
NC
miR-154
PCNAP1-WT +miR-154
PCNAP1-mut1 +miR-154
PCNAP1-mut2 +miR-154
PCNAP1-mut1/2 +miR-154
P
PCNAP1
miR-154
PCNA
Relative RNA levels
NC
miR-154
PCNAP1-WT +miR-154
PCNAP1-mut1 +miR-154
PCNAP1-mut2 +miR-154
PCNAP1-mut1/2 +miR-154

Figure S8. PCNAP1 up-regulates PCNA through competing for miR-154. (A) The binding sites of miR-154 in PCNA mRNA 3′UTR and PCNAP1 were shown. (B) Expression of miR-154 in 49 paired tumors and their adjacent nontumor parts of HCC patients was showed in TCGA database. (C) Luciferase activities of PCNA mRNA 3′UTR were tested in HepG2.2.15 cells transfected with miRNA control or miR-154, and in HepG2 cells transfected with control RNA and miR-154 inhibitors. (D-F) The relative levels of miR-154 and PCNA were examined by RT-qPCR and Western blot analysis in PLC/PRF/5 cells transfected with miR-154, and in HepG2 and H7402 cells transfected with miR-154 inhibitors, respectively. (G) Luciferase activities of PCNA mRNA 3′UTR were measured by luciferase reporter gene assays in HepG2 cells with the single treatment of PCNAP1 or miR-154 and the co-transfection with PCNAP1 and miR-154. (H) Luciferase activities of PCNA mRNA 3′UTR were measured as above in HepG2.2.15 cells with the single treatment of siPCNAP1 or anti-miR-154 and the co-transfection with siPCNAP1 and anti-miR-154. (I and J) The RNA levels of PCNAP1, miR-154 and PCNA were measured by RT-qPCR in H7402 and PLC/PRF/5 cells transfected with different regiments. (K) The protein levels of PCNA were detected by Western blot analysis in the cells with different treatments as above. (L) The binding sites of miR-154 in PCNA mRNA 3′UTR and PCNAP1 RNA region were shown and the mutants were generated at the regions as above indicated. The constructs of PCNA mRNA 3′UTR and PCNAP1 sequence were cloned into pGL3-control and pcDNA3.1+ plasmids, respectively. (M) Luciferase activities of mutants in PCNA mRNA 3′UTR were measured by luciferase reporter gene assays in HepG2 cells treated with different regiments. (N) The effect of miR-154 and the different PCNAP1 constructs on the luciferase activities of PCNA mRNA

3′UTR was examined by luciferase reporter gene assays in HepG2.2.15 cells, respectively. (O) The expression levels of PCNA were tested by Western blot analysis in HepG2.2.15 cells with different regiments as above. (P) The effect of miR-154 and the different PCNAP1 constructs on the expression levels of PCNA was examined by RT-qPCR in HepG2.2.15 cells. Error bars represent means ± SD (n=3). Statistical significant differences are indicated: **P*<0.05; ***P*<0.01; ****P*<0.001; NS, no significance, Student′s *t* test.

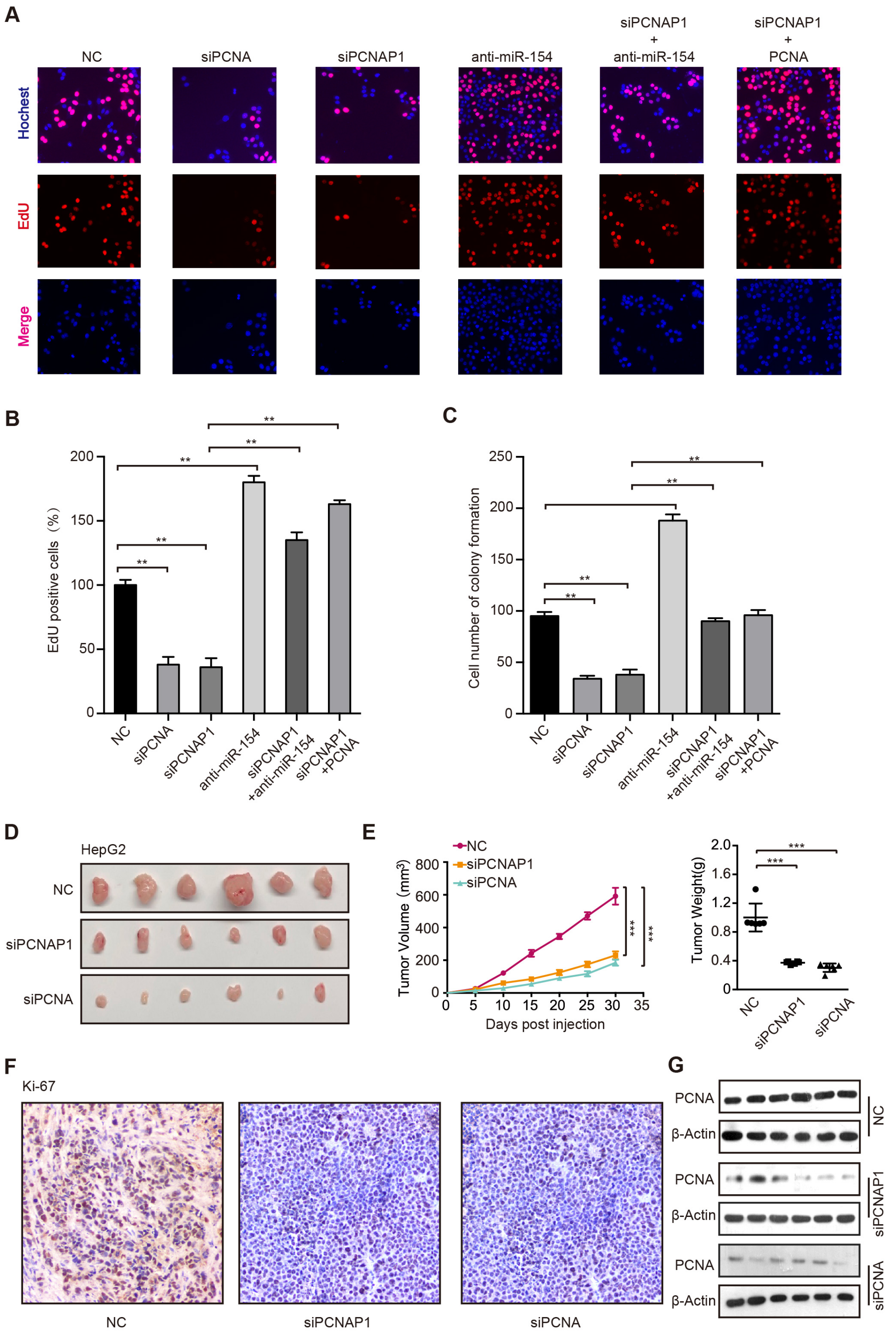

Figure S9. PCNAP1 and PCNA promotes the growth of hepatoma cells *in vitro.* (A and B) EdU incorporation assays were applied to examine the EdU positive cells in HepG2.2.15 cells

transfected with indicated regiments. (C) Colony formation efficiencies were determined in HepG2.2.15 cells treated with different regiments as above. (D) Photographs of dissected tumors from nude mice injected with HepG2 cells treated with siCtr, siPCNAP1 or siPCNA. (E) The average volume and weight of the tumors transplanted with HepG2 cells pre-treated with siCtr, siPCNAP1 or siPCNA in nude mice. (F) Immunohistochemistry assays for Ki-67 were measured in tumor tissues from the nude mice treated as above. (G) The protein expression levels of PCNA were examined by Western blot analysis in the tumor tissues from nude mice, respectively. Error bars represent means ± SD (n=3). Statistical significant differences are indicated: ***P*<0.01; ****P*<0.001, Student's *t* test.

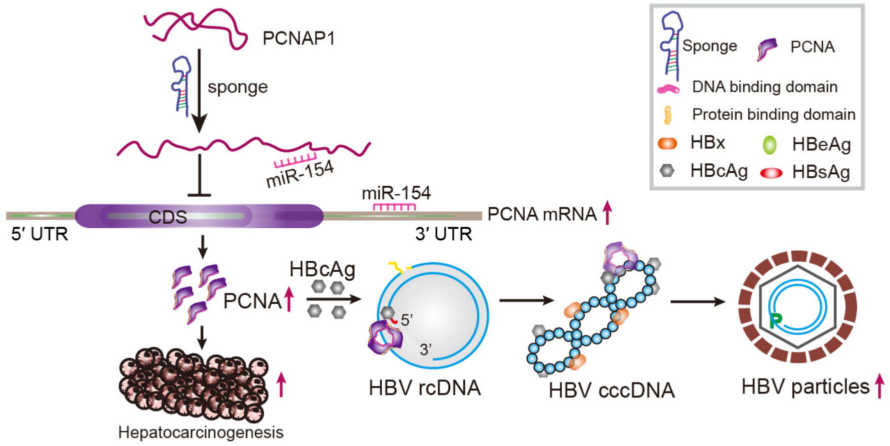

Figure S10. A model of PCNAP modulating HBV replication and enhancing hepatocarcinogenesis. In this model, PCNAP1/PCNA signaling promotes HBV replication by modulating HBV cccDNA accumulation. PCNAP1 as a natural sponge of miR-154 regulates its ancestral PCNA. HBc-recruited PCNA binds to HBV cccDNA, leading to HBV replication and cccDNA accumulation. Meanwhile, PCNAP1/PCNA signaling promotes hepatocarcinogenesis.

Table S1. Information of human liver-chimeric mice

Group	Mouse (No.)	Albumin (μg/mL)	HBV (IU/mL)
Control	10.16-14	1916.18	—
	10.16-15	2065.83	—
	10.16-19	3242.68	—
HBV infection	7.25-13	1093.72	7.81E+05
	7.25-15	1571.23	4.20E+06
	8.10-15	630.57	5.82E+06

Table S2. The characteristics of patients

No.	Age	Gender	Organ	HBV	cccDNA
1	59	F	Liver	+	+
2	60	M	Liver	+	-
3	65	M	Liver	+	+
4	43	M	Liver	+	+
5	60	F	Liver	+	-
6	41	M	Liver	+	+
7	45	M	Liver	+	+
8	56	M	Liver	+	-
9	70	M	Liver	+	-
10	67	M	Liver	+	+
11	59	M	Liver	+	+
12	57	M	Liver	+	+
13	61	F	Liver	+	-
14	51	F	Liver	+	+
15	56	M	Liver	+	+
16	60	M	Liver	+	-
17	54	M	Liver	+	+
18	36	M	Liver	+	-
19	46	M	Liver	+	+
20	60	M	Liver	+	+
21	59	M	Liver	+	-
22	57	F	Liver	+	+
23	51	M	Liver	+	-
24	38	M	Liver	+	+
25	56	M	Liver	+	-
26	49	M	Liver	+	+
27	58	F	Liver	+	-
28	46	M	Liver	+	+
29	56	M	Liver	+	+
30	54	M	Liver	+	-
31	39	M	Liver	+	-
32	53	M	Liver	+	+
33	60	M	Liver	+	+

34	66	M	Liver	+	-
35	60	F	Liver	+	+
36	59	M	Liver	+	-
37	69	M	Liver	+	+
38	38	M	Liver	+	+
39	56	M	Liver	+	+
40	53	F	Liver	-	-
41	47	M	Liver	-	-
42	67	M	Liver	-	-
43	49	M	Liver	-	-

Table S3. List of primers used in this paper

Gene	Primer	Sequence(5′-3′)
**RT-qPCR**		
Mouse PCNA	forward	ATGTTTGAGGCACGCCTGATCCAG
	reverse	CTAAGATGCTTCCTCACTTCAATC
Mouse GAPDH	forward	CCTGCCAAGTATGATGACAT
	reverse	GTTGCTGTAGCCGTATTCA
PCNA	forward	AACCTCACCAGTATGTCC
	reverse	CCATTTCCAAGTTCTCCAC
HBx	forward	ATGGCTGCTAGGCTGTGC
	reverse	TTAGGCAGAGGGGAAAAAGTTG
PCNAP1	forward	CACTCCACTCTCTCTTCC
	reverse	CAGAAAACCGCATCTACC
ATM	forward	CAGGGTAGTTTAGTTGAGGTTGACAG
	reverse	CTATACTGGTGGTCAGTGCCAAAGT
ATR	forward	TGTCTGTACTCTTCACGGCATGTT
	reverse	AAGAGGTCCACATGTCCGTGTT
CHK1	forward	GGTGAATATAGTGCTGCTATGTTGACA
	reverse	TTGGATAAACAGGGAAGTGAACAC
CHK2	forward	CCCAAGGCTCCTCCTCACA
	reverse	AGTGAGAGGACTGGCTGGAGTT
P53	forward	GGAGGTTGGCTCTGACTGTACC
	reverse	TCCGTCCCAGTAGATTACCAC
TP73	forward	GCACCACGTTTGAGCACCTCTGG
	reverse	AGATGTAGTCATGCCCTCCAGGTG
H2AX	forward	ACTCAACTCGGCAATCCAAG
	reverse	GGGTTAGCTGCAGAATTCCA
DNAPK	forward	AAAATTGGTACCCCCGACAG
	reverse	TTCTCATGACCCAGGAGTAGC
PARP1	forward	GCCCTAAAGGCTCAGAACGACC
	reverse	TCCAAGATCGCCGACTCCC
BRCA1	forward	AGCTCGCTGAGACTTCCTGGA
	reverse	CAATTCAATGTAGACAGACGT
Gadd45a	forward	TGGTGACGAATCCACATTCA
	reverse	CACTGGAACCCATTGATCC
CDK1	forward	TCCCTCCTGGTCAGTACATGG
	reverse	ACAAAACACAATCCCCTGTAGG
Smad4	forward	TGAAGGAATCATTCCAGTGCTAG
	reverse	CTTGGTAAAATTAACTCACCCACA
CDK4	forward	ATGGCTACCTCTCGATATGAGC
	reverse	CATTGGGGACTCTCACACTCT
CDK6	forward	GCTGACCAGCAGTACGAATG
	reverse	GCACACATCAAACAACCTGACC
TNF	forward	CCTCTCTCTAATCAGCCCTCTG

	reverse	GAGGACCTGGGAGTAGATGAG
18S	forward	AACCCGTTGAACCCCATT
	reverse	CCATCCAATCGGTAGTAGCG
GAPDH	forward	AACGGATTTGGTCGTATTG
	reverse	GGAAGATGGTGATGGGATT
miR-154	forward	TAGGTTATCCGTGTTGCCTTCG
	reverse	GCGAGCACAGAATTAATACGAC
U6	forward	CTCGCTTCGGCAGCACA
	reverse	AACGCTTCACGAATTTGCGT
**Luciferase assay**		
pGL3-PCNA-3'UTR	forward	TGCTCTAGAGCATTCTTAAAATTCAAGAAAA
	reverse	GGGGGCCGGCCTGATGTTTGAAATTCAAGTAACT
pGL3-PCNA-promoter	forward	GGGGTACCTAAAACCAATCCCTACCTTCCTTAA
	reverse	CCCTCGAGTGGCGCTTCTCGCCCTC
pGL3-PCNA-mut1	forward	TATTGTTTTCTGTAATACCGGATTTTTTTTCTC
	reverse	GAGAAAAAAAATCCGGTATTACAGAAAACAATA
pGL3-PCNA-mut2	forward	ATCTGGTCTAGTATCGGACGAAGTATTTTTGTC
	reverse	GACAAAAATACTTCGTCCGATACTAGACCAGAT
**CHIP assay**		
PreC/HBc promoter	forward	CTGAATCCTGCGGACGACCC
	reverse	CCCAAGGCACAGCTTGGAGG
**Plasmid construction**		
pcDNA3.1-PCNA	forward	CGGGGTACCATGTTCGAGGCGCGCCTGGT
	reverse	CCGCTCGAGCTAAGATCCTTCTTCATCCTCGATC
pcDNA3.1-PCNAP1	forward	GGGGTACCAGGCCTGCTGGGACAAC
	reverse	CGGAATTCTTTAAAATTCAGATTAAACTTTATT
pcDNA3.1-PCNAP2	forward	GGGGTACCAGCAGGCTTGTGGAAAACATGAAAACT
	reverse	CGGAATTCAATAGCATTAATTAATTCATGAAGGT
pcDNA3.1-PCNAP3	forward	CCCATATGGTTGTCATTCTAGGTCTCAGCCT
	reverse	CGGGATCCAGATCTGACTGACTTTGGACTTTA
pcDNA3.1-PCNAP4	forward	GGGGTACCATGTTCGAGGTACACCTGGTCCAGG
	reverse	CGGAATTCAAAAAAAAGTCCCATATTAGCAATGTT
pcDNA3.1-PCNAP1-mut1	forward	TGCGGTTTTCTGTAAGCCCAGACT
	reverse	AGTCTGGGCTTACAGAAAACCGCA
pcDNA3.1-PCNAP1-mut2	forward	CAAATCTGGTCTCATTAACCTAG
	reverse	CTAGGTTAATGAGACCAGATTTG
pCMV-PCNA-WT	forward	ATAGGATCCATGTTCGAGGCGCGCCTGGT
	reverse	GCACTCGAGAGATCCTTCTTCATCCTCGATCTTGG
pCMV-PCNA-DM	forward	TGCCGGCGCAGTAGGTGTCGAAGCCCTCAGACCGCA
	reverse	CGACACCTACTGCGCCGGCAATGAAGATATCATTACAC
pCMV-PCNA-PM	forward	TCTTTACTACCATTTCATAGTCTGAAACTTTCTCCTG
	reverse	CTATGAAATGGTAGTAAAGATGCCTTCTGGTGAATTTG
pcDNA4.0-HBc-WT	forward	ACGGGATCCATGGACATCGACCCTTATAAAGA
	reverse	CCGCTCGAGACATTGAGATTCCCGAGATTGA

pcDNA4.0-HBc (1-44)	forward	ACGGGATCCATGGACATCGACCCTTATAAAGA
	reverse	CCGCTCGAGAGACTCTAAGGCTTCCCGATACA
pcDNA4.0-HBc (1-78)	forward	ACGGGATCCATGGACATCGACCCTTATAAAGA
	reverse	CCGCTCGAGATCTTCCAAATTAACACCCACCC
pcDNA4.0-HBc (45-183)	forward	ACGGGATCCATGCCTGAGCATTGTTCACCTCACC
	reverse	CCGCTCGAGACATTGAGATTCCCGAGATTGA
pcDNA4.0-HBc (79-183)	forward	ACGGGATCCATGCCAGCGTCTAGAGACCTAGTAG
	reverse	CCGCTCGAGACATTGAGATTCCCGAGATTGA
PCH9/3091-HBx-mut1	forward	GCGTCGACGCACCATGCAACTTTTTCACCTC
	reverse	CAGCCTAGCAGCTGTGGAAACGATGT
PCH9/3091-HBx-mut2	forward	ACATCGTTTCCACAGCTGCTAGGCTG
	reverse	CGCCCGGGGATCTCGTACTGAAGGAAAGAAG
pmCherry-C1-PCNA	forward	GCGTCGACATGTTCGAGGCGCGCCTG
	reverse	CGGGATCCCTAAGATCCTTCTTCATCCTCGATC
pEGFP-n1-HBc	forward	CGGCTAGCATGGACATTGACCCGTAT
	reverse	CCAAGCTTACATTGAGATTCCCGAGAT
pcDNA3.1-HBx	forward	CCCAAGCTTATGGCTGCTAGGCTGTGCT
	reverse	CCGCTCGAGTTAGGCAGAGGTGAAAAAGTTG
**CRISPR**		
PCNA-sgRNA-1	forward	CACCGACTAAGGGCCGAAGATAACG
	reverse	AAACCGTTATCTTCGGCCCTTAGTC
PCNA-sgRNA-2	forward	CACCGGCGCGCCTCGAACATGGTGG
	reverse	AAACCCACCATGTTCGAGGCGCGCC
PCNA-sanger-PCR-1	forward	GTCTCCTGTCTTCTAGTATGTCC
	reverse	CTATGACATAACTATCGTGCCTGTG
PCNA-sanger-PCR-2	forward	CATGGACACGATTGGCCCTAAAGTC
	reverse	GGTTTACACCGCTGGAGCTAATATC
**HBV cccDNA assay**		
cccDNA	forward	GCCTATTGATTGGAAAGTATGT
	reverse	AGCTGAGGCGGTATCTA
rcDNA	forward	GTTGCCCGTTTGTCCTCTAATTC
	reverse	GGAGGGATACATAGAGGTTCCTTGA
**pSilencer-construction**		
pSilencer-ATM	forward	GATCCGAAGGAAGCCAGAGTACAATTCAAGAGATTGTACTCTGGCTTCCTTCTTTTTA
	reverse	AGCTTAAAAAGAAGGAAGCCAGAGTACAATCTCTTGAATTGTACTCTGGCTTCCTTCG
pSilencer-ATR	forward	GATCCGGTCAGCTGTCTACTGTTATTCAAGAGATAACAGTAGACAGCTGACCTTTTTA
	reverse	AGCTTAAAAAGGTCAGCTGTCTACTGTTATCTCTTGAATAACAGTAGACAGCTGACCG
pSilencer-CHK1	forward	GATCCAAGGGATAACCTCAAAATCTCTTCAAGAGAGAGATTTTGAGGTTATCCCTTTTTTTA
	reverse	AGCTTAAAAAAAGGGATAACCTCAAAATCTCTCTCTTGAAGAGATTTTGAGGTTATCCCTTG
pSilencer-CHK2	forward	GATCCGAGGACTGTCTTATAAAGATTTTCAAGAGAAATCTTTATAAGACAGTCCTCTTTTTA
	reverse	AGCTTAAAAAGAGGACTGTCTTATAAAGATTTCTCTTGAAAATCTTTATAAGACAGTCCTCG
pSilencer-PARP1	forward	GATCCGGAGGCAAGTTGACAGGATCTTTCAAGAGAAGATCCTGTCAACTTGCCTCCTTTTTA
	reverse	AGCTTAAAAAGGAGGCAAGTTGACAGGATCTTCTCTTGAAAGATCCTGTCAACTTGCCTCCG
pSilencer-PCNA	forward	GATCCGATCGAGGATGAAGAAGGATTCAAGAGATCCTTCTTCATCCTCGATCTTTTTA

	reverse	AGCTTAAAAAGATCGAGGATGAAGAAGGATCTCTTGAATCCTTCTTCATCCTCGATCG

Table S4. List of antibody used in this paper

Gene	Manufacture	Catalog Number	RRID Number
PCNA Polyclonal Antibody	Proteintech	10205-2-AP	AB_2160330
HBx Polyclonal Antibody	Abcam	ab39716	AB_880382
HBc Monoclonal Antibody	Abcam	ab8638	AB_306685
IgG Polyclonal Antibody	Abcam	ab6789	AB_955439
Flag tag Monoclonal Antibody	Sigma-Aldrich	SAB4200071	AB_10603396
His tag Monoclonal Antibody	Abcam	ab18184	AB_444306
Anti Digoxigenin AP Conjugate	Roche	11 745 832 910	N/A
Histone H3 antibody	Proteintech	17168-1-AP	N/A
Ki-67 Polyclonal Antibody	Proteintech	19972-1-AP	N/A
β-actin Monoclonal Antibody	Sigma-Aldrich	A2228	AB_476697

Table S5-1, related to Figure S3. List of genes regulated by PCNAP1

Gene_Symbol	Normalized Intensity		log2 (Ratio)	P-value
Upreggene	C[a]	T[b]	T/C	T/C
CLC	576.455078	1689.33325	1.552657	0.015617166
PSMD1	14113.9111	30434.1543	1.015531	0.026166514
NETO2	1039.28198	2802.11328	1.428792	0.012824742
CHAMP1	2692.89502	5769.89014	1.067938	0.030823622
COPRS	6065.71143	13249.8213	1.069367	0.023324579
SIAE	4719.72559	14591.4795	1.581546	0.002349261
DHCR7	1708.11768	4119.79346	1.259644	0.017256772
ZBED5	3976.9856	8241.7666	1.005716	0.035061438
ATP6V0A2	559.826538	1375.44421	1.300181	0.041219
TAF5L	2231.91577	4909.96533	1.116275	0.027351558
RC3H1	1464.23279	3906.17432	1.407222	0.01023556
PGR	70.209274	438.136749	2.429527	0.033116214
HERC3	387.074799	1353.64636	1.812656	0.015059801
LIMK1	1635.13696	3827.70215	1.217828	0.021322561
PDGFC	1898.42175	4100.17529	1.09771	0.032467507
LBH	612.483521	1496.42224	1.291802	0.038499329
PROS1	12621.9639	26885.4648	1.006092	0.027702894
TP53INP1	5757.16016	15520.0674	1.379741	0.005536321
SMAD4	4176.52783	10186.1338	1.236573	0.011962708
BCLAF1	7040.32715	16661.1855	1.188194	0.012917436
ACADSB	2622.68579	5382.97852	1.00882	0.040421493
APPBP2	1860.54565	3947.59033	1.073074	0.036491249
UBTF	954.291809	2185.23413	1.198415	0.035524588
SNCB	242.037216	1117.13965	2.217303	0.006381562
UBE2Q2	5711.89355	12107.6143	1.024896	0.029058373
LLGL1	399.08429	1207.60071	1.603818	0.021724021
WDR44	1907.65979	4445.6709	1.206349	0.020218035
SIX2	3976.06177	10303.8428	1.324491	0.008109604
TRMT1L	659.597595	2122.02051	1.683054	0.00782767
P2RY8	367.674866	1183.62317	1.696489	0.017822659
PANK1	1579.70862	4326.87256	1.443346	0.008216402
PHTF2	1382.93787	2856.60791	1.042552	0.04969389
UBE2D1	1039.28198	2641.89917	1.345497	0.018119512
CALCOCO2	1063.3009	2261.52661	1.093051	0.049098738
NDUFA2	10676.4277	23577.6426	1.064143	0.021518955
TEAD2	1372.776	4011.8938	1.539123	0.006131922
STK3	1265.6145	3073.49658	1.275864	0.020274013
ARHGAP11A	1010.64398	2273.51562	1.172805	0.037382033
TBX1	3325.70239	7304.45898	1.093639	0.02512487
DMXL1	2015.74512	4379.1875	1.103736	0.030564927
ACR	206.008774	1140.02747	2.466735	0.003887494

CCDC167	3867.97656	8188.36133	1.036864	0.03074556
FOXQ1	13435.8369	28622.7539	1.003406	0.027858576
RETNLB	151.504211	598.350891	1.933814	0.035001669
PROSER1	6482.34814	13705.3965	1.021346	0.02871665
SYNC	2773.26611	10097.8525	1.81962	0.001071791
OR5P3	224.484909	708.430054	1.654198	0.043075189
FABP1	677.149963	2610.29224	1.938302	0.002745164
GPATCH4	10521.2285	23171.1113	1.061796	0.021806203
PTRHD1	14744.8711	32428.6582	1.037804	0.023434993
MZB1	448.969818	1237.02783	1.465286	0.029967848
EXOSC6	1707.19385	3514.90283	1.033182	0.045353331
KCNH6	523.798096	1332.93835	1.350785	0.037147928
PRPS2	2683.65698	6039.09375	1.137354	0.022667473
NPY6R	152.428024	555.845093	1.819332	0.04725837
GIPC2	3416.23535	7399.2793	1.072972	0.027298505
RDH11	875.76825	2199.40283	1.329745	0.022859724
TRAPPC2L	4437.96484	9377.43457	1.029911	0.030386703
SLC27A1	710.406982	2610.29224	1.869833	0.00335685
ZIC2	1770.93652	4216.79346	1.240077	0.018326966
ENTPD7	287.303711	1324.21924	2.222632	0.004459913
SREK1	903.482483	1983.60413	1.139311	0.046830792
CBX2	314.094116	901.341003	1.532165	0.039075837
FAM13B	2548.78125	6030.37451	1.211148	0.016705578
PPM1B	2802.82788	5857.08154	1.030613	0.035706308
HERPUD2	3133.55054	6751.8833	1.069069	0.028739458
ASB16	820.339905	2511.11206	1.610293	0.007898513
RAB4A|SPHAR	505.321991	1281.7135	1.346234	0.039344627
SAA4|SAA2-SAA4	5522.51318	13412.2158	1.225632	0.011511652
E2F3	2365.86768	5241.29248	1.122727	0.02577517
LMAN2L	122.866226	611.429626	2.232085	0.036646321
NDUFB3	1751.5365	4031.51196	1.191971	0.022753332
BRCC3	4148.81348	8716.95898	1.024141	0.031839866
RLN2	106.237709	471.923401	2.040044	0.045330923
PRKAR1A	13598.4268	28957.3516	1.002215	0.02797894
NTF4	323.332153	924.22876	1.525904	0.0382559
BHLHE22	2105.35425	4820.59424	1.176198	0.021725634
LBR	8083.3042	19976.6367	1.244189	0.009619867
DPP8	3534.48242	7562.76318	1.054651	0.029277664
MMD	9782.18359	23213.6172	1.170583	0.013159151
CA13	793.5495	1767.80542	1.160423	0.048431173
HIST1H4B	1237.90027	2629.9104	1.086505	0.044955108
TMEM238	540.426636	1292.61243	1.26186	0.049051218
MAP3K12	1164.91956	2640.80933	1.180618	0.032695729

SUPT6H	1536.28967	3278.39624	1.086708	0.038741149
SETD8	1534.44214	3245.69946	1.074111	0.040847965
KPNA4	1770.0127	4346.49072	1.284198	0.015121312
HSPA13	1113.1864	2404.30249	1.11386	0.043663781
SYNGAP1	413.865173	1397.24207	1.75896	0.011786455
DNASE1	88.685394	6040.18359	5.914078	5.08075E-06
SMARCE1	7169.65967	17978.8652	1.273971	0.008584241
SERF1A|SERF 1B	2637.46655	5479.979	1.025762	0.037452627
VAMP5	5606.57959	12430.2227	1.091138	0.021486614
C8orf82	4554.36475	13725.0146	1.543759	0.002820645
LAMTOR3	1873.479	3788.46582	1.004004	0.048609525
TPTE2P5	1157.52917	2639.71948	1.189278	0.031249555
KIF5B	14385.5098	34050.418	1.137092	0.014629298
SFPQ	10487.0479	23999.4297	1.11444	0.016995633
TIMM10	1634.21313	4086.00659	1.312105	0.014184214
GTF3C6	1353.3761	2910.0127	1.100258	0.039900973
TTC32	1822.66956	3893.0957	1.083428	0.035342142
RPS26	13978.1113	37395.2969	1.301455	0.006686572
C9orf135	396.312866	1174.90405	1.574809	0.024314327
IPO7	3044.86523	6859.78271	1.133199	0.021795092
COX20	3781.13867	9216.12988	1.238502	0.012309486
AIMP1	873.920654	2016.3009	1.209779	0.036756191
HMGCS1	1869.78369	6083.7793	1.679187	0.00256368
LRRC61	2158.93506	5995.49805	1.447089	0.006396802
G3BP1	1724.74622	4227.69238	1.282571	0.015509374
EEF1A1	4853.67773	10369.2363	1.041301	0.028107163
AK4	3165.88379	6989.47998	1.102954	0.024574174
ARGLU1	6850.94678	15613.7988	1.132244	0.016911503
HPGD	269.751404	783.632629	1.55092	0.045729429
ACBD5	3606.53931	8016.15869	1.108329	0.022786798
C7orf55	4132.18506	8806.33008	1.044527	0.029064504
PPP1CB	3218.54077	6710.46729	1.021466	0.035169352
SLC2A5	5784.87451	12131.5918	1.009103	0.03115443
C9orf47	9264.85254	20621.8535	1.08844	0.019615937
STYX	5885.56934	13693.4082	1.162408	0.015217882
TRMT2B	2004.65942	4951.38135	1.286057	0.013780399
PWWP2A	3189.90283	7603.08936	1.21141	0.014967279
NA	1219.42419	3299.10425	1.431241	0.010863111
PTCHD4	1786.64111	4390.08643	1.284729	0.014985512
ZFX	460.979279	1143.29712	1.314269	0.048924778
ESCO2	376.912933	1205.42102	1.685884	0.017786564
DNAJA2	7089.28857	15854.665	1.104846	0.019094307
CHDH	4564.52637	11600.8145	1.292299	0.00894676

GATM	775.073364	2189.59375	1.497228	0.013361247
CYP20A1	2456.40063	5208.5957	1.058669	0.033529937
SLC12A2	2323.37256	4778.08838	1.018787	0.040934082
CTNS	614.331116	2003.22217	1.703706	0.007973934
RECQL	2107.2019	5089.79736	1.251607	0.015551018
LOX	2965.41797	6679.95068	1.134153	0.021956127
ZSCAN29	1511.34692	3138.89014	1.048252	0.045884606
ACLY	10713.3809	22858.3125	1.01685	0.026887206
RAPGEF5	1725.66992	4281.09717	1.299758	0.014381744
USP9X	678.07373	1647.91724	1.283799	0.035572872
NUP50	3181.58862	6570.96143	1.008607	0.037396487
ZMYND11	10253.3252	22467.041	1.05736	0.022354502
ZC3H4	4033.33789	8760.55469	1.072306	0.02581455
ZBTB2	2315.98218	5243.47217	1.154655	0.022635674
PYROXD1	2411.13428	5111.59521	1.05949	0.033701509
ERI2	1212.03369	2620.10132	1.111891	0.041235462
TIMM10B	1788.48877	4194.99561	1.218155	0.020029124
ROCK1|ROCK1P1	5324.81885	11295.6445	1.026782	0.029305985
SDPR	1743.22229	5046.20166	1.518337	0.005465119
SOS2	1563.08008	4020.61304	1.353658	0.012245144
DSC2	2286.42041	5167.17969	1.15286	0.022977863
C5orf24	4774.23047	11503.8145	1.2137	0.012709337
HCCS	994.015442	2221.20068	1.163478	0.039326739
ZBTB1	1111.33887	2913.28247	1.388066	0.014231594
HNMT	1932.60254	3946.50024	1.016797	0.045316577
SREK1IP1	1888.25989	4053.30981	1.089151	0.0337836
UTP15	1409.72827	3224.99146	1.188082	0.026924627
ARID1B	6988.59375	16641.5664	1.197218	0.012399352
RYK	5496.64697	14028.0049	1.298807	0.008184554
DCK	4349.27979	10371.416	1.203333	0.013759548
RAPH1	239.265808	3969.38818	4.041616	0.000140188
AP1G1	2261.47754	4939.39209	1.105358	0.028490273
SLC25A32	2213.4397	5175.89893	1.202947	0.018741934
QTRTD1	1608.34656	3385.20581	1.066011	0.04101146
DLX2	2974.65601	10465.1465	1.76862	0.001277351
RRM2	12785.4775	32185.6113	1.228823	0.009569701
EIF2S1	2203.27783	4628.77295	1.052028	0.036426552
SNX13	1574.16577	3268.58716	1.047043	0.044948943
CPSF6	1226.81458	3061.50781	1.315498	0.017643163
BRAP	659.597595	1638.10828	1.31494	0.03261552
C9orf64	2567.25732	6160.07178	1.23056	0.015248463
TMEM57	1382.93787	2929.63086	1.078487	0.042962212
DDX5	2259.62988	5111.59521	1.154929	0.022915723

ZNF367	1120.5769	2502.39282	1.160821	0.036010664
CHORDC1|LOC 727896	1037.43433	2392.31372	1.207634	0.031840663
TOP1P2|TOP1| TOP1P1	8888.86328	20257.8301	1.124613	0.016648963
TIMM8B	22116.8438	54688.6211	1.134694	0.014203204
SAA4	3370.96875	8510.96973	1.291978	0.010094456
NA	3144.63623	6710.46729	1.055249	0.030540951
ENTPD7	1787.56494	4172.10791	1.211086	0.027827621
NA	3181.58862	7144.24463	1.126669	0.022030663
TSNAXIP1	261.437164	804.340576	1.631754	0.037214931
TMX1	4836.12549	12538.1221	1.321786	0.007651076
NA	1939.99304	4768.2793	1.281095	0.014393281
GP1BB|SEPT5-GP1BB	12095.3945	32383.9727	1.315148	0.006390967
NA	3091.05542	7033.07568	1.146516	0.020386582
EME2	651.283386	1639.19812	1.33412	0.030792639
PTGES3	2296.58228	62372.3633	4.616188	1.20486E-05
CCDC69	2991.28442	6258.16211	1.029463	0.034933392
GMFB	10911.999	28438.5625	1.287901	0.007396296
GPR132	223.561096	764.014587	1.769947	0.03068766
MARCKS	14745.7939	32850.4453	1.054799	0.021615306

"a" refers to the group treated with siCtr RNA.

"b" refers to the group treated with siPCNAP1.

"Upreg^{gene}" refers to the genes upregulated by siPCNAP1.

Table S5-2, related to Figure S3. List of genes regulated by PCNAP1

Gene_Symbol	Normalized Intensity		log2 (Ratio)	P-value
Downreg[gene]	C[a]	T[b]	T/C	T/C
AMT	4934.04883	2394.49365	-1.066497	0.03252282
FUCA1	8589.5498	3472.39722	-1.351593	0.007561329
CXCL8	106923.18	15583.2812	-2.977358	1.62331E-05
EPCAM	57553.125	24422.3086	-1.421372	0.003621404
C4BPA	10523.0762	2114.39111	-2.352709	0.000162848
SERPINA6	2765.87573	948.20636	-1.550387	0.008067991
BIRC5	6609.8335	1747.09753	-1.944204	0.000866048
CCT5	68366.2734	38412.168	-1.08002	0.018059656
VPS72	2748.32349	1313.32031	-1.068671	0.045170069
PPP4C	3932.64307	578.732849	-2.781115	0.000153926
PRKAG1	11700.0059	5727.38428	-1.087415	0.021817017
CASP6	15153.1934	5987.86865	-1.389148	0.00523984
HDAC2	10642.2471	5307.77588	-1.059953	0.025266152
TMEM98	7098.52686	1390.70264	-2.37581	0.000206988
FKBP10	2972.80835	1237.02783	-1.268607	0.020558426
CCNYL1	1609.27039	677.913086	-1.245145	0.039123621
CCNL1	9473.63281	4531.77246	-1.113501	0.020617718
TSSC1	19339.8828	8177.4624	-1.303831	0.007258385
NUP37	31113.793	16081.3623	-1.051521	0.021883445
PIH1D1	41636.8672	18088.9434	-1.33337	0.005627913
7-Mar	3807.00537	1681.70398	-1.188276	0.023213718
MMADHC	42251.1992	16703.6914	-1.47386	0.002935509
FBXL5	3244.40723	946.026611	-1.785819	0.003032244
YIPF5	20180.5469	10216.6514	-1.051093	0.023130145
CYB5R4	1464.23279	459.934601	-1.669875	0.012663457
FBXO7	3128.00781	1399.42188	-1.165875	0.028916517
COPS4	1371.85217	298.630524	-2.181904	0.003963116
MPDU1	3233.32178	1261.00549	-1.363263	0.013521042
PCID2	13228.9043	6269.06104	-1.132966	0.017244445
RNF167	1870.70752	849.026184	-1.13701	0.047901422
PEX3	18923.2461	7174.76172	-1.457063	0.003649298
ATG4C	4610.7168	1908.40161	-1.287611	0.013844527
TM2D2	13886.6543	6161.16162	-1.225862	0.011189442
BLOC1S4	2377.8772	818.509155	-1.543337	0.00979046
NDUFV1	14165.6445	2652.7981	-2.452588	0.000102006
RHBDD2	1924.28833	427.237823	-2.172407	0.002166653
SLC25A38	4736.35449	1971.61536	-1.281135	0.01396366
OMA1	1869.78369	827.228333	-1.17432	0.042476844
FHL2	27333.5781	8532.76758	-1.769628	0.000873471
CYTH2	3371.89258	806.520386	-2.075187	0.001175379
STXBP3	542.274231	148.225372	-1.931753	0.036068302

NUP54	9067.1582	3441.88013	-1.443195	0.005657944
ANGPTL4	1501.18506	646.306213	-1.213315	0.046175189
LIPC	921.958557	311.709229	-1.549461	0.033895224
YIPF1	1509.49927	536.227051	-1.491795	0.02010629
ASGR1	47243.4492	23718.2383	-1.158191	0.012729442
TMEM128	6318.83447	2888.21484	-1.165899	0.019182516
AGTRAP	3225.93115	1605.4115	-1.013941	0.049968537
FAM210A	940.434692	178.742355	-2.416887	0.005448379
PARL	17819.2969	6236.36426	-1.563434	0.002325449
HAT1	28392.2598	12116.334	-1.326268	0.006107373
ZC3HC1	1353.3761	566.744019	-1.253039	0.045658555
ARHGAP5-AS1	2546.93359	1022.31903	-1.319182	0.019655269
PPTC7	1970.47864	891.531982	-1.141982	0.045096863
SH3BGRL	2577.41919	926.408508	-1.480749	0.011056551
PPAN	2314.13452	657.205139	-1.822977	0.004171659
DUSP11	6083.26367	1553.09668	-1.990495	0.0007926
TGOLN2	1208.3385	492.631348	-1.291388	0.046327196
ZCCHC9	5714.66504	2735.62988	-1.094556	0.027066778
KCTD14|NDUFC2-KCTD14	2694.74268	549.305786	-2.306682	0.000853185
ENOPH1	3596.37744	1160.73535	-1.636744	0.004526097
EID2	8632.04492	4347.58057	-1.037117	0.029641466
MFSD5	831.425598	170.023209	-2.322157	0.008331377
MRPL45	1978.79285	599.440796	-1.72601	0.006969413
MAD2L2	2086.87817	903.520813	-1.206852	0.034642894
CTH	1345.06177	253.944931	-2.396063	0.00262158
ERI1	2874.88477	525.328125	-2.465732	0.000517073
RSF1	1192.63379	414.159119	-1.519274	0.025488503
BCKDK	1184.31958	322.608154	-1.859933	0.011058006
RBM48	822.1875	202.719986	-2.032336	0.014549307
APMAP	4998.71533	1737.28845	-1.540481	0.004824144
TPGS2	9901.35449	5008.05566	-1.036686	0.028549729
BET1	16807.7305	7464.67285	-1.223651	0.010802291
FARSA	3028.23657	1487.70312	-1.031079	0.048679855
ACTR6	2366.7915	622.328552	-1.93505	0.002894385
DERA	59883.8906	31858.6445	-1.123921	0.014747894
RAD51AP1	6535.92871	1875.70483	-1.82637	0.001331242
IFT52	6708.68066	2674.59595	-1.362851	0.008133653
LRIF1	1907.65979	850.116089	-1.1639	0.043149259
PRSS23	2993.13208	1043.02698	-1.525837	0.008084187
FOS	8949.83398	2728.00073	-1.755907	0.001404194
RASD1	7720.24854	23.977633	NA	NA
TMEM37	12368.8408	3711.0835	-1.782377	0.0010729
ERGIC2	14492.6719	7477.75146	-1.012283	0.029378556

RIOK2	5947.46436	2759.60742	-1.141064	0.021893261
GMPPA	794.473328	277.922546	-1.503391	0.046180714
TMCO3	976.463135	353.125122	-1.455998	0.039163787
ZNF395	1879.02185	839.217163	-1.160525	0.044208948
SMIM3	8788.16797	3258.77832	-1.475819	0.004410054
COPS2	5949.31201	1464.81543	-2.04153	0.000681757
GTF2H3	2177.41113	762.924683	-1.516661	0.011843391
FN3KRP	7955.81885	3409.18335	-1.266037	0.011314258
WRAP73	2755.71387	1053.9259	-1.390448	0.014166337
TBC1D19	787.082886	138.416336	-2.575554	0.006231106
PITHD1	5757.16016	1613.04077	-1.855022	0.001331495
METTL18	1156.60535	289.911377	-1.975611	0.008719206
RFC4	19645.6621	7260.86328	-1.497234	0.003025203
CCNDBP1	4782.54443	1473.53455	-1.711257	0.002633653
DKK1	31843.5996	7746.95508	-2.146014	0.000201772
ZNF277	44332.5352	15970.1934	-1.614776	0.002583478
CHD6	2629.15234	1162.91516	-1.177783	0.031707715
MTERF3	7720.24854	3709.99365	-1.101162	0.023304883
GLMN	1760.77466	232.147079	-2.924133	0.000545329
SRP54	28328.5176	13898.3076	-1.120233	0.016014714
TSPAN31	2170.94458	526.41803	-2.050464	0.002415077
MAT2B	4510.9458	622.328552	-2.876157	0.000103338
TTF1	1647.14648	633.227478	-1.377707	0.025414398
OXSM	1273.92871	503.530273	-1.336471	0.038512472
ZNF622	11518.9395	5608.58643	-1.095017	0.021171818
PIGU	4412.09814	1022.31903	-2.119753	0.000725149
CA9	10730.9326	4735.58252	-1.233063	0.011668596
HACL1	4590.39307	1552.00671	-1.57647	0.00450371
YIPF4	7141.9458	2522.01099	-1.537679	0.003819347
VAMP3	8253.28418	2856.60791	-1.572493	0.003051233
GAR1	2991.28442	1074.63391	-1.481281	0.009470672
WDR12	34729.5703	11599.7246	-1.705571	0.001088719
APEX1	85646.0703	25114.3906	-1.98106	0.000320484
VEGFA	939.510925	332.417175	-1.48597	0.038345352
OAZ2	3569.58716	1185.80298	-1.595255	0.005363987
PTPRN2	5008.87695	2394.49365	-1.088683	0.029504338
RHOG	540.426636	158.034393	-1.823601	0.043459442
ILF2	23573.6855	3203.19385	-2.9367	2.38942E-05
LRPAP1	5359.92334	1228.30872	-2.140072	0.00056601
NDUFA9	25279.957	13165.9004	-1.02554	0.025268069
ARL6IP1	12296.7842	4357.38965	-1.544377	0.002853515
PIAS1	2106.27808	948.20636	-1.149864	0.041548491
CPE	7377.51611	2490.40405	-1.60288	0.002880483
FGG	12377.1553	6289.76904	-1.034512	0.027287168

SCP2	93767.25	34018.8125	-1.707902	0.000967606
PRSS8	749.206848	203.809875	-1.891907	0.022462901
PMM1	2621.76196	1193.43213	-1.136527	0.036610954
LMAN2	52217.2227	12829.123	-2.189349	0.000158571
PNRC1	6055.5498	2663.69702	-1.217654	0.01575307
AMBP	110503.852	56254.7969	-1.280261	0.006777674
ALDH1A1	51024.5859	19403.3535	-1.550218	0.002035448
IGF2R	3237.01685	1375.44421	-1.240455	0.021335157
LITAF	18825.3223	4086.00659	-2.246051	0.000168848
F2	5353.45703	1700.23218	-1.672431	0.002761738
FBXO8	3433.7876	1540.01794	-1.164337	0.027259572
RHOA	55048.6875	24003.7891	-1.376634	0.004476127
SERPINI1	5496.64697	1334.02832	-2.058895	0.000694539
QPCT	39032.6602	8566.55371	-2.320858	0.000107721
CCNG1	9916.13574	2501.30298	-2.028271	0.000483499
PSMC5	35227.5	18874.7559	-1.013152	0.025898507
POLR2C	6166.40625	2383.59473	-1.401579	0.007269685
MREG	892.39679	280.102356	-1.656895	0.027766809
SMNDC1	5630.59863	2554.70776	-1.169514	0.019967441
GADD45A	46005.5469	15582.1914	-1.707952	0.001033843
AUH	6164.55859	2565.60669	-1.297033	0.011204004
PHLDA1	47903.9688	15602.8994	-1.768076	0.00079853
PRIM1	10814.0752	4066.38843	-1.461698	0.004273465
VRK2	1406.95679	525.328125	-1.419355	0.027067836
PPP1R11	2885.04663	894.801636	-1.696511	0.004686977
CRYZ	65508.0195	10961.0479	-2.767364	2.79278E-05
MCL1	17604.9746	4421.69336	-2.031068	0.000362444
SNRPD1	8732.74023	4476.18799	-1.012322	0.032898556
CLP1	8471.30273	3645.68994	-1.261667	0.011216391
PAX6	16093.6279	6430.36523	-1.373384	0.005531385
PGRMC1	30472.6719	15680.2822	-1.056247	0.021449061
15-Sep	143390.422	53685.918	-1.736473	0.00083721
DUSP14	6309.59619	1616.31042	-1.987175	0.000776418
EXOSC9	3334.94043	1055.01587	-1.666381	0.004412128
TCEAL1	9990.04004	3958.48926	-1.384517	0.006155846
CPVL	8887.93945	2631.00024	-1.797	0.001203936
TRUB2	4130.3374	1918.21057	-1.1192	0.028932897
STXBP6	16608.1875	5976.96973	-1.520943	0.002853655
PHACTR2	3490.13989	1356.91602	-1.36938	0.012388689
TTC5	3453.1875	1538.9281	-1.173544	0.026220217
FNDC3A	2945.09424	1432.11865	-1.045153	0.047040146
CCDC115	4693.85938	2037.00891	-1.221689	0.017831635
KIAA1324	1226.81458	504.620178	-1.278601	0.048096485
TMEM45A	6137.76855	1259.91565	-2.303406	0.000292983

ENC1	15641.8867	7217.26758	-1.170313	0.014004644
GMDS	5948.38818	2101.3125	-1.526848	0.004484135
ATP6V0D1	2486.88623	861.015015	-1.535217	0.009559072
CRYL1	7106.84082	3118.18213	-1.229043	0.013912309
HOXA10	4700.32568	1238.1178	-1.935118	0.001222553
DDIT4	6319.7583	2879.49561	-1.170392	0.018820738
LAPTM4A	70382.9453	22155.332	-1.86198	0.000570061
HDAC8	1635.13696	556.934998	-1.553037	0.015145057
ALDH18A1	3560.34912	1496.42224	-1.258121	0.018585118
MPZL2	6084.1875	2425.0105	-1.357424	0.00878668
TRIP4	3462.42554	687.722107	-2.345996	0.000522292
DNASE2	1598.18469	267.023621	-2.570096	0.001282199
FUBP1	2501.66724	792.351746	-1.665469	0.006168399
GPC3	5822.75049	2435.90942	-1.286343	0.01208464
NOP58	79877.8281	43970.6172	-1.131777	0.013994608
FER	4112.78516	1712.22095	-1.274862	0.015720313
UGT2A3	42315.8672	19220.252	-1.275047	0.007388491
TAF2	2051.77344	935.127686	-1.131619	0.045136459
RAB3B	3128.93164	1367.81494	-1.199037	0.025544776
RND1	1462.38525	308.439545	-2.229345	0.003146535
DIMT1	1960.31677	708.430054	-1.470237	0.015585485
GALK2	929.348999	321.51825	-1.517311	0.036138788
SH3GLB1	4863.83936	2104.58228	-1.227952	0.017020633
RFC2	16773.5488	6516.4668	-1.412932	0.004583573
CHEK2	5595.49414	2187.41406	-1.37989	0.008429658
SERINC1	1977.86902	764.014587	-1.37322	0.021041736
TANK	12149.8994	3169.40698	-1.983005	0.000507001
OXA1L	13208.5811	3795.00537	-1.842954	0.000823007
GBAS	6544.24316	2078.4248	-1.683054	0.002276583
PAF1	668.835693	201.630096	-1.74638	0.03581417
TOM1L1	8579.3877	2739.9895	-1.688013	0.001877004
UBXN8	6400.12939	2976.49609	-1.141661	0.021125957
RTN4IP1	7719.32471	3234.80054	-1.29714	0.010017638
STX6	513.63623	146.045578	-1.877663	0.042965718
CDC37	3072.57935	1238.1178	-1.315352	0.016849572
TUBAL3	2150.62085	913.329834	-1.235309	0.030587889
SRSF5	45381.9805	23385.8203	-1.115718	0.02444672
FAT1	3181.58862	1130.21838	-1.497226	0.008420971
PDGFRL	749.206848	257.2146	-1.537749	0.046362665
DUSP1	31645.9043	13562.6211	-1.329453	0.005924436
CSTF1	13011.8105	4302.89502	-1.642049	0.001852287
UQCRC2	25597.7461	3827.70215	-2.807148	3.12175E-05
TACO1	4483.23145	1530.20886	-1.56192	0.004855081
MGAT4A	1653.61304	709.519958	-1.21862	0.041344583

CTNNBL1	2974.65601	1088.80249	-1.454019	0.010498778
DEPDC7	2341.84863	863.194763	-1.443148	0.013921201
GRN	1454.07092	482.822327	-1.589414	0.016040653
PEX2	2828.69458	923.138855	-1.622229	0.006156132
IFNGR1	67406.4453	21592.9473	-1.831912	0.000590781
MED4	4756.67822	2219.021	-1.120205	0.026655007
TMEM9	7757.20068	3364.4978	-1.248024	0.012342281
ARL4D	2067.47827	419.608582	-2.303264	0.001363221
ATP6AP2	7053.26025	3614.08325	-1.006783	0.036025278
PMPCB	14150.8633	6463.06201	-1.184906	0.013407693
EIF3I	56196.0547	13693.4082	-2.205868	0.00014832
CNN3	13038.6006	4088.18628	-1.718201	0.001354896
SLC38A5	1019.88202	364.024048	-1.475216	0.035275184
WRB	8915.65332	3333.98071	-1.464187	0.004599951
BPGM	2955.2561	794.531555	-1.905286	0.002318768
SCAMP3	2441.61987	871.91394	-1.489806	0.011366005
HMOX1	13001.6484	4712.69482	-1.511319	0.003352619
AGPAT2	4809.33496	1227.21887	-1.98158	0.001023135
PSMC4	5105.87695	2500.21313	-1.055831	0.033420473
RAB1A	101081.023	47898.5898	-1.365288	0.004543283
HMGN4	1889.18372	406.529846	-2.215103	0.001991899
RMND1	15143.0312	5079.98828	-1.62028	0.001919347
ANKRA2	9185.40527	4564.46924	-1.058123	0.026546303
PEF1	2232.8396	870.824036	-1.360178	0.019356679
TNF	1762.62219	656.115234	-1.425024	0.020315474
SERPINH1	6164.55859	2391.22388	-1.396617	0.007420558
CD68	1579.70862	598.350891	-1.399046	0.024998525
HES1	2458.24829	999.431335	-1.300173	0.021817155
NARF	11320.3213	4088.18628	-1.519028	0.003283266
NSMCE4A	20541.7539	8728.94824	-1.302511	0.007212088
COPG2	911.796692	342.226196	-1.402523	0.04865795
NRAS	2204.20166	709.519958	-1.640457	0.007840305
NAAA	577.378845	170.023209	-1.800817	0.040719368
DSN1	1058.68188	256.124725	-2.036222	0.008963224
RNF146	1328.43335	547.125977	-1.277048	0.043463703
TMA16	2195.88721	816.329407	-1.430141	0.015605955
FBXO5	2051.77344	750.935852	-1.452421	0.015626049
SERINC3	13319.4375	6127.375	-1.174844	0.014243685
HNRNPH2|RPL36A-HNRNPH2	1544.604	643.036499	-1.262077	0.038744949
GLOD4	9799.73633	3470.21729	-1.544848	0.003788145
MSRB1	2722.45679	1290.43262	-1.079983	0.043645632
LMCD1	2623.60962	1099.70142	-1.256096	0.023881512
EIF2B3	5127.12451	1583.61365	-1.710572	0.002483842

C8orf4	14666.3467	3268.58716	-2.204211	0.000215391
DDX47	13786.8838	3386.29565	-2.066424	0.000354163
TOMM22	5703.57959	2726.91064	-1.09621	0.026905384
SLC35B3	5722.97949	2447.89819	-1.253922	0.013952233
SCOC	28689.7246	9787.2334	-1.649575	0.001431289
MED21	8390.00781	1077.90356	-2.986752	4.0268E-05
ZNF556	2584.80981	481.732422	-2.435567	0.000665992
TRAPPC3	9248.22363	4336.68164	-1.141332	0.018381352
OPA3	812.949463	265.933746	-1.603208	0.035890464
RDH14|NT5C1B-RDH14	5766.39844	1581.43384	-1.88574	0.001192689
TM2D3	10633.0088	4485.99707	-1.296513	0.008837934
NUBPL	5141.90527	2457.70728	-1.090454	0.028925354
PPM1K	1338.59521	514.429199	-1.377375	0.032388009
C11orf49	1628.67029	481.732422	-1.7574	0.008588346
SLC36A4	2394.50562	580.912659	-2.051925	0.002036189
SIL1	1399.56641	406.529846	-1.777787	0.010172688
NAA35	3867.97656	1746.00757	-1.157616	0.025888104
RBM3	21809.2168	8619.95898	-1.411541	0.004332881
HABP4	5749.76953	2814.10205	-1.063571	0.030699445
PROSC	6227.37744	881.722961	-2.844054	7.3511E-05
PLEKHB2	22252.6426	8165.47363	-1.519413	0.002662775
TMEM243	12159.1367	4593.89648	-1.45293	0.004245258
YIPF2	4057.35669	1950.90735	-1.069362	0.037571095
IP6K2	9565.08887	2456.61743	-2.001174	0.000543758
ADD3	15433.1064	5920.29541	-1.430965	0.004328927
AHSG	8381.69336	2403.21265	-1.839532	0.00106352
GJB1	14475.1191	1938.91858	-2.926549	3.06588E-05
CAPZB	69724.2734	23980.9023	-1.740115	0.00086251
WIBG	7177.97412	3514.90283	-1.072184	0.027102033
CDK1	21310.3613	5586.78857	-1.990943	0.000392918
FIP1L1	3696.14868	643.036499	-2.538306	0.000292393
ZHX1-C8orf76|C8orf76	4899.86816	2480.59497	-1.006272	0.041622747
ADIPOR2	4304.93701	1821.21021	-1.252845	0.016627457
AMN1	2586.65723	1044.11694	-1.311088	0.019904215
MRPL10	7685.14355	2296.40332	-1.777235	0.001422715
RALB	2377.8772	839.217163	-1.506798	0.01105536
DHRS1	4500.78369	577.642944	-2.980764	0.046516683
TOP1MT	1562.15625	329.147491	-2.233955	0.002731875
AS3MT|C10orf32-ASMT	4438.88867	1571.62488	-1.509003	0.005985338
TM4SF1	20360.6875	5597.6875	-1.918818	0.000522941
BPHL	5803.35059	2672.41602	-1.150329	0.021307958

LIN52	2349.23926	924.22876	-1.34787	0.019182295
C2orf15	2386.19141	754.205505	-1.668125	0.006488077
SPTSSA	36385.0312	19643.1309	-1.009286	0.026299752
WDR66	4148.81348	1355.82617	-1.621948	0.004157097
UFM1	523.798096	137.326447	-2.004999	0.034034878
UFSP2	1328.43335	501.350494	-1.403608	0.030355221
CCT4	55401.582	28871.25	-1.134673	0.014078243
NOP56	7168.73584	2403.21265	-1.611405	0.002834332
CMTM6	6012.13086	1313.32031	-2.213438	0.000391066
TCEAL8	4619.95459	2337.81909	-1.003435	0.043232877
CAT	3136.32202	862.104858	-1.872396	0.002382847
PAIP2	17749.0879	6872.86133	-1.420259	0.0043712
CDK8	1445.75671	624.508362	-1.208364	0.048646279
EFNA1	1157.52917	428.327698	-1.428638	0.0336693
IGFBP1	64595.3008	29107.7559	-1.359579	0.004788298
ATP5B	155859.031	84933.1328	-1.233951	0.008371862
IER3IP1	5920.67383	3017.91211	-1.007112	0.038341723
SGCE	5522.51318	2616.83154	-1.106953	0.026120611
STX5	3885.52881	1366.7251	-1.515128	0.00654768
CXCR4	1672.08923	696.441223	-1.261708	0.035773106
RWDD2A	3452.26367	1606.50134	-1.111581	0.033202421
NR0B2	24748.7676	11523.4326	-1.189892	0.011766219
SCPEP1	1372.776	579.822754	-1.240666	0.046617597
ASB8	4167.28955	1130.21838	-1.889714	0.001616751
INTS12	1112.2627	343.316101	-1.681387	0.018684603
ARPC1B	106642.336	58626.4023	-1.171589	0.011428859
AFMID	1562.15625	417.428772	-1.900272	0.006165421
CLK1	7948.42822	2273.51562	-1.840564	0.001097
VCAN	1716.43188	720.418884	-1.250696	0.036054011
UBXN6	1969.55481	750.935852	-1.392276	0.019871978
OARD1	12025.1855	5901.76709	-1.08372	0.022033373
UTP18	9918.90723	3084.39551	-1.731161	0.002117423
DTD1	44276.1836	21779.3203	-1.174892	0.01182046
TMEM237	2097.96387	461.024475	-2.19231	0.001744265
AGT	14212.7578	5209.68555	-1.495339	0.003346838
CHEK1	6735.4707	2187.41406	-1.6532	0.002504821
UCHL5	4997.7915	2294.22339	-1.145936	0.023392428
TMEM161A	2847.17065	1228.30872	-1.215945	0.025809584
DARS	39594.332	13112.4951	-1.729598	0.000963307
SAE1	23870.2285	3611.90332	-2.783866	3.38164E-05
LTV1	6020.44482	1809.22131	-1.756475	0.001836393
DHRS7	34774.8359	18464.957	-1.022864	0.031349748
PICALM	2829.61841	970.004211	-1.550284	0.007877951
ASB3|GPR75-	3406.99731	1581.43384	-1.114921	0.033058077

ASB3				
PRKAA1	3064.26514	785.812439	-1.974101	0.001787118
MRPL19	44818.457	21002.2266	-1.243126	0.008543731
EIF3M	16382.7793	5899.5874	-1.520075	0.002875917
HEXB	10669.9619	2685.49487	-2.034675	0.000452508
STK17B	14548.0996	3514.90283	-2.088825	0.000319482
ALKBH1	3571.43481	1064.82483	-1.752483	0.003043247
HNRNPA0	4060.12817	473.013306	-3.119697	7.64322E-05
ZNF207	15052.498	5079.98828	-1.611828	0.001993166
HELZ	1798.65063	817.419312	-1.134724	0.0498969
ANKMY2	1889.18372	816.329407	-1.208896	0.037594393
ASCC1	4194.08008	814.149597	-2.378741	0.000367434
CDR2	867.454041	307.34964	-1.482529	0.043035332
HGD	11299.9971	4379.1875	-1.41809	0.005073165
ANKRD13A	1896.5741	668.104004	-1.506515	0.014436597
LPAR6	8848.21582	2737.80957	-1.734165	0.001538225
MRPS30	3488.29224	1186.89282	-1.560435	0.006131237
LRMP	1019.88202	374.922974	-1.433787	0.039423373
ARPC5	6851.87061	1777.6145	-1.9724	0.000762719
CTSC	8851.91113	2210.30176	-2.037571	0.000503811
TCP11L2	755.673462	265.933746	-1.499247	0.04995155
ELP4	1464.23279	330.237396	-2.135282	0.00388499
METAP2	19446.1211	8177.4624	-1.312167	0.006978564
GPCPD1	1671.16541	547.125977	-1.610434	0.012421705
ANXA5	39024.3438	15678.1016	-1.443175	0.00340978
MRS2P2	1535.36584	566.744019	-1.4363	0.023212778
FAM114A1	3381.13062	1626.11938	-1.063912	0.040294923
SH3YL1	1092.86267	289.911377	-1.893269	0.011644482
DPYSL3	1796.8031	438.136749	-2.036223	0.003411998
COMMD10	3010.68433	1389.61279	-1.120358	0.0351546
TMEM177	1212.03369	286.641693	-2.060483	0.006735293
METTL14	2142.30664	515.519104	-2.061239	0.002719681
ERP27	14846.4893	3882.19678	-1.975204	0.000475444
PON2	30522.5566	13541.9131	-1.275202	0.007666891
CNOT7	4375.146	1918.21057	-1.203059	0.020053811
ECHDC2	2548.78125	1021.22919	-1.321808	0.019422783
LCOR	1330.28088	538.40686	-1.302336	0.04037714
SFXN4	6318.83447	2672.41602	-1.275762	0.0121097
CCT2	21780.5781	6527.36572	-1.804538	0.000802179
SLTM	6111.90186	2091.50342	-1.573438	0.003660169
SLC38A6	1310.88098	514.429199	-1.346986	0.036192697
WDR45B	640.197693	191.82106	-1.759394	0.037404906
DNAJC22	3696.14868	815.239502	-2.192989	0.000726687
SMIM14	26274.8965	8039.04639	-1.794511	0.000796204

TIMMDC1	3634.25366	1259.91565	-1.534498	0.006478272
TDP2	17105.1953	2651.70825	-2.718935	6.60252E-05
TMEM156	2050.84985	719.328979	-1.514519	0.012801224
PDIA6	4256.89893	762.924683	-2.495397	0.000266163
C7orf25	2069.32593	407.619751	-2.345286	0.001232287
SEC22B	1790.33643	494.811157	-1.856675	0.005554139
CPB2	2424.06738	449.035675	-2.442083	0.00073491
REV3L	1778.3269	674.643372	-1.397731	0.021891488
CLRN3	6716.0708	1918.21057	-1.834743	0.001264393
SPG21	1292.40491	502.440399	-1.360525	0.0354033
CCNE1	4385.30811	1831.01917	-1.272292	0.015208643
ACTR10	3398.68286	1044.11694	-1.709101	0.003728544
PLRG1	3344.17847	1270.81458	-1.401186	0.011414046
MAEA	503.474365	127.51741	-2.067544	0.033369593
DCP1B	495.160126	103.53978	-2.387533	0.021812398
PRKAA2	957.063232	232.147079	-2.042213	0.010689063
FEZ2	7882.83838	3958.48926	-1.038795	0.030278618
CHIC2	1091.93896	409.79953	-1.406017	0.038914196
TSPAN3	4789.93506	1625.02954	-1.57331	0.004400332
GLO1	57895.8594	16187.082	-2.004694	0.000304614
LRRC75A-AS1	49188.9844	21833.8145	-1.333039	0.005544299
SUCLG1	10830.7041	4391.17627	-1.353971	0.006818552
DAP3	8952.60547	4271.28809	-1.115667	0.02079436
FAM118B	2830.54224	871.91394	-1.706667	0.004635627
SMPDL3A	5515.12305	1572.71472	-1.827991	0.001521828
WDFY2	1834.67908	568.923828	-1.690489	0.008620525
TRMT11	6555.32861	1888.78345	-1.820908	0.001354925
BAAT	6139.61572	1237.02783	-2.330118	0.000271075
ARF1	13688.0361	1193.43213	-3.541999	1.0907E-05
SC5D	7919.79053	3979.19702	-1.038153	0.030316224
SLC25A3	174927.328	77590.5312	-1.534703	0.002026693
C11orf80	593.083557	125.337624	-2.330692	0.016464114
DUSP6	12524.04	6355.1626	-1.036541	0.026969342
DEGS1	1516.88977	397.81073	-1.925162	0.006078099
STARD10	5778.40771	2523.10083	-1.22532	0.015628804
DDOST	127718.977	59770.7891	-1.416882	0.003531011
EVA1A	4716.95459	1195.61194	-1.990379	0.001014368
PRPF18	5069.84814	726.958252	-2.823204	9.85207E-05
RAB28	2910.91333	589.631775	-2.316579	0.000750439
DYNC2LI1	8923.04395	2628.82056	-1.803926	0.001169937
PAK1IP1	832.349365	233.236969	-1.836415	0.02099807
AATF	3625.01538	1205.42102	-1.594152	0.005229418
CMAS	9782.18359	2942.70947	-1.778203	0.001224929
CFI	47614.8203	18657.8672	-1.497451	0.002601651

LAYN	1454.99475	362.934174	-1.993505	0.005509923
RPAP3	8363.21777	1853.90698	-2.203454	0.000303308
PTCD3	5541.91357	2142.72852	-1.394908	0.007980024
ASAH1	8825.12012	2933.99023	-1.632194	0.002308898
PDHX	10051.9355	3548.6897	-1.549928	0.003033624
CHRNB1	1092.86267	355.304932	-1.607625	0.023023795
MRPL42	24685.0254	12495.6162	-1.066422	0.020950779
TSEN2	2258.70605	1022.31903	-1.14256	0.040256675
DR1	2178.33496	527.507935	-2.052466	0.002364336
BAMBI	96488.7891	31037.9551	-1.871407	0.00048885
RMDN1	9820.05957	5039.6626	-1.015774	0.031368133
LRRC58	1861.46948	722.598633	-1.365462	0.023058742
ATP5SL	3182.51221	612.519531	-2.391144	0.000527676
CFLAR	2030.526	935.127686	-1.11631	0.047899243
MAP1LC3B	11302.7686	2362.88672	-2.298108	0.000183652
SNRPD3	3300.75952	1508.41101	-1.13659	0.031091046
SOX4	48221.7578	25211.3906	-1.106409	0.016260101
TSPAN13	9702.73633	2933.99023	-1.770549	0.001267481
CCT7	22187.9766	2329.1001	-3.297029	1.20762E-05
THUMPD2	2315.05835	676.823181	-1.780958	0.004738686
MBIP	11310.1592	2735.62988	-2.091801	0.000359503
GMPR2	13191.0283	5273.98926	-1.372876	0.005869939
CCNI	10657.9521	5058.19043	-1.130085	0.018489918
DAZAP2	29854.6445	12991.5176	-1.301852	0.006791025
LETMD1	8753.06348	1239.20764	-2.846831	5.21799E-05
PMP22	1427.28052	224.517838	-2.669672	0.001364412
TMEM41A	3109.53174	892.621887	-1.809048	0.002955428
BAG1	5595.49414	2609.20239	-1.130435	0.023552248
RTFDC1	11608.5488	2910.0127	-2.040963	0.000422283
SERPINB1	24829.1387	11265.1279	-1.227239	0.009880129
BCAP31	11646.4248	2285.50439	-2.38743	0.000137767
ARSE	1734.90808	462.11438	-1.909449	0.005055704
MIOS	911.796692	321.51825	-1.490179	0.039544147
CCDC92	776.921021	232.147079	-1.745481	0.030711182
CD9	18347.7148	8277.73242	-1.206309	0.01148798
PPARG	5234.28564	913.329834	-2.536485	0.000184739
ITGA2	5796.88379	538.40686	-3.455666	2.67952E-05
ABI2	2234.68726	655.02533	-1.776414	0.005046083
RNASET2	31989.5605	16199.0703	-1.084155	0.018720115
CAPNS1	9077.31934	3525.80176	-1.410354	0.005735268
RORA	2801.9043	1075.72375	-1.384684	0.014231688
RALY	8914.72949	4508.88477	-1.032007	0.030018818
TMEM9B	2114.59229	720.418884	-1.557339	0.010772898
ZFAND1	3380.20679	990.712158	-1.777955	0.002968457

FYN	983.853577	373.833099	-1.386698	0.046573877
FH	39204.4883	11731.6016	-1.878373	0.000525085
TIA1	1915.974	764.014587	-1.326462	0.025320206
WARS2	2702.13306	1237.02783	-1.129525	0.036710493
SS18	4448.12695	1455.00635	-1.622541	0.003894788
HNRNPLL	12104.6328	4650.5708	-1.429292	0.004712832
SRSF3	68394.9141	31588.3516	-1.33922	0.005248831
PAWR	2703.05688	1290.43262	-1.069466	0.045545451
PEX13	2503.51489	535.137146	-2.236285	0.001153616
AGPS	811.101807	277.922546	-1.532866	0.042061076
NUPL2	10957.2656	3567.21777	-1.668169	0.001791829
PAAF1	11798.8525	3860.39893	-1.65943	0.001794972
KDELC1	994.015442	352.035248	-1.48569	0.035558812
IFNGR2	3227.77881	733.497559	-2.150456	0.000988928
IVD	2142.30664	707.340149	-1.603027	0.009152011
ID1	192796.5	72076.7656	-1.778387	0.00069338
WBSCR22	16474.2363	7259.77295	-1.235095	0.010296316
MYH7B	630.035828	124.247734	-2.431612	0.012559077
SLC44A1	2177.41113	979.813232	-1.150758	0.040304586
UGP2	24944.6152	4065.29858	-2.681631	4.29628E-05
NAE1	36167.0117	19554.8496	-1.006032	0.026717307
BUB3	8697.63574	3828.79199	-1.230016	0.012740047
AP1S1	6183.03467	3073.49658	-1.044902	0.032209277
KIAA1671	1643.45117	567.833923	-1.532332	0.015990963
SLC2A3	11717.5576	5176.98877	-1.232132	0.011401123
SEC22A	3261.95972	979.813232	-1.742347	0.003490121
MPP1	8509.17871	3485.47583	-1.332494	0.008242451
VRK1	3842.10986	1538.9281	-1.32875	0.013395115
NUCB2	11068.1221	4928.49316	-1.221196	0.012180713
COX7A2L	52571.0391	11254.2285	-2.391933	0.000103492
MIPOL1	2052.69727	589.631775	-1.803834	0.005247567
HSPA14	4998.71533	2026.10999	-1.322029	0.011444537
CSTF2	2603.28589	483.912231	-2.439502	0.000651911
ASRGL1	5777.48389	2879.49561	-1.038159	0.041178282
C6orf89	1770.0127	719.328979	-1.297941	0.03011458
AP2M1	3852.27173	1314.41028	-1.558551	0.005618786
C3orf52	1191.70996	451.215454	-1.397804	0.03523989
ACTL6A	19774.9961	4844.57178	-2.078346	0.000293343
CCNC	13771.1787	2717.10181	-2.378899	0.000128478
METTL25	622.645386	202.719986	-1.63535	0.049284156
CLU	5777.48389	772.733704	-2.926483	6.89269E-05
VBP1	13093.1055	5199.87646	-1.382349	0.005641914
ZCCHC11	3119.6936	1531.29883	-1.032965	0.047481764
CHCHD7	4710.48779	1344.92725	-1.819735	0.001811699

TRIM13	2267.94409	675.733276	-1.753055	0.005311588
CLASP1	1799.57446	632.137573	-1.509462	0.015265012
POLG2	7079.12695	2436.99927	-1.573296	0.00332625
NECAP2	6571.95703	1961.80627	-1.770977	0.001626514
LPIN2	1807.88867	492.631348	-1.877417	0.005162897
LTF	2855.48486	1075.72375	-1.412231	0.012681035
WRAP53	469.293549	94.820641	-2.459107	0.022169594
KCNIP4	701.168884	188.551376	-1.916034	0.024065627
SORL1	1030.96765	343.316101	-1.572849	0.027309228
TALDO1	22142.7109	9948.53809	-1.230219	0.009928885
BRAP	2087.802	577.642944	-1.858527	0.004361262
PLTP	1744.14612	622.328552	-1.486106	0.01704558
LMBRD1	10976.665	3386.29565	-1.74515	0.001313643
TSPAN7	3289.67383	1088.80249	-1.600407	0.005638419
ALG8	5117.88623	1550.91687	-1.737808	0.002250923
DDX1	21095.1152	10216.6514	-1.118541	0.016811326
TSEN15	966.30127	299.720398	-1.671385	0.023805127
CDO1	876.692078	322.608154	-1.429559	0.047984876
RFC5	5775.63623	2890.39453	-1.032339	0.034877345
VRK3	396.312866	97.00042	-2.17373	0.043136556
PSMD11	10759.5703	2469.69604	-2.165393	0.00028887
KIAA0020	4222.71826	915.509583	-2.217041	0.00057162
C7orf49	891.472961	275.742767	-1.679293	0.026448689
KYNU	9059.76758	3028.81104	-1.62497	0.002344271
PHB2	23653.1328	5513.76562	-2.168236	0.000202285
TSG101	20001.3281	9421.03027	-1.153133	0.01575906
HMGCL	14138.8535	1637.01831	-3.134824	2.03482E-05
TBCE	966.30127	265.933746	-1.849106	0.015886523
MAPK8	1345.9856	525.328125	-1.355004	0.034294717
CYP20A1	1137.20544	406.529846	-1.476258	0.030470094
GPRC5C	1537.2135	503.530273	-1.609351	0.013988061
MLLT10	543.198059	148.225372	-1.934199	0.035807595
ASNS	72073.5078	40808.8398	-1.078503	0.018145863
TFG	28869.8672	9788.32324	-1.659202	0.001371782
JKAMP	15648.3535	5090.88721	-1.66352	0.001583859
NR2C2AP	3613.92993	913.329834	-1.994121	0.001358616
CHM	2520.14331	1020.13928	-1.306752	0.020704467
ANKRD1	920.110962	180.922134	-2.366267	0.02041485
SLC35A3	1436.51868	566.744019	-1.339639	0.033297863
UCK2	10037.1543	4940.48242	-1.075739	0.023941267
ASMTL	821.263733	233.236969	-1.817335	0.022405466
CERS5	1680.40344	566.744019	-1.567413	0.014001186
ABHD3	7017.23193	2811.92236	-1.358029	0.008103882
ENOSF1	1952.00244	611.429626	-1.677279	0.008223278

DFFA	31437.125	12280.9072	-1.465626	0.003172627
SYAP1	34531.875	13661.8008	-1.455146	0.003281208
NMD3	1454.99475	613.609436	-1.243119	0.043594375
CD40	3370.96875	1550.91687	-1.12738	0.03174955
ANKRD37	4330.80322	1930.19946	-1.179309	0.02217688
SAAL1	2920.15137	1022.31903	-1.519265	0.008488071
GNPDA2	1011.56775	192.91095	-2.402777	0.00475369
CRK	594.931213	123.157837	-2.36343	0.015613605
ENO2	9907.82129	3805.9043	-1.428712	0.005105278
RRP8	649.43573	115.528595	-2.592424	0.009388391
KCTD18	1040.20581	374.922974	-1.461864	0.035509441
GPS1	4247.66064	1063.73499	-2.006154	0.001080589
MOGAT3	3823.63379	1335.11816	-1.525301	0.006395776
ART4	352.893951	75.202576	-2.43203	0.03933163
DCDC2	5993.65479	2631.00024	-1.219985	0.015681528
GDE1	9891.19336	4594.98633	-1.156686	0.01680862
PLEKHJ1	11210.3877	4227.69238	-1.457253	0.004294397
ISY1	6264.32959	1678.43433	-1.922521	0.000976601
ROBO1	11040.4082	4184.09668	-1.450553	0.004446517
AZI2	11671.3672	5857.08154	-1.052355	0.025536964
METTL17	8535.04492	1118.22961	-2.958052	4.20855E-05
TGM2	2828.69458	1293.70227	-1.132155	0.035172537
L2HGDH	1056.83423	343.316101	-1.60811	0.024164466
CCDC77	2024.98315	527.507935	-1.94511	0.003573414
IMP3	16103.7891	7746.95508	-1.110417	0.018310158
STRAP	24151.0645	11633.5117	-1.138657	0.014999883
PAPSS1	13137.4482	5932.28418	-1.201124	0.012692823
TMEM55B	1138.12927	306.259766	-1.87518	0.011307857
SPSB3	622.645386	202.719986	-1.63535	0.049281638
IFRD1	12775.3154	3601.00439	-1.871042	0.000750035
TIMM21	8507.33105	3818.98291	-1.201402	0.014552662
TGDS	1853.15527	407.619751	-2.183025	0.002230173
PDZK1	13092.1816	3883.28662	-1.797569	0.000985788
GPN1	3055.95093	1044.11694	-1.554516	0.007153056
THOC3|LOC728554	16962.0059	3375.39673	-2.363153	0.000121908
KRT12	3200.0647	1055.01587	-1.606342	0.005683314
SLC3A2	8905.49121	3537.79077	-1.377607	0.006655386
LTA4H	34449.6562	5102.87598	-2.859214	2.52994E-05
TEAD4	704.864136	234.326859	-1.592756	0.044974633
IGF1R	3634.25366	1649.0072	-1.148874	0.027858542
CCNB1IP1	46901.6406	12345.2109	-2.081263	0.00023608
UQCRC1	8824.19629	1543.2876	-2.544212	0.000108077
CDIPT	1508.57556	374.922974	-2.000394	0.005078503

AXIN2	2214.36353	591.811584	-1.909979	0.003438619
MCCC2	1156.60535	277.922546	-2.039499	0.007549061
NUP88	2014.82129	764.014587	-1.400442	0.01887795
NDUFS2	19585.6152	5859.26172	-1.795298	0.000858849
LAP3	6202.43457	1043.02698	-2.592444	0.000132545
TFB1M	12486.1641	5544.28271	-1.224933	0.011559892
CYB5D2	876.692078	277.922546	-1.643519	0.029382298
SLC50A1	1755.23181	516.609009	-1.765107	0.007405363
HMG20B	1147.36731	276.832672	-2.03383	0.007771917
ADA	1445.75671	612.519531	-1.236441	0.044782832
PXN	1798.65063	764.014587	-1.233533	0.036367532
PMAIP1	2847.17065	764.014587	-1.908377	0.002413027
TNFRSF1A	730.730713	201.630096	-1.872739	0.024370672
CTSE	4276.29883	1920.39038	-1.168098	0.023363253
ERO1L	1915.05029	783.632629	-1.288675	0.028630309
YTHDF2	912.72052	136.236542	-2.811866	0.003146572
RNFT1	1173.23389	341.136322	-1.767849	0.013882388
USP18	2188.49683	839.217163	-1.384745	0.018196747
FPGT	1374.62366	461.024475	-1.574718	0.01942116
ANAPC7	2204.20166	943.846802	-1.223434	0.031147491
CLK2	686.388	115.528595	-2.671941	0.007446153
MAT2A	2439.77222	837.037354	-1.548392	0.00934939
UNG	2041.61169	667.01416	-1.617252	0.00982813
PPP2CB	7198.29785	1206.51086	-2.599755	0.000111951
WARS	967.225098	310.619324	-1.622584	0.026634596
CYP24A1	15282.5254	7045.06445	-1.17164	0.013991153
RPS6KB2	1716.43188	656.115234	-1.386125	0.023631265
APOL1	1257.30017	526.41803	-1.252998	0.049345769
PPID	9105.03418	4347.58057	-1.114968	0.020746222
RNF170	3073.50317	1152.01624	-1.419068	0.011537605
PPP2R5E	2693.81885	852.295837	-1.667648	0.005597096
ATF4	1254.52881	386.911804	-1.688336	0.015235533
CENPH	3416.23535	1627.20935	-1.077973	0.037968792
NADK	884.08252	320.428375	-1.451025	0.045147277
LYZ	660.521423	158.034393	-2.111943	0.018856887
C1GALT1	3472.5874	655.02533	-2.420815	0.000428714
PSMC3	668.835693	180.922134	-1.911987	0.027454551
PIGK	4708.64014	1561.8158	-1.604931	0.003959729
CAP2	885.006348	190.731171	-2.229874	0.008638588
SERPINA1	38472.832	20687.248	-1.025895	0.024215046
ZNF426	448.969818	115.528595	-2.061996	0.040588077
GSTM4	1546.45154	341.136322	-2.169036	0.003254328
PLSCR1	1145.51965	361.844269	-1.650365	0.019284889
FGA	9828.37402	4713.78467	-1.111361	0.020579342

SEC16A	858.215942	265.933746	-1.680314	0.02804211
AP3S1	11915.252	3137.80029	-1.969862	0.00053722
MORF4L2	6029.68311	560.204712	-3.456214	2.54664E-05
MICU2	15043.2598	4710.51514	-1.717543	0.001286089
IK	7429.24951	2380.32495	-1.677036	0.002139186
GTF2H2|GTF2H2C|GTF2H2C_2	31572.9238	12809.5049	-1.410165	0.004082518
TWF2	13506.9707	4227.69238	-1.719921	0.001326398
TULP3	3479.97803	1690.4231	-1.050248	0.041722879
XBP1	11916.1758	2383.59473	-2.360251	0.000147302
MYH3	766.759155	265.933746	-1.51997	0.046690762
NASP	50952.5312	24100.791	-1.252416	0.008086486
CRTAP	2133.99219	675.733276	-1.663523	0.007606721
EXOSC2	2358.47729	1120.40942	-1.072139	0.049656916
POLR2D	795.397156	256.124725	-1.629342	0.03499819
PPP2R2A	2106.27808	481.732422	-2.134483	0.002009941
DNAJB14	1970.47864	601.620605	-1.71456	0.007256988
SPP1	7731.33398	3505.09399	-1.184562	0.016248686
LDHA	188419.516	115253.938	-1.084933	0.017108388
HPD	4699.40186	1852.81702	-1.357511	0.010348836
NOB1	5451.38037	2145.99805	-1.368473	0.00897564
RPS10-NUDT3|NUDT3	8045.42822	3861.48877	-1.104094	0.022676149
RUVBL2	2902.59912	1344.92725	-1.114022	0.041343492
WIPI1	8326.26562	3632.61133	-1.241609	0.012321615
SLC25A20	1755.23181	634.317383	-1.467785	0.017893849
FUCA2	15208.6211	7791.64062	-1.021607	0.027745614
CHORDC1|LOC727896	1581.55615	655.02533	-1.269661	0.036955394
TUBE1	1084.54846	299.720398	-1.835657	0.01348915
SNAI1	7912.3999	3872.3877	-1.075729	0.025786357
SRSF9	44386.1172	11965.9287	-2.042682	0.000274224
PCNA	91995.3906	46776.	-1.258131	0.007590317
SUMO1|SUMO1P3	29241.2383	9903.85254	-1.662453	0.001349458
ING4	1131.6626	428.327698	-1.395811	0.037778314
TSC22D1	5559.46582	1529.11902	-1.880075	0.001256582
EIF3L	11083.8271	4670.18896	-1.299178	0.008610619
SNX7	5035.66748	1800.5022	-1.500126	0.005611591
GRPEL1	3380.20679	1570.53491	-1.113349	0.033423405
RNF130	5749.76953	1906.2218	-1.614643	0.003256341
KPNA2	10216.373	3323.08179	-1.667612	0.001855366
CSMD2	2485.9624	926.408508	-1.427617	0.01378083

VAPA	28328.5176	13455.8115	-1.167989	0.012798023
SFN	3779.29102	1735.10864	-1.132856	0.028917043
GNB2	2240.22998	815.239502	-1.461497	0.013747528
RPL4	90158.8672	36351.1797	-1.562251	0.001841298
HIBCH	4574.68848	1604.32153	-1.523917	0.005515796
LOC93622	1021.72968	291.001282	-1.792212	0.016466279
YARS	3037.47485	1075.72375	-1.502084	0.008663046
KLF6	2657.79053	960.19519	-1.473521	0.010976381
COTL1	2032.37366	794.531555	-1.355921	0.021629997
ARPC1A	36611.3633	11222.6221	-1.835612	0.000630305
RNF34	8154.43701	3470.21729	-1.276628	0.010680798
UBE2I	52687.4375	14641.6143	-2.008064	0.000304362
TIFA	7700.84863	3591.19531	-1.144066	0.019385414
GNL3	14538.8623	4197.17578	-1.83405	0.000818297
NDRG1	13924.5303	7174.76172	-1.014733	0.029099738
RBPJ	38173.5195	13423.1143	-1.637909	0.001433376
RSU1	1825.44104	321.51825	-2.494088	0.001131899
RNPS1	9392.33789	2542.71899	-1.92584	0.000720284
NUTF2	12052.8994	6257.07227	-1.004452	0.031391181
UBE2V2	874.844482	319.33847	-1.440863	0.046882015
EWSR1	2376.95337	827.228333	-1.52727	0.010331624
MORF4L1	32813.5977	14212.1963	-1.317053	0.006246113
TMEM230	106516.703	59166.9883	-1.157456	0.012233279
RPL15	19992.0898	9072.26465	-1.206224	0.011301206
PIGC	7701.77197	1570.53491	-2.320594	0.000226287
VDAC1	75871.2812	36092.875	-1.316726	0.005800213
EIF3E	108866.867	66551.0078	-1.030934	0.022446172
NT5C3A	20406.8789	9830.8291	-1.122961	0.016558671
FBXO25	10884.2842	4390.08643	-1.361528	0.006584501
PIH1D2	3534.48242	1690.4231	-1.072849	0.037938055
CS	1210.18616	427.237823	-1.496834	0.026506886
METTL2B	6156.24463	1982.51428	-1.659807	0.006605715
PSMD10	22522.3945	11350.1396	-1.068134	0.021037923
CLN3	1561.23242	513.339294	-1.603925	0.01390513
SMS	17216.0527	5826.56494	-1.607579	0.001944045
TAF1A	2132.14478	764.014587	-1.483677	0.013510755
EIF4A2	132230.844	33500.0234	-2.24212	0.000121236
HSP90B1|HSP90B2P	12594.25	5058.19043	-1.366591	0.006125065
CLDN2	569.064636	147.135468	-2.012934	0.0291028
IMP3	8282.84668	2707.29272	-1.653663	0.002195239
MORF4L1	18924.1699	6764.96191	-1.540147	0.002530174
SFN	4195.00391	1606.50134	-1.394968	0.009703705
PCBP1	33500.9062	15916.7881	-1.182274	0.011729825

PIH1D1	8010.32324	2568.87646	-1.679086	0.002024752
PRRC1	1237.90027	514.429199	-1.263781	0.048688032
ZPR1	3505.84448	1216.31995	-1.532642	0.006748573
GOLGA7	25795.4395	13209.4961	-1.051379	0.022351703
IDH1	18515.8477	5155.19092	-1.890513	0.000601403
WDR13	630.959656	156.944504	-2.057302	0.02252472
DIP2B	1553.84204	610.339722	-1.34633	0.02984898
RNFT1	4075.83301	1560.72595	-1.394477	0.009940161
MTX2	10188.6582	5048.38135	-1.067109	0.024767803
TUBE1	1192.63379	158.034393	-2.955949	0.00133144
DHRS7	18752.3418	9030.84863	-1.115328	0.017402111
ZCCHC8	1543.68018	645.216309	-1.256307	0.039438974
ETFA	31184.9258	14169.6914	-1.241883	0.008936601
CBL	696.549866	211.439117	-1.732282	0.034558658
PDE6D	11718.4814	3742.69043	-1.694112	0.00156325
OSGEPL1	1434.67102	277.922546	-2.352441	0.002502694
PIGU	2375.10571	15.258493	NA	NA
ELF3	1797.72681	493.721252	-1.865927	0.005377567
BMP4	42371.293	19814.2441	-1.23559	0.008900348
TUBA4A	1962.16431	806.520386	-1.282317	0.028538743
C11orf58	23264.2109	7950.76514	-1.625063	0.001663385
SERPINI1	4972.84863	1108.42053	-2.177846	0.000530622
DNTTIP2	1166.76721	473.013306	-1.299399	0.047146484
CBR1	1997.26904	805.430481	-1.310373	0.025575336
TCEAL1	2746.47583	1087.71265	-1.339418	0.017056622
AKAP8L	3558.50146	1268.63477	-1.493983	0.007665928
GTF2H4	1599.10852	320.428375	-2.305617	0.002221468
NDUFAF1	3244.40723	1464.81543	-1.15357	0.029511068
FUS	18870.5898	7745.86523	-1.343772	0.00608182
SBDS|SBDSP1	35815.043	16263.374	-1.25491	0.008268085
LYRM7	1896.5741	732.407715	-1.373282	0.022183429
FAM133B|FAM133DP|FAM133CP	6483.27197	2478.41528	-1.420249	0.006536301
ARPC5	17874.7266	4261.479	-2.107026	0.000275216
YBX3P1|YBX3	2194.96362	384.732025	-2.513123	0.000754464
PERP	14140.7012	4150.31006	-1.810983	0.000905619
NEK2	2268.86792	859.92511	-1.402138	0.016532147
PRMT2	750.130615	246.315674	-1.605392	0.040096194
DCAF16	1111.33887	268.113525	-2.03619	0.00820597
CNOT2	3370.96875	1237.02783	-1.4514	0.009420442
GUSBP3	4131.26123	1897.50269	-1.134881	0.027200829
NUP35	3011.60815	1216.31995	-1.311654	0.017371303
RPL4	234136.828	127644.93	-1.257781	0.007402549

CDK11A|CDK11B	1426.35681	478.462769	-1.574601	0.01713782
WRB	6265.25342	1561.8158	-2.025922	0.00068547
UTP6	5559.46582	559.114807	-3.339361	3.37935E-05
DDX21	25742.7832	11462.3984	-1.257474	0.008534317
APOBEC3B|APOBEC3A|APOBEC3A_B	3968.67139	817.419312	-2.292542	0.000499463
TXNDC12	33022.375	15820.8779	-1.168702	0.012525157
NDRG2	2692.89502	795.62146	-1.767774	0.004042124
DRAM2	5264.77148	1669.71509	-1.673761	0.002792759
PIPOX	5551.15137	1452.82654	-1.951204	0.000984029
NRBP1	3950.19531	990.712158	-2.004572	0.001181387
SRP68	3325.70239	591.811584	-2.504964	0.000372085
RNASE4	15236.3359	3698.00488	-2.081299	0.000321352
SMN2|SMN1	17784.1934	5846.18262	-1.651624	0.001597456
CHEK2	6057.39697	2599.39331	-1.252676	0.013606431
GCDH	1273.00488	297.540619	-2.078417	0.005780394
TRAM2	1301.64294	536.227051	-1.276601	0.044489764
CMAS	5395.95215	2132.91943	-1.362059	0.00927191
PRCP	1806.96497	770.553894	-1.227852	0.036875282
GABARAPL1	1725.66992	635.407288	-1.440375	0.01986523
RBPJ	76097.6094	42834.9492	-1.09473	0.016749455
RNPS1	3507.69214	837.037354	-2.078294	0.001090187
GLUD2	1616.66089	689.901855	-1.226407	0.041258778
EIF2A	4755.75439	1724.20972	-1.477867	0.00639002
HDHD2	1590.79431	439.226624	-1.855176	0.006738706
GLTP	1744.14612	568.923828	-1.616067	0.01153524
PSRC1	1066.07239	353.125122	-1.580933	0.025509317
FGFR1OP2|LOC100335030	659.597595	104.629669	-2.781864	0.007073163
PEX13	2575.57178	1160.73535	-1.150191	0.035352264
PARP2	5053.21973	1368.90479	-1.897937	0.001290559
DCAF7	3560.34912	967.824463	-1.887645	0.001943769
PPP1R35	4141.42334	2090.41357	-1.001473	0.045890648
ZNF830	3450.41602	1421.21973	-1.28639	0.017092697
LYPLA2	884.08252	213.618912	-2.055924	0.012068903
SHISA5	1572.31812	494.811157	-1.667451	0.011498593
KLHDC2	6320.68213	2427.19043	-1.41236	0.006853841
CCT8	46609.7188	23546.0352	-1.147393	0.013414582
COX7A2L	52389.0508	21596.2168	-1.444729	0.003278611
CFDP1	5352.5332	2556.88745	-1.093658	0.028005498
RAPGEF6	614.331116	160.214188	-1.985703	0.026663212
KATNA1	3496.60645	1152.01624	-1.606633	0.005183647

YIPF3	2982.97021	1195.61194	-1.322364	0.016842408
OAS1	11608.5488	5067.99951	-1.248991	0.010603637
RPL4	190706.859	72844.0469	-1.748164	0.000788173
NBPF14	6012.13086	1226.12891	-2.31187	0.000291856
EEF1A1	204939.938	109789.219	-1.274508	0.006847864
HNRNPC	548.740906	127.51741	-2.191251	0.023542799
LUZP1	1346.90942	544.946167	-1.302893	0.039761975
HNRNPA1L2|HNRNPA1|HNRNPA1P10	38148.5781	20742.832	-1.009004	0.026233342
CES1	838.81604	235.416748	-1.833271	0.020905891
NBPF15|NBPF14|NBPF11	2340.9248	719.328979	-1.709073	0.005838482
HNRNPCL1|HNRNPCL3|HNRNPCL2	3597.30127	850.116089	-2.092305	0.00101169
GPKOW	2069.32593	566.744019	-1.873046	0.004239014
RBMX	2575.57178	535.137146	-2.278015	0.000988197
HNRNPCL3|HNRNPCL1|HNRNPCL2	5019.03906	1022.31903	-2.309729	0.000356577
TMEM69	6329.91992	1000.52124	-2.683278	0.000103939
FANCL	2278.10596	622.328552	-1.878884	0.003610437
SEC61A2	967.225098	329.147491	-1.54122	0.03228211
SLC25A5	739.96875	178.742355	-2.075753	0.016583409
ZCCHC7	2386.19141	935.127686	-1.353677	0.018516691
GOLGA8T|LOC645752|GOLGA6D|GOLGA6B|GOLGA6A	1084.54846	384.732025	-1.484997	0.031693932
HNRNPCL2|HNRNPCL3|HNRNPCL1	767.682922	170.023209	-2.208654	0.01194205
SMS	342.732086	63.21376	-2.701605	0.03189401
EIF3IP1	3037.47485	720.418884	-2.088289	0.001287337
PDHA1	23444.3535	6334.45459	-1.958899	0.000430535
CENPA	3561.27295	1131.30835	-1.659768	0.004213375
OMA1	2577.41919	1001.61108	-1.366607	0.016424639
TUSC2	1470.69946	483.912231	-1.602665	0.015167839
TBC1D7	2828.69458	958.015381	-1.567996	0.00740956
NINJ1	1581.55615	676.823181	-1.222242	0.04269515
TMEM237	8850.06348	4377.00781	-1.063864	0.026180819
HAT1	18335.7051	6235.27441	-1.607155	0.001911319
TRUB2	4520.18359	828.318237	-2.463167	0.000267054

HSPBAP1	1219.42419	204.899765	-2.5802	0.002282785
FAM49B	4061.052	634.317383	-2.695003	0.000176998
HOXA5	1400.49023	331.327271	-2.066004	0.004966136
PCCB	8444.5127	1670.80505	-2.366272	0.00018355
IMPDH2	17260.3945	2319.29102	-2.92116	2.82646E-05
LSM4	10244.0869	3850.58984	-1.460714	0.004388124
ID3	71722.4688	39640.4766	-1.109689	0.015634414
PROCR	7079.12695	2383.59473	-1.604644	0.002934752
TMCO1	9505.04199	4120.8833	-1.254352	0.011076734
CDK4	16654.3789	6852.15332	-1.331986	0.006608309
HDAC8	1906.73596	322.608154	-2.553413	0.000916981
UMAD1	1047.59619	234.326859	-2.156604	0.007101816
LINC00998	15651.124	7919.1582	-1.03888	0.025518911
TTC3	1298.87146	446.855865	-1.536511	0.02163408
RBM41	1807.88867	504.620178	-1.842589	0.00568863
ARL6IP6	5523.43701	935.127686	-2.581247	0.000155029
GMPS	739.96875	212.529022	-1.810503	0.02691336
MTIF2	2107.2019	787.992188	-1.421181	0.016798187
NIT1	1364.46179	450.125549	-1.59783	0.017089585
RNF170	741.816345	245.225784	-1.59628	0.04152761
SERPINF1	12648.7539	2351.98779	-2.463225	0.000105343
CKB	3761.73877	748.756042	-2.342977	0.000468216
MRPL44	7458.81104	2855.51807	-1.425324	0.005920448
AHSA1	14229.3867	7390.56006	-1.003168	0.030529676
RTCA	7910.55225	3127.99121	-1.380903	0.006929903
MSRB1	2675.34277	1031.03821	-1.379099	0.015173015
GDF15	108151.836	20462.7305	-2.605141	4.05045E-05
NUDT9	5895.73096	2349.80811	-1.355614	0.009016767
DDOST	79984.9844	47899.6797	-1.019356	0.023960326
MAGEF1	2296.58228	1032.12805	-1.153052	0.038292173
IMPA2	3397.75928	471.923401	-2.864035	0.000164056
PSMG2	3950.19531	1972.7052	-1.014716	0.044631194
TCEAL8	1770.93652	708.430054	-1.320823	0.027999898
C16orf87	12261.6797	4444.58105	-1.511994	0.003280198
HLA-F	648.511963	115.528595	-2.590379	0.009443814
TAF12	2168.1731	1010.33026	-1.099652	0.048104756
ARPC1B	4030.56641	395.630951	-3.361369	5.24307E-05
CENPL	793.5495	244.135895	-1.698987	0.030290196
FBXL4	1173.23389	395.630951	-1.559727	0.024330569
IDH3B	21010.125	5143.20215	-2.086116	0.00027957
ALG8	14321.7676	2135.09912	-2.77518	4.35204E-05
AURKB	1454.07092	578.732849	-1.326958	0.034054078
HNRNPA1P10|HNRNPA1L2	78003.4219	24034.3066	-1.902884	0.000438164

HACD3	6616.2998	2843.5293	-1.255763	0.012844972
CD99	4701.24951	2330.18994	-1.033771	0.037984364
SNX1	1129.81494	409.79953	-1.455538	0.032373685
RNPS1	2107.2019	214.708801	-3.307106	0.000202493
LRRFIP1	3200.98853	709.519958	-2.186622	0.00090231
NBEAL1	946.901367	265.933746	-1.820247	0.017525444
RSL1D1	32097.6465	12497.7959	-1.472296	0.003068345
KLF6	20388.4023	1831.01917	-3.508713	8.88504E-06
BCAP31	9058.84375	4045.68066	-1.210543	0.013661075
MALAT1	29475.8848	15087.3799	-1.061143	0.021035746
RNFT1	1120.5769	331.327271	-1.742121	0.015925016
CCZ1|CCZ1B	16237.7412	7866.84326	-1.100193	0.019156823
RTN3	2648.55225	622.328552	-2.100465	0.001521143
VDAC2	30876.375	14102.1172	-1.233392	0.009311509
ATXN7L3B	2222.67773	17.438278	NA	NA
NBPF15|NBPF14|NBPF11	2882.27539	923.138855	-1.649518	0.005495102
YKT6	1383.86169	267.023621	-2.360946	0.002648555
CLMN	1236.97644	277.922546	-2.137081	0.005352515
NA	415.712799	95.91053	-2.263606	0.035507146
DDX41	1438.36621	234.326859	-2.615835	0.001478547
GFPT1	776.921021	265.933746	-1.538705	0.044032324
GGNBP2	18444.7148	6191.67871	-1.626079	0.00176025
RAF1	1256.37646	461.024475	-1.444065	0.029089978
TRA2B	22125.1582	6558.97217	-1.821607	0.000745907
AKAP2|PALM2-AKAP2	595.85498	126.427521	-2.323441	0.016509743
RHNO1	4249.5083	884.992615	-2.275817	0.000479378
FNBP1L	2532.15283	816.329407	-1.639684	0.006612241
UGT2B28|UGT2B11	913.644348	332.417175	-1.446243	0.043775395
BCKDHA	1472.54712	373.833099	-1.969372	0.005726059
ZNF7	1690.56531	408.709656	-2.04466	0.003713743
GJB1	11453.3496	2328.01025	-2.337799	0.00016139
TMEM101	8933.20605	3784.10645	-1.285817	0.009949588
HNRNPA0	2566.3335	515.519104	-2.326831	0.000878764
FIBP	1237.90027	362.934174	-1.758797	0.013018327
HSPA8	23417.5625	2664.78687	-3.188622	1.43712E-05
DNAJB6	6835.24219	925.318665	-2.910756	5.7629E-05
SUMO1|SUMO1P3	50417.6484	13618.2051	-2.047402	0.000264421
SNX5	64197.1406	19315.0723	-1.909032	0.000435651
CHMP4B	30562.2793	11535.4209	-1.513931	0.002564173
IGBP1	533.959961	159.124283	-1.795229	0.046355393

PSMC2	46783.3945	14039.9941	-1.887517	0.000492921
CCT2	39594.332	4162.29883	-3.366265	8.91661E-06
ALG1	812.949463	171.113098	-2.279883	0.009392791
BIRC5	2911.83716	1250.10657	-1.223401	0.024665531
ALDOC	2024.05933	384.732025	-2.393912	0.001148882
ADA	803.711365	246.315674	-1.703566	0.029379157
MORF4L1	1402.33777	590.72168	-1.244638	0.045076121
15-Sep	63156.9336	14830.166	-2.267455	0.000118772
IDI1	8390.00781	2804.29321	-1.62262	0.00246766
LGMN	1534.44214	115.528595	-3.826792	0.000244627
TUBA1C	37161.9531	5460.36084	-2.882132	2.35488E-05
MLF2	4056.43286	1216.31995	-1.744781	0.0027341
PRIMPOL	613.407288	159.124283	-1.994539	0.026343957
TMX2-CTNND1 |TMX2	3724.78662	515.519104	-2.869687	0.000139609
PRDX3	42441.5039	5672.88965	-3.035926	1.63187E-05
TAB2	2232.8396	980.903137	-1.1861	0.034985207
CNBP	10434.3906	3095.29443	-1.80005	0.001087409
DAZAP2	2992.20825	850.116089	-1.824468	0.002947677
CCR6	1546.45154	515.519104	-1.583971	0.014916468
CDC45	522.874329	126.427521	-2.135701	0.028022572
SF3A3	6021.36865	719.328979	-3.092033	4.65373E-05
CCDC82	1472.54712	353.125122	-2.0494	0.00470806
RNF41	667.911865	170.023209	-2.010112	0.022059536
ZNF791	361.208221	71.9329	-2.542908	0.033636458
CSDE1	2908.14185	1162.91516	-1.325149	0.017090626
CEP76	939.510925	354.215027	-1.396775	0.047534399
TSPAN12	2024.05933	753.115662	-1.428182	0.017164126
TCP11L2	3996.3855	667.01416	-2.598855	0.000225876
EIF2AK1	3857.8147	1604.32153	-1.275054	0.016429355
UTP15	1227.7384	246.315674	-2.309917	0.003771169
ANGEL2	1002.32971	277.922546	-1.834092	0.015446182
MAGT1	2177.41113	913.329834	-1.253515	0.028439598
SLC2A3	7405.23047	2383.59473	-1.670368	0.002200262
HNRNPR	16087.1611	7932.23682	-1.075257	0.021509664
RSRP1	1898.42175	418.518677	-2.181461	0.002142684
RBM45	2031.44983	902.430908	-1.169028	0.040186066
C2orf15	1147.36731	406.529846	-1.48918	0.029123161
RTFDC1	13180.8672	2815.19214	-2.266976	0.000185515
CA14	677.149963	211.439117	-1.691843	0.039203171
SRPX2	18108.4492	7583.47119	-1.311251	0.007118659
ERICH1	4547.89795	1228.30872	-1.897923	0.001433453
ARHGDIB	99526.2578	58000.8047	-1.084025	0.017451264
NSRP1	4060.12817	1863.71594	-1.13504	0.027457815

MFAP1	3381.13062	481.732422	-2.827101	0.000178046
ZCCHC10	4464.75537	2219.021	-1.026852	0.040027741
UNCX	2710.44727	1128.03857	-1.266778	0.022364153
SLC16A9	377.836731	95.91053	-2.122118	0.049920496
OR2AG1	4963.61084	1304.6012	-1.940505	0.001136182
OR10AD1	7584.44873	754.205505	-3.361687	2.26202E-05
KRTAP20-4	1284.09058	204.899765	-2.655255	0.001778611
DDOST	77327.1953	13512.4854	-2.708943	3.17202E-05
LACE1	2539.54321	992.891968	-1.357621	0.017193196
SLC25A40	1537.2135	577.642944	-1.410455	0.024961628
ALB	74848.625	26083.3047	-1.730315	0.000893741
GALT	2871.1897	1097.52161	-1.39087	0.013605996
TRMT11	2765.87573	568.923828	-2.294037	0.00084446
ILF2	15805.4004	7736.05615	-1.086065	0.020541458
FAM49B	21274.332	8428.1377	-1.405845	0.004469502
SNRNP40	3821.78613	1873.52502	-1.039635	0.041235793
YBX1	146527.672	73933.9375	-1.336031	0.005146547
AKR1B1	42895.0938	23742.2148	-1.008182	0.026104573
ID2	69704.875	36977.8672	-1.159443	0.012338034
TMEM59	53943.8164	25868.5957	-1.243481	0.008399161
SGCE	17116.2812	8437.94727	-1.074519	0.02134032
MTFR2	3803.31006	1355.82617	-1.495497	0.007180005
PRDX3	109299.203	34814.4336	-1.90385	0.00042583
SLC50A1	1961.2406	903.520813	-1.115537	0.049397212
KARS	55327.6758	29700.6582	-1.095144	0.016999833
LIAS	9874.56445	5111.59521	-1.003848	0.033004243
PKIB	12847.373	6213.47656	-1.10429	0.019767439
SDCBP	9256.53809	3796.09521	-1.333177	0.007932254
CCBL2	9837.6123	5082.16797	-1.006526	0.032650691
BTF3	8715.1875	3894.18555	-1.208722	0.01396799
RBMS1	8968.31055	3838.60107	-1.271078	0.010524525
SLC4A1AP	1644.375	719.328979	-1.190594	0.045344319
GLRX	3823.63379	1808.13147	-1.090751	0.033814482
PSMD12	19520.9492	9034.11816	-1.175941	0.016333139
SDHD	21482.1895	9332.74805	-1.275252	0.008105579
SEC22C	5990.8833	1583.61365	-1.940058	0.000955011
SMPDL3B	703.940308	202.719986	-1.811385	0.029126488
RPL7L1	38102.3867	15474.292	-1.425108	0.003715408
S100PBP	1988.03088	677.913086	-1.554663	0.011712135
LACTB	8444.5127	2502.39282	-1.793215	0.001260196
IFT74	1644.375	419.608582	-1.967463	0.004747764
FBXO4	3071.65552	1355.82617	-1.184784	0.0273035
ETNK1	2800.98047	1138.9375	-1.300697	0.019239288
PPHLN1	17974.4961	8015.06885	-1.221018	0.010778888

TM9SF1	2819.45654	1377.62402	-1.037339	0.049739186
FBXO3	1581.55615	485.002106	-1.704963	0.033738278
WDR20	929.348999	340.046417	-1.438629	0.043562975
MSI2	2194.96362	937.307434	-1.227425	0.030843921
SF3B1	8877.77734	3689.28564	-1.313051	0.008805122
ORC5	3497.53027	590.72168	-2.580895	0.000287461
CLK1	8986.78711	3612.99316	-1.360766	0.007127389
BCKDHB	2351.08667	977.633484	-1.266874	0.025347004
ADAL	1824.51721	773.823608	-1.235876	0.03560688
TAF1D	26790.3789	11914.7031	-1.261235	0.008335778
IP6K2	19158.8164	8468.46387	-1.239652	0.00976536
HACD2	434.188904	93.730743	-2.366862	0.028849628
CRIM1	2783.42798	1227.21887	-1.184289	0.029457392
MBTD1	2521.06714	859.92511	-1.557145	0.00874254
JMJD6	5252.76221	1388.52295	-1.934632	0.001096705
CEP63	2387.11523	1088.80249	-1.131738	0.039991602
MBD2	6275.41553	2329.1001	-1.460128	0.005662708
GART	712.254578	244.135895	-1.545192	0.048897337
IKBIP	6455.55762	3072.40674	-1.108985	0.024163777
PINX1	1480.86133	634.317383	-1.220638	0.045904264
VWA5A	430.493683	82.831818	-2.563111	0.023373134
GFM2	1807.88867	764.014587	-1.241067	0.035325483
IFT81	1091.93896	319.33847	-1.756283	0.016059078
SLC28A1	1300.71912	297.540619	-2.1097	0.005167836
LLGL2	1226.81458	459.934601	-1.412908	0.032626092
PCK2	11363.7402	3148.69922	-1.898105	0.000716725
MTA3	8409.4082	2781.40527	-1.637584	0.004743267
MAPKAP1	2938.62744	1065.91479	-1.467367	0.010127148
IFNAR2	3100.29346	1161.82532	-1.41954	0.011425459
AGPAT2	4094.30908	1313.32031	-1.648356	0.003822981
BCCIP	61251.1211	26062.5957	-1.428343	0.003488423
MPZL2	3047.63672	1388.52295	-1.139223	0.032513704
USP48	1455.91858	555.845093	-1.387251	0.028497737
SLC30A5	2324.29639	861.015015	-1.435781	0.014386779
PPIE	4076.75684	1929.1095	-1.092195	0.032418974
TNFRSF19	2919.22754	686.632202	-2.10012	0.001317348
MAX	1925.21216	480.642548	-2.005573	0.003288186
ITGB1	23608.791	6461.97217	-1.941405	0.000459142
KYNU	38914.4102	9161.63574	-2.220343	0.000149897
MAPK14	1853.15527	710.609863	-1.383113	0.02188427
ZEB1	912.72052	331.327271	-1.449416	0.043604188
MTERF1	3306.30225	1601.05188	-1.053626	0.042453528
GLRX3	43999.043	23212.5273	-1.078345	0.018703412
NCK1	2792.66602	1172.72424	-1.254189	0.02281224

MAD2L1	34168.8203	17818.6504	-1.046544	0.022201339
RAB4A	7351.6499	3288.20532	-1.202486	0.015357822
CXorf40B	1390.32837	222.338043	-2.646417	0.001507478
POFUT1	938.587097	147.135468	-2.727627	0.003499957
RIOK2	6725.30908	2069.70557	-1.729229	0.001871551
RBM34	7115.15527	2006.49194	-1.855703	0.001121647
LGMN	11391.4541	5134.48291	-1.203988	0.013038776
RFC3	1472.54712	623.418457	-1.237564	0.043781802
NOVA1	5713.74121	2337.81909	-1.316635	0.010781225
SAMD4A	1138.12927	398.900604	-1.504043	0.028261524
ABHD11	2024.05933	470.833496	-2.109074	0.002306456
IDS	1039.28198	371.65329	-1.472901	0.034582231
SEPP1	52324.3828	18108.5625	-1.683781	0.001128921
PTPN2	8381.69336	3828.79199	-1.176018	0.016345115
CNOT7	15651.124	6721.36621	-1.271618	0.008822489
RBM34	8550.75	2187.41406	-2.001782	0.000585088
KDELR3	11670.4434	5178.07861	-1.226157	0.011723669
GIT2	549.664673	166.75354	-1.761424	0.047027148
MTFR1	1156.60535	357.484711	-1.681377	0.017590106
PCBP1	50798.2539	17457.8965	-1.692343	0.00109184
KPNA2	47787.5703	9842.81836	-2.436552	7.24991E-05
ERCC2	560.750366	147.135468	-1.991786	0.030904187
HMGCL	6735.4707	2274.60547	-1.597902	0.003112455
PRMT1	27223.6445	13414.3955	-1.110666	0.016830953
LIMS1	15442.3447	7831.9668	-1.035741	0.025974581
MORF4L1	17385.1094	8590.53223	-1.071405	0.021587879
AMACR|C1QTNF3-AMACR	2487.81006	883.90271	-1.497397	0.010852295
AMACR	1959.39294	633.227478	-1.632096	0.009393241
FCF1	2467.48633	826.138428	-1.584171	0.008191118
PIGY|PYURF	25815.7637	12982.7979	-1.078128	0.019727184
H2AFV	4150.66113	1660.99597	-1.331715	0.012506239
PWARSN	1862.39331	461.024475	-2.017149	0.003370796
FAM127A	3390.36865	1486.61316	-1.196401	0.024345886
ATP5SL	1518.73743	190.731171	-3.008077	0.000674333
LOC389831	1074.3866	362.934174	-1.55351	0.027006449
ARMCX6	803.711365	147.135468	-2.506875	0.006607795
NBPF1|NBPF14|NBPF10	1083.62463	298.630524	-1.83956	0.013370247
MED18	983.853577	354.215027	-1.462399	0.038215883
MKRN1|MKRN7P	2133.0686	459.934601	-2.220143	0.001579436
HLA-C|HLA-B|HLA-H	9980.80176	4236.41162	-1.286059	0.009465924

PDZK1	8055.58984	4032.60181	-1.043885	0.029415216
SNRPN|SNURF	3433.7876	1335.11816	-1.368912	0.012580686
UBE2M|UBE2MP1	7150.25977	3192.29492	-1.204315	0.015420544
DNAJB6	10611.7617	4302.89502	-1.353233	0.006894573
TUBA3C|TUBA3D|TUBA3E|TUBA1A	4176.52783	1218.49963	-1.784646	0.002308334
MTCH1	2274.41089	633.227478	-1.851349	0.00392733
C1GALT1	17476.5664	6589.48926	-1.45614	0.003740784
SUMO4	79164.6484	38778.3711	-1.284641	0.006738401
WASH1|WASH3P|WASH7P|WASH2P	1263.76685	449.035675	-1.489879	0.026610158
ACOT2	4746.51611	2404.30249	-1.003599	0.042677634
HAVCR1	396.312866	93.730743	-2.231678	0.040164307
ATRAID	3517.854	1746.00757	-1.019633	0.046582677
ATRAID	2005.58325	492.631348	-2.030034	0.002877144
ALG11	6247.70117	2079.51465	-1.613962	0.003075835
HMGN3	4756.67822	2114.39111	-1.188536	0.020222524
CXADR	5595.49414	2423.92065	-1.234643	0.015296831
BMPR1A	859.139771	287.731598	-1.561956	0.039768569
ISCA1	3432.86377	1140.02747	-1.595135	0.005524853
GAPDH	236695.781	142709.422	-1.119909	0.014398824
GIGYF2	651.283386	191.82106	-1.784063	0.034767021
BORA	1182.47192	483.912231	-1.285823	0.048222344
FAM213A	23627.2676	11158.3184	-1.16532	0.013287811
HNRNPUL2	2649.47607	1010.33026	-1.394482	0.014503152
DLD	19981.0039	8590.53223	-1.283186	0.007925574
EIF4G2	3671.20581	1647.91724	-1.164533	0.026057743
SDHC	32831.1484	15247.5947	-1.214098	0.01011962
PSAT1	17746.3164	6061.98145	-1.597218	0.00201982
NA	2042.53552	741.126831	-1.465013	0.015080227
CASP7	2584.80981	503.530273	-2.371464	0.000777242
BAAT	8596.94043	4356.2998	-1.028303	0.030838262
SNX6	10587.7432	4960.1001	-1.14808	0.017100165
BZW1	34320.3242	15020.8965	-1.30556	0.006552789
NA	1264.69067	287.731598	-2.116481	0.005370418
UBAC2	1054.98669	330.237396	-1.660165	0.040251568
PDXDC1	838.81604	256.124725	-1.704972	0.027428897
TMEM242	3950.19531	1583.61365	-1.328084	0.013156354
BAG5	3969.59521	1099.70142	-1.859064	0.001888141
NA	3661.96777	1757.99646	-1.068217	0.037823007

RNF183	7142.86963	3505.09399	-1.069029	0.027519299
AS3MT|C10orf 32-ASMT	5353.45703	1055.01587	-2.358765	0.000288798
PHACTR2	2971.88452	5.449462	NA	NA
CAPZB	26014.3828	6235.27441	-2.139467	0.000217261
CACNA2D4	2331.68677	741.126831	-1.659726	0.006863077
C4orf33	3155.72192	1347.10693	-1.233295	0.02235215
SBDS|SBDSP1	21557.0176	7196.55957	-1.651083	0.00151666
SVIL	2440.69604	914.419739	-1.41964	0.014435403
NA	4925.73438	1874.61487	-1.410297	0.008121615
ASPH	4085.07104	1559.63599	-1.398771	0.009759934
LCOR	3062.41748	1247.92676	-1.299227	0.017927753
SMNDC1	5802.42676	1874.61487	-1.651735	0.002804545
PMAIP1	9168.77637	3580.29639	-1.403034	0.005892107
ALG9	2657.79053	871.91394	-1.614566	0.006785282
MAX	2431.45801	579.822754	-2.077167	0.001860068
RALB	3225.93115	473.013306	-2.785224	0.000209813
ERAL1	1113.1864	310.619324	-1.823144	0.013270048
PPIL4	8822.34863	2587.40454	-1.809829	0.001151751
PTAFR	3200.0647	1067.00464	-1.589818	0.006022556
SMUG1	2214.36353	718.239075	-1.629529	0.008069299
LANCL1	1309.95715	555.845093	-1.233803	0.049905803
KIF4A|KIF4B	1029.12012	361.844269	-1.496481	0.033045322
ZCCHC9	13862.6357	5599.86719	-1.358245	0.006170649
TOE1	1942.7644	859.92511	-1.173939	0.04109272
GJB1	7123.46973	2339.99902	-1.639872	0.002542771
NKIRAS1	6898.06104	2231.00977	-1.660425	0.002396251
NMNAT3	2107.2019	869.734131	-1.27684	0.027117075
ACVR1	868.377808	224.517838	-1.954692	0.01528734
CSGALNACT2	1156.60535	439.226624	-1.392191	0.037130997
ELF3	379.684357	51.224941	-3.238096	0.016618226
NEK2	2224.52539	447.94577	-2.319162	0.001148317
BZW1	17848.8594	6506.65771	-1.506072	0.00450336
PGM3	15884.8477	5544.28271	-1.564245	0.002398425
LARP4	2703.98071	1302.42139	-1.056718	0.047664736
FCGR3B|FCG R3A	4030.56641	1335.11816	-1.60195	0.004595107
LOC391003|PR AMEF19	1682.2511	526.41803	-1.675925	0.010175272
DEFB131|LOC 100129216	4130.3374	1379.80371	-1.590338	0.004689145
HNRNPCL3|H NRNPCL2|HN RNPCL1	26573.2852	8264.6543	-1.772227	0.000869695

SERF1A|SERF1B	4755.75439	2101.3125	-1.197028	0.019542908
SRSF10	15442.3447	7142.06494	-1.167002	0.014258089
ZFAND6|ATP8B5P	25987.5918	5685.96875	-2.268063	0.000140999
ZFAND6|ATP8B5P	27877.6992	14677.5811	-1.014633	0.026297979
PLGLB2|PLGLB1	12356.832	4518.69385	-1.499223	0.003455041
DDX11|LOC642846|DDX12P	525.645752	158.034393	-1.783753	0.048424512
RDH14|NT5C1B-RDH14	6228.30127	1831.01917	-1.789421	0.00158286
THOC3|LOC728554	54116.5664	18961.9473	-1.671926	0.001183651
CDK11B|CDK11A	1687.79395	481.732422	-1.809195	0.006947176
TRIM74|TRIM73|TRIM50	1896.5741	590.72168	-1.684823	0.008365598
TRIM73|TRIM74|TRIM50	1518.73743	515.519104	-1.557704	0.016484425
CCDC74B|CCDC74A	2250.39185	1044.11694	-1.106283	0.045707781
FAM72A|FAM72B|FAM72D	10106.4395	5240.20264	-1.002649	0.032980945
GUSBP3|GUSBP4|GUSBP2|GUSBP1|SMA5|GUSBP9|GUSB	5433.82812	1743.82788	-1.658118	0.002880272
RSL24D1	94852.7266	49444.0586	-1.22844	0.008737558
CDKN2AIPNL	3615.77734	1238.1178	-1.552093	0.00610528
CSNK2A3|CSNK2A1	3108.60791	1105.15088	-1.49628	0.008643473
SETSIP|SET	2287.34424	321.51825	-2.825821	0.000369406
CTAGE9|CTAGE15|CTAGE6|CTAGE8|CTAGE4	848.054077	223.427933	-1.928398	0.016835116
POTEF|POTEJ|POTEM|ACTB|POTEE	87210.0781	34752.3086	-1.572575	0.001761446
POTEF|POTEJ|POTEI|POTE	24656.3867	6236.36426	-2.057633	0.000294745

M|POTEE				
POTEF|POTEJ |POTEI|POTE M|POTEE	18183.2773	4516.51416	-2.049888	0.000334631
ACTG1	119887.875	49719.8008	-1.569963	0.001751975
SEC13	23191.2305	9721.83984	-1.333496	0.00611072
SEC13	27674.4629	10283.1348	-1.522762	0.002508835
SYNGR2	6066.63525	1712.22095	-1.846699	0.001312493
LOC101929087	1382.01404	384.732025	-1.836849	0.008915031
NA	8109.1709	1918.21057	-2.110197	0.000419756
NA	776.921021	234.326859	-1.731258	0.029216163
NA	4889.70605	883.90271	-2.484006	0.000229301
NA	739.044922	202.719986	-1.880634	0.023752663
NA	4593.16455	680.092834	-2.774211	0.000124902
NA	13145.7617	827.228333	-4.019931	5.94422E-06
NA	19961.6035	5544.28271	-1.902225	0.000560802
NA	2089.64966	481.732422	-2.122827	0.002100789
NA	6925.7749	2328.01025	-1.606066	0.002959314
NA	3904.92871	1692.60291	-1.215954	0.020507049
NA	7667.59131	3126.90137	-1.335877	0.008517321
NA	867.454041	156.944504	-2.51149	0.00553115
NA	9863.47852	2685.49487	-1.919979	0.000714543
NA	11816.4053	5846.18262	-1.072391	0.023296392
NA	1934.4502	601.620605	-1.687422	0.008075701
NA	6915.61328	1137.84766	-2.625571	0.000109136
NA	1708.11768	297.540619	-2.505477	0.001268705
TNFAIP8	8786.32031	4274.55811	-1.087211	0.023695601
SIGMAR1	1607.42273	643.036499	-1.319982	0.031858437
PDIA5	7124.39355	1182.5332	-2.613457	0.000109243
TMEM116	1988.03088	765.104431	-1.378672	0.020557592
ADM	25741.8594	8976.35352	-1.606054	0.001765306
PIGB	7413.54492	3365.58765	-1.181433	0.016751794
MRFAP1L1	27704.0234	14006.207	-1.073968	0.019946346
UBC|UBA52	149545.75	93501.8672	-1.036252	0.021716073
CDC25C	1824.51721	602.71051	-1.598756	0.01142396

“a” refers to the group treated with siCtr RNA.

“b” refers to the group treated with siPCNAP1.

“Downreg^{gene}” refers to the genes downregulated by siPCNAP1.

Table S6. List of siRNAs used in this paper

Gene		Sequence (5′-3′)
siPCNA-human-1	Sense	GGAGAAAGUUUCAGACUAU
siPCNA-human-2	Sense	GAUCGAGGAUGAAGAAGGA
siPCNAP1-1	Sense	GCACUAAAUCAAGAGAACU
siPCNAP1-2	Sense	GAGUACAGCUGUGUAAUAA
siPCNAP1-3	Sense	CCUAGAGAGAUAUCUGCUU
siHBc	Sense	GAUCUCAAUCUCGGGAAUCUCA
